# The epidemiologic impact and cost-effectiveness of new tuberculosis vaccines on multidrug-resistant tuberculosis in India and China

**DOI:** 10.1186/s12916-021-01932-7

**Published:** 2021-02-26

**Authors:** Chathika K Weerasuriya, Rebecca C Harris, C Finn McQuaid, Fiammetta Bozzani, Yunzhou Ruan, Renzhong Li, Tao Li, Kirankumar Rade, Raghuram Rao, Ann M Ginsberg, Gabriela B Gomez, Richard G White

**Affiliations:** 1 TB Modelling Group, TB Centre and Centre for the Mathematical Modelling of Infectious Diseases, Department of Infectious Disease Epidemiology, Faculty of Epidemiology & Population Health, London School of Hygiene and Tropical Medicine, London, UK; 2 Currently employed at Sanofi Pasteur, Singapore, Singapore; 3 Department of Global Health and Development, Faculty of Public Health & Policy, London School of Hygiene and Tropical Medicine, London, UK; 4 Chinese Centre for Disease Control and Prevention, Beijing, China; 5 World Health Organisation, New Delhi, India; 6 National Tuberculosis Elimination Programme, New Delhi, India; 7 International AIDS Vaccine Initiative, New York, USA; 8 Current Affiliation: Bill and Melinda Gates Foundation, Washington DC, USA; 9 Currently employed at Sanofi Pasteur, Lyon, France

**Keywords:** Tuberculosis, Drug resistance, Vaccine, Mathematical model

## Abstract

**Background:**

Despite recent advances through the development pipeline, how novel tuberculosis (TB) vaccines might affect rifampicin-resistant and multidrug-resistant tuberculosis (RR/MDR-TB) is unknown. We investigated the epidemiologic impact, cost-effectiveness, and budget impact of hypothetical novel prophylactic prevention of disease TB vaccines on RR/MDR-TB in China and India.

**Methods:**

We constructed a deterministic, compartmental, age-, drug-resistance- and treatment history-stratified dynamic transmission model of tuberculosis. We introduced novel vaccines from 2027, with post- (PSI) or both pre- and post-infection (P&PI) efficacy, conferring 10 years of protection, with 50% efficacy. We measured vaccine cost-effectiveness over 2027–2050 as USD/DALY averted-against 1-times GDP/capita, and two healthcare opportunity cost-based (HCOC), thresholds. We carried out scenario analyses.

**Results:**

By 2050, the P&PI vaccine reduced RR/MDR-TB incidence rate by 73% (UI:66–76) and 72% (UI:65–77), and the PSI vaccine by 29% (UI: 27–31) and 47% (UI: 37–58) in China and India, respectively.

In India, we found both USD 10 P&PI and PSI vaccines cost-effective at the 1-times GDP and upper HCOC thresholds and P&PI vaccines cost-effective at the lower HCOC threshold. In China, both vaccines were cost-effective at the 1-times GDP threshold. P&PI vaccine remained cost-effective at the lower HCOC threshold with 49% probability and PSI vaccines at the upper HCOC threshold with 21% probability. The P&PI vaccine was predicted to avert 1.0 million (UI: 0.6–1.3) and 0.8 million (UI: 0.5–1.4) second-line therapy regimens in China and India between 2027 and 2050, respectively.

**Conclusions:**

Novel TB vaccination is likely to substantially reduce the future burden of RR/MDR-TB, while averting the need for second-line therapy. Vaccination may be cost-effective depending on vaccine characteristics and setting.

**Supplementary Information:**

The online version contains supplementary material available at 10.1186/s12916-021-01932-7.

## Background

Rifampicin-resistant and multidrug-resistant tuberculosis (RR/MDR-TB) threatens to impede global tuberculosis (TB) control efforts and progress towards the World Health Organization End TB targets [1], with approximately half a million incident cases in 2018 [2]. RR/MDR-TB has worse treatment outcomes than drug-susceptible TB (DS-TB) and imposes a disproportionate cost on health systems and patients [3, 4]. Further, prolonged multi-agent treatment for RR/MDR-TB may contribute to wider antimicrobial resistance [5]. As such, there is an urgent need for novel interventions to control and prevent RR/MDR-TB.

Prophylactic TB vaccine candidates progressed considerably through the development pipeline in 2018–2019. Results from the M72/AS01_E_ [6] and BCG revaccination [7] trials suggest possible vaccine efficacy of 50% and 46% in Interferon-γ release assay (IFNy) + and IFNy-negative patients, respectively. These trials did not include RR/MDR-TB endpoints due to its relative rarity.

Mathematical modelling techniques could investigate the potential effect of vaccination on RR/MDR-TB. However, to date, no modelling studies have assessed the epidemiologic impact of novel TB vaccines on RR/MDR-TB. Furthermore, studies of TB vaccine cost-effectiveness have omitted RR/MDR-TB or not modelled RR/MDR-TB dynamically [8, 9].

The global distribution of RR/MDR-TB is heterogenous, with India and China accounting for 27% and 14% of all global cases, respectively [2]. High-quality national RR/MDR-TB burden estimates are derived through large and infrequent drug-resistance surveys; consequently, data to inform drug resistance trends are sparse. In 2017, globally, 3.5% (95% CI 2.5–4.7%) of new and 18% (95% CI 6.3–34%) of previously treated TB cases were estimated to have RR/MDR-TB [10]. Data from China indicate slightly higher rates, with estimates of 7.1% (95% CI 5.6–8.7) and 24% (95% CI 20–24) RR/MDR-TB among new and previously treated cases. Data from India indicate slightly lower-than-global rates. Estimates from the first national drug-resistance survey, reporting in 2018, place RR/MDR-TB among new and previously treated cases at 2.8% (CI 2.3–3.5) and 11.6% (10.2–13.2), respectively. Approximately 69% and 74% of RR-TB cases are estimated to be MDR-TB (defined as resistance to isoniazid in addition to rifampicin) in India and China, respectively, consistent with the global average of 78%. Despite these similarities, China and India have substantially differing demographics, TB epidemiology and health systems [2, 11, 12]. As such, in this study, we modelled the epidemiologic impact, cost-effectiveness and budget impact of prophylactic vaccination against TB, while dynamically modelling epidemics of DS- and RR/MDR-TB in China and India.

## Methods

### Model structure and calibration

We programmed an age-, treatment history- and drug resistance-stratified compartmental transmission model of TB in R [13], extending previously developed methods [8, 14, 15]. Details of model structure, diagram, equations, calibration, vaccine implementation, demography and health economic analysis are described in Additional file 1 [2, 7, 8, 11–14, 16–101]. The model time horizon was 1950–2050.

The model allowed for five states of TB disease: (1) susceptible; (2) latently infected; (3) active disease (both infectious, i.e., bacteriologically sputum-positive, and non-infectious, i.e., bacteriologically sputum-negative, and extra-pulmonary); (4) on-treatment; and (5) recovered from disease, stratified by drug-susceptible/drug-resistant, and treatment history (Fig. S1 in Additional file 1). All states were stratified by vaccination status. Transitions between states represented acquisition of infection, progression to latency or active disease, conversion of non-infectious to infectious active disease, detection of active disease and initiation of treatment, treatment success or failure, reactivation from latency and relapse from recovered states. Misdiagnosis of RR/MDR-TB was assumed to lead to inappropriate initiation of DS-TB treatment and treatment failure. Consistent with empirical data, progression to active disease following re-infection of latent and recovered populations was assumed to occur at a lower rate than naive populations [42]. We assumed that RR/MDR-TB could develop following drug resistance acquisition in situ while on treatment for DS-TB or following transmission of drug-resistant *M. tuberculosis*. Transmission could occur between drug-resistance strata. We assumed resistance acquisition and RR/MDR-TB treatment began in 1970.

**Fig. 1:**
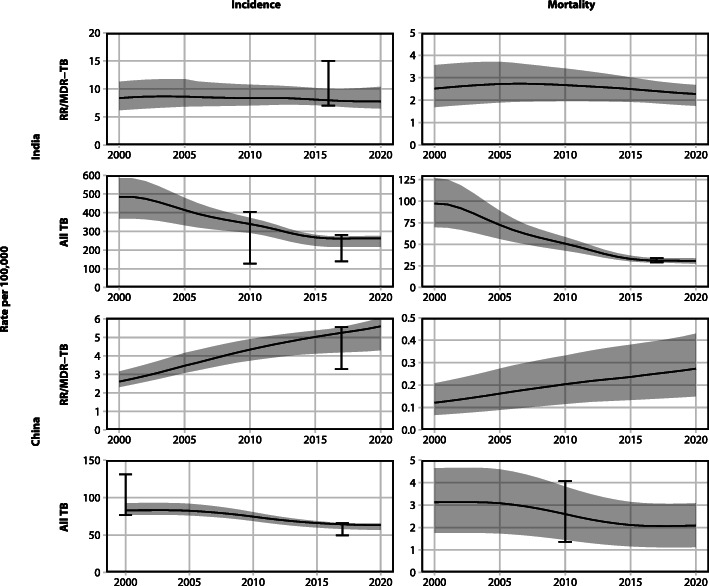
Model calibration: TB incidence and mortality of RR/MDR-TB and All TB in India and China. Lines represent median model trajectory. RR/MDR-TB mortality presented for comparison–no calibration targets available. Ribbons represent model uncertainty. Error bars represent calibration targets and uncertainty. Note: *y*-axis scales on subplots differ

Neonates entered the model uninfected. We modelled 0–100 years in 1-year age groups and applied all-cause mortality to all states, with TB-specific mortality applied to active-disease and on-treatment states. We applied age-assortative contact patterns using empirical data for China [39] and by adapting POLYMOD contact matrices [40] for India.

We obtained prior ranges for natural history parameters from the literature (Additional file 1, section 2.4), including age-stratified ranges where available. We assumed RR/MDR-TB was less than or equally transmissible to DS-TB [28, 29]. We constrained rates of fast progression to active disease, reactivation from latent infection, relapse from recovered state and TB mortality to be greater or equal in children (age < 15 years) than adults [21, 47]; in China, we also constrained these rates to be greater or equal in the elderly (age > 64 years) than in adults (age 15–64). We applied the opposite constraint to natural cure rate [21, 47].

We used country-specific case-detection rates to inform diagnosis and treatment initiation [31] (Additional file 1, section 2.5). RR/MDR-TB was diagnosed through a combined probability of drug-susceptibility testing (determined by country-specific drug-susceptibility testing coverage) and empirical diagnosis (Additional file 1, section 2.5). We based treatment success for DS-TB and RR/MDR-TB treatment on historical data [31, 58]. In India, we adjusted case-detection and DS-TB treatment success rates for a large private healthcare sector [2, 11, 32], where we assumed a lower treatment success rate for DS-TB and unsuccessful treatment of RR/MDR-TB [Rao, R., *personal communication*]. Beyond 2018, we maintained constant rates of treatment initiation, drug-susceptibility testing coverage and treatment success.

We calibrated the model to historical rates of all TB prevalence, incidence, notification and mortality [2, 34, 35, 69], RR/MDR-TB incidence rate and percentage RR/MDR-TB among notifications (stratified by treatment history) in each country. In India, we fitted to total RR/MDR-TB treatment initiations in the public sector. In China, we constrained the number of RR/MDR-TB treatments contributing to cost-effectiveness calculations to the number of laboratory-confirmed RR/MDR-TB treatment initiations reported by China Centres for Disease Control [31]. Where data allowed, we stratified calibration targets by age group (< 15, 15–64 and ≥ 65 years in China, and < 15 and ≥ 15 years in India).

Model calibration used Approximate Bayesian Computation (ABC) rejection-sampling process and ABC Markov chain Monte Carlo sampling. We randomly subsampled 1000 fully calibrated parameter sets to generate model runs, whose median trajectory we used as an estimate of central tendency and whose maximum and minimum trajectories represent uncertainty intervals.

### Vaccine implementation

We simulated vaccine introduction in 2027 for each country and estimated vaccine impact for 2027–2050 by comparison to the corresponding unvaccinated baseline model runs.

We modelled two simultaneous vaccination strategies: routine annual vaccination and mass vaccination campaigns. We assumed routine annual vaccine administration to 9-year-olds, co-delivered with human papillomavirus (HPV) vaccine, with coverage of 80%, based on HPV vaccine coverage in South Africa and secondary school enrolment ratios in China and India. Mass campaigns were delivered 10-yearly to ages ≥ 10 at 70% coverage based on existing data for Menafrivac mass campaigns delivered to 1–29-year-olds. We delivered vaccination to populations in the model who had neither active disease or nor were receiving treatment, assuming no pre-immunisation testing for latent TB [73]. No other targeting or eligibility criteria were applied. We assumed a prevention of disease vaccine, priced at US$10, conferring 50% efficacy for 10 years, with vaccine efficacy in individuals with a previous history of M. tb infection (“post-infection”; PSI) or irrespective of infection (“pre- and post-infection”; P&PI). Vaccine was modelled as a reduction in the rates of progression to disease following infection, reactivation from latency and relapse from the recovered state. The reduction was proportional to vaccine efficacy. A lower burden of active disease further depressed the force of infection, leading to lower rates of M. tb transmission. We modelled vaccination as equally protective against DS-TB and RR/MDR-TB and in those with or without previous treatment history for either. In addition to the direct prevention of disease mechanism (above), vaccine reduced RR/MDR-TB burden indirectly by reducing DS-TB burden. Reduced DS-TB burden translated to reduced total patient-time on treatment for DS-TB, leading to lower drug-resistance acquisition. Vaccine waning was implemented as instantaneous at the end of duration of protection. We did not explicitly represent existing Bacillus Calmette–Guérin (BCG) immunisation programmes as we assumed protection conferred by BCG to be reflected in calibration targets.

As measures of vaccine impact, we calculated percentage reduction in incidence rate and mortality rate, in vaccine scenarios in 2030 and 2050, compared to baseline and corresponding number of cumulative averted TB cases and deaths.

### Cost, cost-effectiveness and budget impact

We estimated costs from a public sector perspective using an ingredients approach. Unit cost assumptions, estimates and full references are provided in Additional file 1, section 5. Briefly, we calculated costs incurred by the vaccine and TB programmes.

We estimated three categories of intervention (vaccine programme) costs: vaccine, delivery and programme costs. We modelled vaccines priced at US$10 as the base case. We estimated US$1.13–2.40 (routine) or US$1.20–2.47 (mass campaign) delivery cost per person vaccinated in India, and US$1.60–2.80 for both routine and mass campaign delivery cost in China. Programme costs included mass campaign organisation and management, which we estimated from the literature at US$25,374,949 per campaign in India, and US$16,133.10 per 10,000 persons vaccinated per campaign in China.

TB programme costs represented service costs for TB diagnosis (including drug-susceptibility testing) and treatment (Additional file 1, section 5.1). For India, we added costs representing nutritional support payments to patients and government incentives to improve TB case notification in the private healthcare sector. We inflated historic cost-data to 2018 values where appropriate.

We calculated incremental costs of vaccination as the difference in total costs predicted between vaccine and corresponding baseline scenario.

Using standardised outputs from the model (deaths due to DS and RR/MDR-TB by age and year and time spent with active DS and RR/MDR-TB disease), we calculated total (DS-TB and RR/MDR-TB) disability-adjusted life years (DALYs) averted by vaccination. We applied disability weights per the Global Burden of Disease study [95] and life expectancy from the UN World Population Prospects [38]. Future costs and DALYs averted were discounted at 3%.

We calculated incremental cost-effectiveness ratios (ICERs) for the 1000 vaccine runs and constructed cost-effectiveness acceptability curves for each vaccine profile through a probabilistic sensitivity analysis. Input costs were sampled from their corresponding uncertainty ranges and attached to each vaccine run. We report the proportion of ICERs which fall below three illustrative thresholds per country: 1-times 2018 World Bank gross domestic product (GDP) per capita and the lowest and highest healthcare opportunity cost (HCOC) thresholds estimated by Ochalek et al. [101].

We present budget impact for immunisation and TB programmes separately. For the immunisation programme, we present total costs incurred by instantaneous deployment of vaccine. For the TB programme, we present annual total costs for programmatic management of TB. Health economic analysis was undertaken in line with the Consolidated Health Economic Evaluation Reporting Standards [102] (Additional file 2).

### Scenario analysis

We conducted scenario analyses in two areas: product related, pertaining to vaccine characteristics and cost, and baseline related, pertaining to uncertainty in programmatic (non-vaccine) TB management and associated future health system investments.

Under product-related scenario analysis, we modelled vaccines with 30%, 70% and 90% efficacy, 5-years duration of protection, 30% mass campaign coverage and a vaccine price of US$30. There are no vaccine candidates in advanced development that prevent disease solely in uninfected (i.e., pre-infection) populations. Therefore, we present vaccines effective pre-infection (PRI) vaccines as a scenario analysis.

For baseline-related uncertainty, to capture the impact of vaccination in the context of uncertainty in future health system investments, and in contrast to the baseline scenario with no programmatic change after 2018, we defined an alternative “Policy” scenario, representing a scaled-up programmatic TB management for each country (Additional file 1, section 2.6).

For China, the Policy scenario was informed by country expert opinion. It included linearly scaling up drug-susceptibility testing coverage to 90% by 2036 and introduction of a standard 9-month RR/MDR-TB treatment regimen (with the same treatment success rate), linearly increasing this to 40% of all second-line therapy by 2036 [*Li, R., personal communication*].

For India, the National Strategic Plan of the Indian Revised National Tuberculosis Control Programme [36] informed the Policy scenario, defined as (1) increased case detection rate (combined across private and public sectors) to 85%, (2) increased drug-susceptibility testing coverage among public sector notifications to 100% and (3) increased proportion of notifications originating from the private sector to 35%, all by 2025.

### Model uncertainty

The final estimates of uncertainty in the results reflect a combination of epidemiologic input parameter uncertainty (delineated through sampling during calibration) and cost input uncertainty (incorporated through sampling during the probabilistic sensitivity analysis for cost-effectiveness analysis).

### Role of the funder

The study funder was involved in developing the research question and commented on the draft manuscript, but had no role in study design, data collection, analysis, interpretation, nor writing the initial draft. The corresponding author had full access to all study data and materials and had final responsibility for the decision to submit for publication.

## Results

### Calibration

We calibrated to all TB prevalence, incidence, notification and mortality, and to RR/MDR-TB specific rates of incidence, proportion among all TB notifications (stratified by treatment history) and number of treatment initiations (Fig. 1; further details in Additional file 1, section 6). The model predicted a RR/MDR-TB incidence and mortality rate per 100,000 in 2018 of 7.8 (UI: 6.7–10.1) and 2.4 (UI: 1.8–2.8) in India, respectively, and 5.4 (UI: 4.2–5.7) and 0.3 (UI: 0.1–0.4) in China, respectively (Fig. 1, rows 1 and 3). Baseline epidemiologic projections (without vaccine) are provided in Additional file 1, section 6. The model predicted that RR/MDR-TB incidence in China was predominantly driven by infection of susceptible (naive) individuals. In contrast, RR/MDR-TB incidence in India was driven by equal proportions of new infection of susceptible and re-infection of latently infected individuals (Additional file 1, section 7.2).

### Epidemiologic impact

**Fig. 2:**
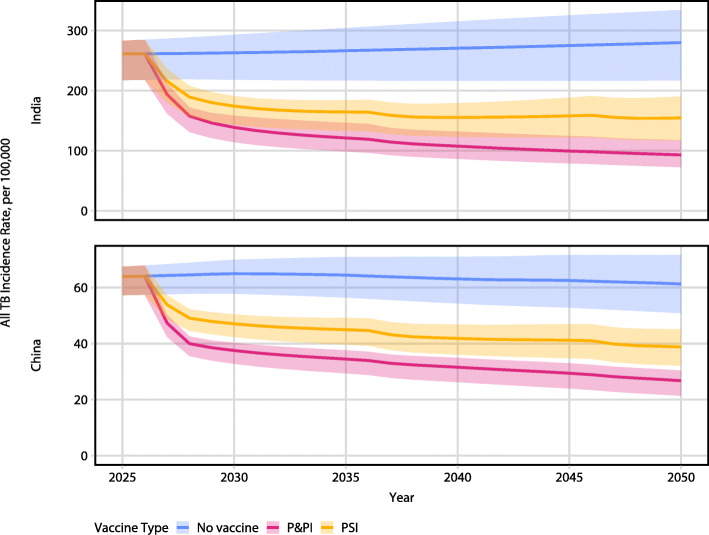
Incidence rate reduction of all TB by 50% efficacy, 10-year duration of protection pre- and post-infection efficacy (P&PI) and post-infection efficacy (PSI) vaccines in India (top) and China (bottom). Lines represent median model incidence rate; ribbons represent model uncertainty. Vaccine is introduced in 2027. Note: *y*-axis scales on subplots differ

**Fig. 3:**
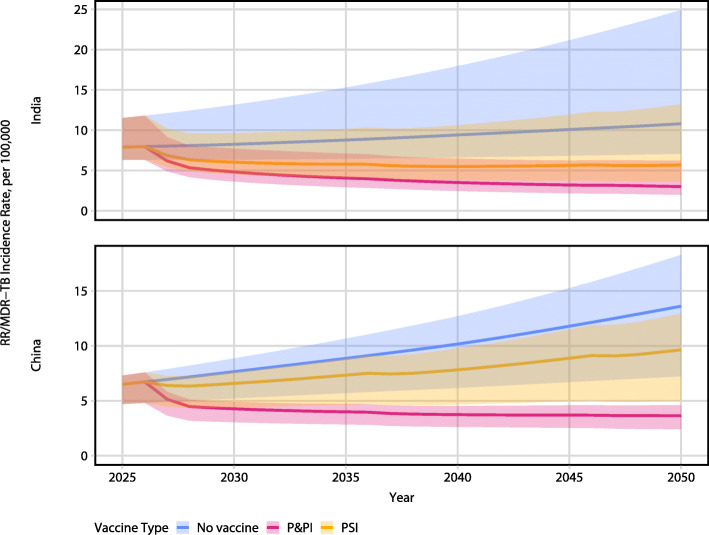
Incidence rate reduction of RR/MDR-TB by 50% efficacy, 10-year duration of protection pre- and post-infection efficacy (P&PI) and post-infection efficacy (PSI) vaccines in India (top) and China (bottom). Lines represent median model incidence rate; ribbons represent model uncertainty. Vaccine is introduced in 2027

A summary of the epidemiologic impact of both P&PI and PSI vaccines is presented in Figs. 2 and 3 and Tables 1 and 2.

**Table 1:** India–Epidemiologic impact for 50% efficacy, 10-year duration of protection vaccines. Estimates are median (uncertainty interval) values. Incidence and mortality rate reductions compare annual values of vaccine vs baseline in 2030 and 2050. Cases and deaths averted are cumulative over 2027–2030 and 2027–2050

		Pre- and post-infection vaccine		Post-infection vaccine	
Resistance status	Outcome	2030	2050	2030	2050
RR/MDR-TB	% Incidence rate reduction in year	42% (37–45)	72% (65–77)	27% (20–34)	47% (37–58)
	% Mortality rate reduction in year	20% (15–24)	69% (60–75)	13% (8–19)	45% (33–55)
	Cumulative cases averted, millions	0.2 (0.1–0.3)	2.0 (1.4–4.1)	0.1 (0.1–0.2)	1.3 (0.9–2.6)
	Cumulative deaths averted, millions	0.015 (0.008–0.02)	0.4 (0.3–0.7)	0.009 (0.004–0.015)	0.3 (0.2–0.4)
All TB	% Incidence rate reduction in year	47% (41–51)	67% (59–71)	34% (28–39)	44% (39–49)
	% Mortality rate reduction in year	36% (28–41)	66% (59–71)	25% (18–31)	44% (38–49)
	Cumulative cases averted, millions	6.1 (5.0–7.0)	57.1 (45.9–70.0)	4.3 (3.3–5.2)	39.6 (31.4–48.2)
	Cumulative deaths averted, millions	0.4 (0.3–0.5)	5.9 (4.7–7.9)	0.3 (0.2–0.3)	4.1 (3.0–5.3)

**Table 2:** China–Epidemiologic impact for 50% efficacy, 10-year duration of protection vaccines. Estimates are median (uncertainty interval) values. Incidence and mortality rate reductions compare annual values of vaccine vs baseline in 2030 and 2050. Cases and deaths averted are cumulative over 2027–2030 and 2027–2050

		Pre- and post-infection vaccine		Post-infection vaccine	
Resistance status	Outcome	2030	2050	2030	2050
RR/MDR-TB	% Incidence rate reduction in year	44% (42–46)	73% (66–76)	14% (13–16)	29% (27–31)
	% Mortality rate reduction in year	22% (18–24)	67% (59–72)	8% (6–10)	28% (25–30)
	Cumulative cases averted, millions	0.2 (0.1–0.2)	2.1 (1.1–2.7)	0.05 (0.04–0.06)	0.7 (0.5–0.9)
	Cumulative deaths averted, millions	0.003 (0.001–0.005)	0.1 (0.0–0.2)	0.001 (0.001–0.002)	0.04 (0.02–0.06)
All TB	% Incidence rate reduction in year	42% (40–44)	56% (53–59)	28% (26–29)	37% (35–38)
	% Mortality rate reduction in year	29% (26–32)	53% (48–58)	21% (18–24)	35% (33–36)
	Cumulative cases averted, millions	1.4 (1.2–1.5)	10.5 (8.9–12.0)	0.9 (0.8–1.0)	6.9 (5.9–7.8)
	Cumulative deaths averted, millions	0.02 (0.01–0.04)	0.4 (0.2–0.5)	0.02 (0.01–0.03)	0.3 (0.1–0.4)

In India, we found the P&PI vaccine reduced the RR/MDR-TB incidence rate in 2050 by 72% (UI: 65–77), corresponding to 2.0 (UI: 1.4–4.1) million cases averted (Table 1, Fig. 2). The PSI vaccine reduced the RR/MDR-TB incidence rate in 2050 by 47% (UI: 37–58), corresponding to 1.3 (UI: 0.9–2.6) million cases averted (Table 1, Fig. 2). The P&PI and PSI vaccines reduced all TB incidence rate in 2050 by 67% (UI: 59–71) and 44% (UI: 39–49), respectively (Table 1, Fig. 3).

In China, we found the P&PI vaccine reduced the RR/MDR-TB incidence rate in 2050 by 73% (UI: 66–76), corresponding to 2.1 (UI: 1.1–2.7) million cases averted (Table 2, Fig. 2). The PSI vaccine reduced the RR/MDR-TB incidence rate in 2050 by 29% (UI: 27–31), corresponding to 0.7 (UI: 0.5–0.9) million cases averted (Table 2, Fig. 2). The P&PI and PSI vaccines reduced all TB incidence rate in 2050 by 56% (UI: 53–59) and 37% (UI: 35–38), respectively (Table 2, Fig. 3).

We found a similar relative effect of P&PI vaccines compared to PSI vaccines on RR/MDR-TB mortality rate and deaths averted, and on all TB mortality rate, and cases and deaths averted (Tables 1 and 2).

### Averted treatment

In India, P&PI and PSI vaccines were predicted to avert 0.8 (UI: 0.5–1.4) million and 0.5 (UI: 0.3–1.1) million RR/MDR-TB treatment regimens (not shown). In China, the model predicted the P&PI and PSI vaccines would avert 1.0 (UI: 0.6–1.3) million and 0.3 (UI: 0.2–0.4) million RR/MDR-TB treatment regimens, respectively (not shown).

### Cost-effectiveness

**Fig. 4:**
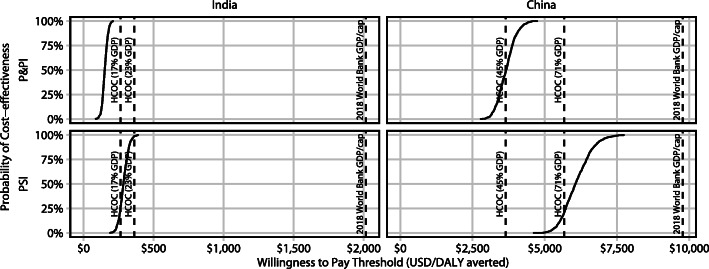
Cost-effectiveness acceptability curves. Vertical axis shows the probability that 50% efficacy, 10-year duration of protection pre-and post-infection (P&PI; top) and post-infection (PSI; bottom) vaccines are cost-effective at or below a given willingness to pay value (horizontal axis), in India (left) and China (right). Reference lines are 2018 World Bank GDP and upper and lower health-care opportunity-cost thresholds. Note: *x*-axis scales differ between subplots

In India, in a discounted analysis, we estimated ICERs of $151 (UI: 82–210) and $284 (UI: 189–389) for P&PI and PSI vaccines priced at USD 10, respectively (Fig. 4), over 2027–2050. The P&PI vaccine was predicted to be cost-effective in 100% of model runs at the 1-times GDP, and upper and lower HCOC thresholds. The PSI vaccine was predicted to be cost-effective in 100%, 99% and 27% of runs at the 1-times GDP, upper HCOC and lower HCOC thresholds, respectively.

In China, in a discounted analysis, we estimated ICERs of $3663 (UI: 2763–4754) and $6059 (UI: 4591–7749) for P&PI and PSI vaccines priced at USD 10, respectively (Fig. 4), over 2027–2050. The P&PI vaccine was predicted to be cost-effective in 100% of runs at the 1-times GDP threshold and upper HCOC threshold, and 49% of runs at the lower HCOC threshold. The PSI vaccine was cost-effective at 100% and 21% of runs at the 1-times GDP and upper HCOC threshold, but not cost-effective at the lower HCOC threshold.

### Budget impact

**Fig. 5:**
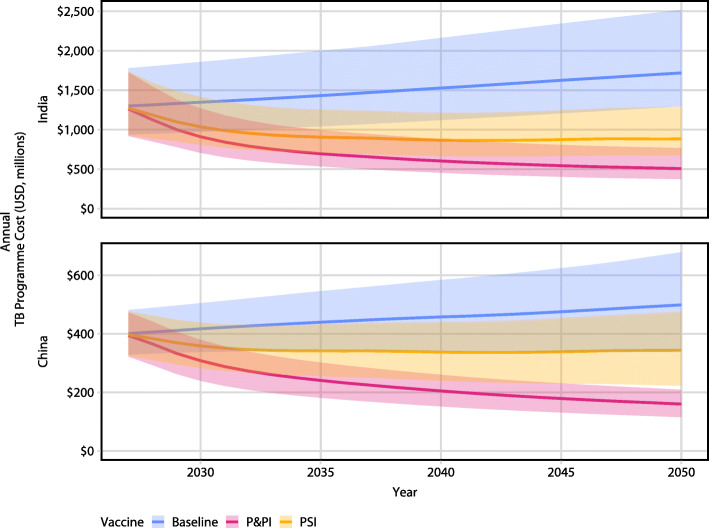
Budget impact analysis. Annual expenditure by the national TB programmes TB-related costs. Vertical axis represents annual expenditure, in millions USD, by the national TB programme, with and without vaccine, in India (top) and China (bottom). Lines represent median modelled expenditure; ribbons represent model uncertainty

The total undiscounted costs for instantly deployed mass campaigns and routine annual vaccination for a 50% efficacy P&PI vaccine providing 10 years of protection and total savings in the TB programme over 2027–2050 are presented in Table 3. Immunisation programme costs were similar for a PSI vaccine priced at US$10 in India and China (Additional file 1, section 9) but with lower TB programme savings. The total annual expenditure by the National Tuberculosis Programmes for India and China over 2027–2050 is shown in Fig. 5.

**Table 3:** Estimated cumulative total cost of vaccine programmes and cumulative TB programme savings over 2027–2050. Costs are presented for a 50% efficacy P&PI vaccine providing 10-years of protection. All costs are undiscounted and in billions USD

	Type	Amount
India	Routine vaccination costs	$5.2 (4.9–5.4)
	Mass vaccination costs	$33.4 (31.9–34.6)
	All vaccination programme costs	$38.6 (37.1–39.9)
	TB Programme savings	$19.4 (13.0–27.2)
China	Routine vaccination costs	$3.4 (3.2–3.5)
	Mass vaccination costs	$38.1 (36.4–39.2)
	All vaccination programme costs	$41.5 (39.8–42.6)
	TB programme savings	$5.2 (3.9–6.8)

### Scenario analyses

We found increasing vaccine efficacy increased percent incidence rate reduction, cases averted, percent mortality rate reduction, deaths averted in both RR/MDR-TB and all TB, and averted DS-TB and RR/MRD-TB treatment regimens. Reduced duration of protection to 5 years or reduced vaccine efficacy (Additional file 1, sections 7.3 and 7.4) had the opposite effect. PRI vaccines had a comparable or lower impact than PSI vaccines in all cases except for RR/MDR-TB in China, where PRI effect was greater than PSI (Additional file 1, sections 7.2 and 7.3).

We found vaccines (of all types, efficacies and durations of protection) affected a similar per cent incidence rate reduction and per cent mortality rate reduction in both all TB and RR/MDR-TB, in both India and China, in the Policy scenario as compared to the unchanged baseline. Fewer cases and deaths were averted in the Policy scenario, leading to a lower absolute impact of vaccination. ICERs for vaccination were higher in undiscounted analyses, with a vaccine priced at US$30, and in the Policy scenario for each country (Additional file 1, section 8).

## Discussion

We estimate that the introduction of new TB vaccines in India and China in 2027 might substantially reduce RR/MDR-TB burden by 2050. A pre- and post-infection vaccine (effective in all individuals, irrespective of their infection status by M. tb) was projected to reduce RR/MDR-TB incidence rate by approximately 70% in both India and China if delivered annually to 9-year-olds and every 10 years to ages 10 and above. A post-infection vaccine (effective only in individuals with latent M. tb infection or who have recovered from TB) was projected to impart lower but still substantial reductions of approximately 50% and 30% RR/MDR-TB incidence rate reduction in India and China, respectively.

P&PI vaccines priced at US$10 were highly likely to be cost-effective in India and China at the 1-times GDP and upper HCOC thresholds. In India, P&PI vaccines were also likely to be cost-effective at the lower HCOC threshold. While we found PSI vaccines to be less cost-effective than P&PI vaccines in general, a PSI vaccine priced at US$10 remained highly likely to be cost-effective at the 1-times GDP threshold in both India and China and at the upper HCOC threshold in India. In both countries, vaccination was projected to avert approximately 1 million RR/MDR-TB regimens by 2050.

We attributed the greater PSI vaccine impact on RR/MDR-TB in India than China, to the proportionally greater rate of re-infection and fast progression of latent RR/MDR-TB (which is avertible through post-infection vaccine efficacy) at baseline (Additional file 1, section 7.2). Moreover, in China, the RR/MDR-TB epidemic—driven by new infections among susceptible (naive) individuals—was more impacted by PRI than PSI vaccine efficacy (Additional file 1, section 7.2).

We found that vaccination averted a substantially higher absolute number of all TB cases and deaths in India than China. This reflected higher TB incidence and substantially higher TB mortality at baseline in India than China. The greater averted burden translated to greater averted life-years otherwise lost to TB; thus, despite lower TB management costs, for all vaccine profiles, ICERs in India were lower than in China. Correspondingly, savings in the TB programme were greater in India than China and reflect an underestimate for both countries, as vaccine-mediated protection and the dynamic impact of vaccination on the TB epidemic would continue to suppress TB burden beyond the model time horizon.

This study had several limitations pertaining to (1) model parameterisation and structure, (2) baseline scenarios and (3) vaccine implementation, which we consider in turn.

Data to substantiate natural history parameters for RR/MDR-TB were sparse and heterogeneous [28, 103, 104]. We assumed RR/MDR-TB transmissibility was equal to or lower than DS-TB [28, 29] and sampled from wide parameter priors. In the absence of evidence or a priori reasoning for differing values, we assumed other RR/MDR-TB and DS-TB parameters had the same values. As new empirical evidence arises, our predictions and estimates can be updated, but it is currently difficult to identify their direction of bias. Further, we maintained constant country-specific contact patterns over the model time horizon between DS and RR/MDR-TB. If individuals with RR/MDR-TB were to mix with one another preferentially, our results may overestimate vaccine impact.

To test our baseline assumptions of post-2018 constant case detection rates and drug-sensitivity testing coverage, we implemented a contrasting scaled up programmatic management scenario based on country-specific national policy. Our conclusions regarding relative vaccine impact remained robust to programmatic scale-up. However, we did not change RR/MDR-TB treatment success rate nor introduce a theoretical future highly effective diagnostic technology, either of which may reduce vaccine impact estimates. In India, we assumed the private health sector did not treat RR/MDR-TB successfully, based on in-country expert opinion. Should overall treatment success improve because of a larger private sector engagement effort, relative vaccine impact might remain stable, but absolute impacts may be reduced. We cannot speculate on the impact on cost-effectiveness, as this would depend on the specific mechanism of increased private sector engagement. Should the strategic focus of the Indian National Tuberculosis Programme change to include improved RR/MDR-TB treatment in the private healthcare sector, a new baseline scenario could be modelled to estimate the potential effect on vaccine impact. In China, we assumed the number of RR/MDR-TB treatment initiations contributing to programme costs was equal to the number starting treatment as reported by the Chinese Centre for Disease Control and Prevention (CDC). However, the averted number of treatments estimate applies to *all* RR/MDR-TB treatment—both CDC and non-CDC. As the total RR/MDR-TB treatment volume is unconstrained, our result of averted treatment may be an overestimate. We confined our health-economic analysis to a public sector healthcare perspective. TB-related costs to patients, including indirect costs from seeking healthcare and productivity losses, can be substantial [3]; these costs are not factored into our cost-effectiveness analysis. However, our analysis does include TB programme costs related to patient and private sector support: in India, we included nutritional support payments to TB patients and payments to incentivise private sector healthcare providers.

We implemented vaccine waning as an instantaneous loss of efficacy. If empirical data suggested a different waning shape, our estimates may over or underestimate the impact. We did not investigate vaccine targeting (e.g. by age, or by RR/MDR-TB risk group); targeting could improve cost-effectiveness estimates but reduce overall impact. We assumed population-wide mass campaigns were deployed instantaneously, with simultaneously applied costs, instead of through phased multi-year campaigns. These assumptions have one main consequence: vaccine-associated costs, which were calculated assuming that countries do not need additional capacity to deliver these campaigns, are an underestimate. However, as the benefits of such campaigns were also realised from the start, the ICERs may not be an underestimate. Finally, while we capture the cost-savings due to averted RR/MDR-TB treatment, the positive externalities this affords through a reduced contribution to antimicrobial resistance are not included in the cost-effectiveness analysis. We assumed a US$10 price per vaccine course and vaccine introduction in 2027. Should vaccine price decline over time, this might increase the probability of cost-effectiveness at the low HCOC threshold in either country or of PSI vaccines in China at the upper HCOC threshold. We speculate that delayed vaccine introduction by a few years would affect vaccine costs and benefits to similar extents and so is unlikely to substantially alter our findings.

Any evaluation of new TB vaccines must be compared to BCG. Neonatal BCG is still offered routinely in India and China [105]. Epidemiologic evidence suggests that BCG prevents severe disease—particularly miliary tuberculosis and tuberculous meningitis—in young children [106]. The effect of neonatal BCG vaccination on pulmonary tuberculosis risk in adults with estimates ranging from none to substantial [107–109]. Current evidence indicates that neonatal BCG vaccination will be inadequate to end transmission of M. tb among adults, which is a prerequisite for global TB elimination. In contrast, we suggest that adolescent and adult vaccination—with efficacy similar to late-stage vaccine candidate M72/AS01_E_—which prevents pulmonary TB disease may be a useful contributor towards this goal.

Previous estimates of TB vaccine cost-effectiveness either omit MDR-TB or do not model MDR-TB dynamically [8, 9]. This is the first study to dynamically model the impact of potential novel vaccines on MDR-TB. We modelled both the de novo acquisition of drug-resistance and transmission of drug-resistant *M. tuberculosis*. We also developed a country-specific cost model and estimated the cost-effectiveness of these vaccines. Consequently, our ICER estimates incorporated both the direct impact of vaccination on RR/MDR-TB and indirect effects due to reduced transmission. In India, we also adjusted for differential treatment by the private sector.

## Conclusions

Novel TB vaccination is likely to substantially reduce the future burden of RR/MDR-TB, while averting the need for RR/MDR-TB treatment. Vaccination may be cost-effective, but this depends on the local context and specific characteristics of the vaccine. There is an urgent need for new TB vaccines to prevent disease and for further investment in and acceleration of development of such vaccines to progress towards global TB elimination goals. As development of such vaccines continues, decision-makers should consider their potential role in national tuberculosis programmes and in wider antimicrobial resistance control efforts.

## Supplementary information

**Additional file 1: Technical Appendix**. **Figure S1** Model diagram. **S2** Case Detection and Treatment Success Rates. **S3** Demographic Model–India and China. **S4** Posterior distributions of sampled parameters—India. **S5** Posterior distributions of sampled parameters—India (contd). **S6** Posterior distributions of sampled parameters—China. **S7** Posterior distributions of sampled parameters—China (contd). **S8** Calibration Results—All Tuberculosis in India. **S9** Calibration Results—RR/MDR-TB in India. **S10** Baseline (no vaccine) projections–India. **S11** Calibration Results—Prevalence and Incidence of All TB in China. **S12** Calibration Results—Notifications and Mortality of All TB in China. **S13** Calibration Results—RR/MDR-TB in China. **S14** Baseline (no vaccine) projections–China. **S15** Latent tuberculosis infection. **S16** Incident tuberculosis, disaggregated by incidence source. **S17** Incidence Rate Reduction in India. **S18** Mortality Rate Reduction in India. **S19** Incidence Rate Reduction in China. **S20** Mortality Rate Reduction in China. **S21** RR/MDR-TB Cases Averted in India by 2030 and 2050. **S22** RR/MDR-TB Deaths Averted in India by 2030 and 2050. **S23** All TB Cases Averted in India by 2030 and 2050. **S24** All TB Deaths Averted in India by 2030 and 2050. **S25** RR/MDR-TB Cases Averted in China by 2030 and 2050. **S26** RR/MDR-TB Deaths Averted in China by 2030 and 2050. **S27** All TB Cases Averted in China by 2030 and 2050, **S28** All TB Deaths Averted in China by 2030 and 2050. **S29** RR/MDR-TB Treatment Regimens Averted by 2030 & 2050 in India. **S30** DS-TB Treatment Regimens Averted by 2030 & 2050 in India. **S31** RR/MDR-TB Treatment Regimens Averted by 2030 & 2050 in China. **S32** DS-TB Treatment Regimens Averted by 2030 & 2050 in China. **S33** ICER for vaccination in India at USD 10 per vaccine. **S34** ICER for vaccination in India at USD 30 per vaccine. **S35** ICER for vaccination in China at USD 10 per vaccine. **S36** ICER for vaccination in China at USD 30 per vaccine. **S37** ICER for vaccination in China at USD 10 per vaccine. **S38** ICER for vaccination in China at USD 30 per vaccine. **S39** ICER for vaccination in India at USD 10 per vaccine and 30% mass campaign coverage. **S40** ICER for vaccination in China at USD 10 per vaccine and 30% mass campaign coverage. **Tables S1** Model equations—symbols. **S2** Parameters for births and deaths. **S3** Parameters determining transmission and drug-resistance. **S4** Parameters determining disease progression. **S5** Parameters determining disease relapse and natural cure. **S6** Parameters related to treatment initiation and treatment success. **S7** Scale up of drug sensitivity testing coverage in the “Policy” scenario in China. **S8** RR/MDR-TB treatment regimens in the China “Policy” scenario. **S9** Calibration targets—India Rates are expressed per 100,000 population. **S10** Calibration targets—China. **S11** China Incidence Target Data. **S12** Modelled vaccine types and impact on disease states. **S13** TB-related Unit Costs. **S14** Vaccine-related Costs. **S15** Willingness to Pay Thresholds. **S16** Vaccine Impact by 50% efficacy, 10-year P&PI and PSI vaccines. **S17** Incidence of TB disaggregated by origin.

**Additional file 2.** Consolidated Health Economic Evaluation Reporting Standards (CHEERS) checklist.

## Data Availability

The datasets used and/or analysed during the current study are available from the corresponding author on reasonable request.
