# Correction to: The epidemiologic impact and cost-effectiveness of new tuberculosis vaccines on multidrug-resistant tuberculosis in India and China

**DOI:** 10.1186/s12916-022-02306-3

**Published:** 2022-03-01

**Authors:** Chathika K. Weerasuriya, Rebecca C. Harris, C. Finn McQuaid, Fiammetta Bozzani, Yunzhou Ruan, Renzhong Li, Tao Li, Kirankumar Rade, Raghuram Rao, Ann M. Ginsberg, Gabriela B. Gomez, Richard G. White

**Affiliations:** 1 TB Modelling Group, TB Centre and Centre for the Mathematical Modelling of Infectious Diseases, Department of Infectious Disease Epidemiology, Faculty of Epidemiology & Population Health, London School of Hygiene and Tropical Medicine, London, UK; 2 Currently employed at Sanofi Pasteur, Singapore, Singapore; 3 Department of Global Health and Development, Faculty of Public Health & Policy, London School of Hygiene and Tropical Medicine, London, UK; 4 Chinese Centre for Disease Control and Prevention, Beijing, China; 5 World Health Organisation, New Delhi, India; 6 National Tuberculosis Elimination Programme, New Delhi, India; 7 International AIDS Vaccine Initiative, New York, USA; 8 Current Affiliation: Bill and Melinda Gates Foundation, Washington, DC USA; 9 Currently employed at Sanofi Pasteur, Lyon, France

**Correction to: BMC Medicine 19, 1-13 (2021)**

**https://doi.org/10.1186/s12916-021-01932-7**

The original article contained errors in the Abstract which have all since been corrected.

