# Supplementary material for: The epidemiologic impact and cost-effectiveness of new tuberculosis vaccines on multidrug-resistant tuberculosis in India and China
Source: BMC Med. 2021 Feb 26;19:60. doi: 10.1186/s12916-021-01932-7 (PMC7908776; doi:10.1186/s12916-021-01932-7)
Supplement: Supplementary file 1 — Additional file 1: Technical Appendix. Figure S1 Model diagram. S2 Case Detection and Treatment Success Rates. S3 Demographic Model–India and China. S4 Posterior distributions of sampled parameters—India. S5 Posterior distributions of sampled parameters—India (contd). S6 Posterior distributions of sampled parameters—China. S7 Posterior distributions of sampled parameters—China (contd). S8 Calibration Results—All Tuberculosis in India. S9 Calibration Results—RR/MDR-TB in India. S10 Baseline (no vaccine) projections–India. S11 Calibration Results—Prevalence and Incidence of All TB in China. S12 Calibration Results—Notifications and Mortality of All TB in China. S13 Calibration Results—RR/MDR-TB in China. S14 Baseline (no vaccine) projections–China. S15 Latent tuberculosis infection. S16 Incident tuberculosis, disaggregated by incidence source. S17 Incidence Rate Reduction in India. S18 Mortality Rate Reduction in India. S19 Incidence Rate Reduction in China. S20 Mortality Rate Reduction in China. S21 RR/MDR-TB Cases Averted in India by 2030 and 2050. S22 RR/MDR-TB Deaths Averted in India by 2030 and 2050. S23 All TB Cases Averted in India by 2030 and 2050. S24 All TB Deaths Averted in India by 2030 and 2050. S25 RR/MDR-TB Cases Averted in China by 2030 and 2050. S26 RR/MDR-TB Deaths Averted in China by 2030 and 2050. S27 All TB Cases Averted in China by 2030 and 2050, S28 All TB Deaths Averted in China by 2030 and 2050. S29 RR/MDR-TB Treatment Regimens Averted by 2030 & 2050 in India. S30 DS-TB Treatment Regimens Averted by 2030 & 2050 in India. S31 RR/MDR-TB Treatment Regimens Averted by 2030 & 2050 in China. S32 DS-TB Treatment Regimens Averted by 2030 & 2050 in China. S33 ICER for vaccination in India at USD 10 per vaccine. S34 ICER for vaccination in India at USD 30 per vaccine. S35 ICER for vaccination in China at USD 10 per vaccine. S36 ICER for vaccination in China at USD 30 per vaccine. S37 ICER for vaccination in China at USD 10 per vaccine. S38 ICER for vacc [file 12916_2021_1932_MOESM1_ESM.pdf]

The epidemiologic impact and cost-effectiveness of new tuberculosis vaccines on multidrug resistant tuberculosis in China and India
Technical Appendix

Weerasuriya CK, Harris RC, McQuaid CF, Bozzani F, Ruan Y, Li R, Li T, Rade K, Rao R, Ginsberg A, Gomez GB, White RG

Contents

I Model Description 6

1 Summary 6

2 Model Structure and Parameterisation 7
2.1 Drug Sensitive Stratum 7
2.2 Drug Resistant Stratum 9
2.3 Equations 10
2.3.1 Susceptible Compartment 10
2.3.2 Drug-Sensitive Tuberculosis 10
2.3.3 Drug-Resistant Tuberculosis 12
2.3.4 Vaccine Stratum 14
2.4 Natural History Parameters 14
2.5 Diagnosis and Treatment Parameters 15
2.5.1 Private-Public Health Sector Treatment Proportions in India 15
2.5.2 Treatment Initiation 16
2.5.3 Treatment Regimens 17
2.6 Baseline Scenarios 17
2.7 Demographic Model 20
2.8 Transmission and Contact Mixing 24
2.8.1 India 24

3 Model Calibration 26
3.1 Sampling and Calibration Method 26
3.2 Calibration Targets 26
3.2.1 India 26
3.2.2 China 27

4 Vaccine implementation 30
4.1 Vaccine Characteristics 30
4.2 Deployment 31

5 Cost-effectiveness Analysis 32
5.1 TB-related Cost Model 32
5.2 Vaccine-related Cost Model 32
5.3 Disability Adjusted Life Years Calculations 34
5.4 Cost-effectiveness Analysis and Willingness to Pay Thresholds 34

II Furthers Results and Discussion 35

6 Calibration and Baseline Scenario Projections 35
6.1 Posterior Distributions of Parameters 35
6.2 India 40
6.2.1 Calibration 40
6.2.2 Baseline Scenario Projections 40
6.3 China 44
6.3.1 Calibration 44
6.3.2 Baseline Scenario Projections 44

7 Vaccine Impact **49**
7.1 Vaccine Efficacy and Duration of Protection . . . 49
7.2 Host-Infection Status Required for Vaccine Efficacy . . . 49
7.3 Scenario Analyses: Variable Mass Vaccine Campaign Coverage . . . 52
7.4 Scenario Analyses: Tuberculosis Incidence and Mortality Rate Reduction . . . 53
7.5 Scenario Analyses: Tuberculosis Cases and Deaths Averted . . . 58
7.6 Scenario Analyses: Averted Anti-tuberculosis Therapy . . . 67

8 Cost-effectiveness **72**
8.1 Incremental Cost Effectiveness Ratios . . . 72

9 Budget Impact **78**

List of Figures

S1 Model diagram . . . 8
S2 Case Detection and Treatment Success Rates . . . 19
S3 Demographic Model–India and China . . . 23
S4 Posterior distributions of sampled parameters—India . . . 36
S5 Posterior distributions of sampled parameters—India (contd.) . . . 37
S6 Posterior distributions of sampled parameters—China . . . 38
S7 Posterior distributions of sampled parameters—China (contd.) . . . 39
S8 Calibration Results—All Tuberculosis in India . . . 41
S9 Calibration Results—RR/MDR-TB in India . . . 42
S10 Baseline (no vaccine) projections–India . . . 43
S11 Calibration Results—Prevalence and Incidence of All TB in China . . . 45
S12 Calibration Results—Notifications and Mortality of All TB in China . . . 46
S13 Calibration Results—RR/MDR-TB in China . . . 47
S14 Baseline (no vaccine) projections–China . . . 48
S15 Latent tuberculosis infection . . . 50
S16 Incident tuberculosis, disaggregated by incidence source . . . 51
S17 Incidence Rate Reduction in India: 30% Mass Campaign Coverage . . . 52
S18 Incidence Rate Reduction in China: 30% Mass Campaign Coverage . . . 53
S19 Incidence Rate Reduction in India. . . . 54
S20 Mortality Rate Reduction in India. . . . 55
S21 Incidence Rate Reduction in China. . . . 56
S22 Mortality Rate Reduction in China. . . . 57
S23 RR/MDR-TB Cases Averted in India by 2030 and 2050. . . . 59
S24 RR/MDR-TB Deaths Averted in India by 2030 and 2050. . . . 60
S25 All TB Cases Averted in India by 2030 and 2050. . . . 61
S26 All TB Deaths Averted in India by 2030 and 2050. . . . 62
S27 RR/MDR-TB Cases Averted in China by 2030 and 2050. . . . 63
S28 RR/MDR-TB Deaths Averted in China by 2030 and 2050. . . . 64
S29 All TB Cases Averted in China by 2030 and 2050. . . . 65
S30 All TB Deaths Averted in China by 2030 and 2050. . . . 66
S31 RR/MDR-TB Treatment Regimens Averted by 2030 & 2050 in India. . . . 68
S32 DS-TB Treatment Regimens Averted by 2030 & 2050 in India. . . . 69
S33 RR/MDR-TB Treatment Regimens Averted by 2030 & 2050 in China. . . . 70
S34 DS-TB Treatment Regimens Averted by 2030 & 2050 in China. . . . 71
S35 ICER for vaccination in India at USD 10 per vaccine . . . 73
S36 ICER for vaccination in India at USD 30 per vaccine . . . 74
S37 ICER for vaccination in China at USD 10 per vaccine . . . 75
S38 ICER for vaccination in China at USD 30 per vaccine . . . 76
S39 ICER for vaccination in India at USD 10 per vaccine and 30% mass campaign coverage . . . 77
S40 ICER for vaccination in China at USD 10 per vaccine and 30% mass campaign coverage . . . 77

List of Tables

S1 Model equations—symbols . . . 11
S2 Parameters for births and deaths . . . 18
S3 Parameters determining transmission and drug-resistance . . . 20
S4 Parameters determining disease progression . . . 21
S5 Parameters determining disease relapse and natural cure . . . 21
S6 Parameters related to treatment initiation and treatment success . . . 22
S7 Scale up of drug sensitivity testing coverage in the "Policy" scenario in China . . . 22
S8 RR/MDR-TB treatment regimens in the China "Policy" scenario. . . . 23
S9 Calibration targets—India. Rates are expressed per 100,000 population . . . 28
S10 Calibration targets—China . . . 29
S11 China Incidence Target Data . . . 30
S12 Modelled vaccine types and impact on disease states . . . 31
S13 TB-related Unit Costs . . . 33
S14 Vaccine-related Costs . . . 33
S15 Willingness to Pay Thresholds . . . 34
S16 Vaccine Impact by 50% efficacy, 10-year P&PI and PSI vaccines . . . 50
S17 Incidence of TB disaggregated by origin . . . 50

Part I

Model Description

1 Summary

We took the following overarching steps during our analysis:

1. Constructed a dynamic transmission model of tuberculosis stratified by age, prior treatment history, drug resistance status and vaccina status. We only enabled the vaccine stratum during vaccine simulation.
2. Calibrated the model to nationally representative epidemiologic data from China and India.
3. Constructed future baseline scenarios of programmatic MDR-TB and TB management (i.e. without novel TB vaccines).
4. Implemented a country-specific cost-model for programmatic management of TB and RR/MDR-TB within the future baseline scenarios.
5. Implemented vaccines into the future baseline scenarios and estimate epidemiologic impact, vaccine cost-effectiveness, budget impact and treatment regimens averted by vaccination in comparison to baseline.

2 Model Structure and Parameterisation

We represented the following states of tuberculosis within the model:

1. Susceptible (never infected by Mycobacterium tuberculosis);
2. Latent infection (infected with Mycobacterium tuberculosis, but without active disease);
3. Infectious active disease (symptomatic bacteriologically positive disease capable of transmission);
4. Non-infectious active disease (symptomatic TB, but bacteriologically negative and incapable of transmission);
5. On-treatment for tuberculosis and;
6. Resolved (asymptomatic, having recovered from active tuberculosis, either via treatment or through natural cure).

A diagram of the model is presented in Figure S1.

Where appropriate, the natural history states were orthogonally stratified into three layers:

1. by treatment history, into never-treated and previously-treated status;
2. by drug-sensitive or drug-resistance status; and
3. by new TB vaccine vaccination status (the vaccinated stratum was only enabled when modelling vaccine scenarios).

Susceptible populations infected by either drug sensitive or drug resistant Mycobacterium tuberculosis could become latently infected or rapidly and directly to active disease. Populations in the active disease state had three possible exit routes: (1) detection and treatment; (2) natural cure; or (3) death from tuberculosis or other causes. Those who naturally cured moved to the resolved state. Those starting treatment entered the treatment state where they could either succeed treatment (and move to the resolved state) or fail (and move back to active disease). Treatment failures were redirected back to active TB disease. Latently infected and resolved populations could reactivate and relapse, respectively, back into the active disease state. These populations could also be reinfected and experience rapid progression to active disease but their rate of rapid progression to disease was lower than a naive individual—presumed due to pre-existing immunity—due to existing infection (latent) or previous experience of infection (resolved) .

We introduced an additional vaccine stratum, duplicating the drug resistance- and treatment history- strata within it, when simulating vaccine.

We modelled age in single years, over the range 0–99 years, and simulated over 1900–2050 in calendar time with a 3-month model timestep. Within this period, 1900–1999 represented "burn in" where the model equilibrated between various states. We calibrated to data select time points between 2000–2017, and projected from the model from 2018 to 2050. The full demographic model is described in section 2.7. We programmed the model in the R language for Statistical Computing [1] and implemented it as a series of difference equations.

2.1 Drug Sensitive Stratum

We modelled infection of the susceptible population at rate λ^S. Following infection, a proportion (p–"fast progressors") progressed directly to active disease, whereas the remainder (1-p) became latently infected. Those who progressed to active disease developed either *infectious* or *non-infectious* active disease through a partitioning parameter f. Latently infected populations could (1) remain latent; (2) reactivate to active disease at rate v or; (3) be reinfected at a reduced rate compared to the susceptible population (reduction specified by parameter x).

From the active disease compartments, populations could:

1. be detected and initiated on treatment, moving to a treatment compartment or;

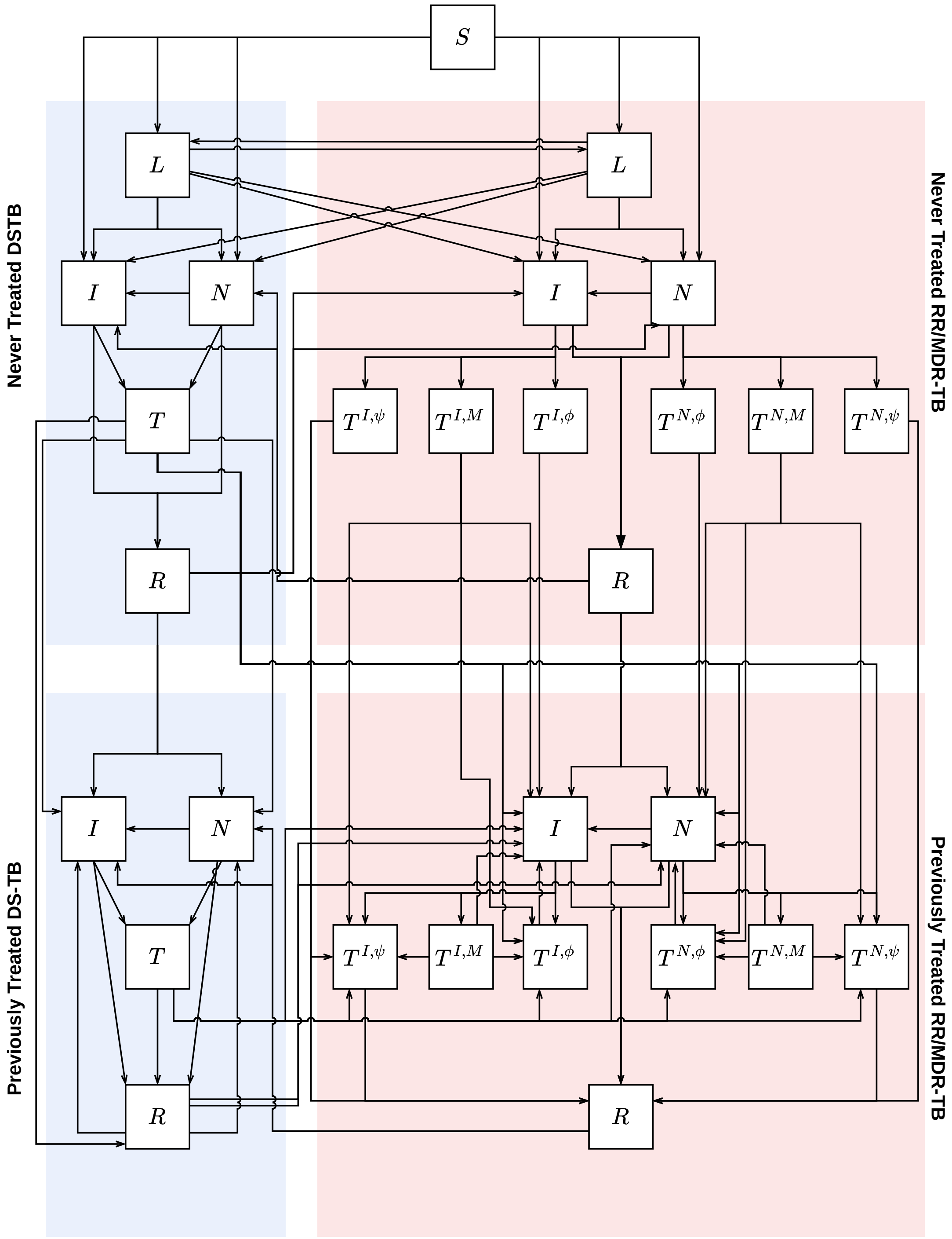

Figure S1: Model diagram. Compartments on blue background represent DS-TB. Compartments on red background represent RR/MDR-TB. Compartment symbols as follows: **S:** susceptible; **L:** latent; **I**: active infectious disease; **N:** active non-infectious disease; **T**: on-treatment, with ψ, ϕ, and M representing RR/MDR-TB treatments which succeed, fail and are misdiagnosed and treated with DS-TB treatment regimen, respectively; **R:** resolved.

2. undergo natural cure, moving to the resolved compartment or;
3. die due to tuberculosis.

Those initiated on treatment could:

1. be successfully treated and transition to the resolved compartment or;
2. experience treatment failure and transition back to the active disease compartment or
3. die on treatment

We used the WHO definition of prior treatment for tuberculosis ($\geq$ 2 months of previous anti-tuberculosis therapy). Consequently, populations in the *never-treated* drug-sensitive on-treatment compartment exited to destinations in the *previously-treated* stratum. Entry into the resolved state in the "never treated" stratum was only possible through natural cure. As was in latent infection, the resolved state conferred protection against reinfection compared to the susceptible population (through parameter x). Resolved populations could relapse back to active disease, but *could not* transition to the latently infected state.

Similarly, we only represented susceptible and latent states in the never-treated stratum. By definition, any populations with any previous experience of tuberculosis disease or treatment could not re-enter the susceptible state. Similarly, entry into the latent infection state was only possible from the susceptible state, or through re-infection while in the latent infection state itself. Relapse from the resolved state to latent infection was not possible in this model. Drug resistant populations in their respective susceptible, latent infection and resolved states could also be infected with drug sensitive tuberculosis as described above. Extant drug resistant latently infected and resolved states conferred also protection against drug sensitive reinfection. Populations experiencing treatment failure would transition back into active infectious disease, or active non-infectious disease in proportion to the relative prevalence of infectious vs non-infectious active disease in the preceding time step.

2.2 Drug Resistant Stratum

The drug resistant stratum differed from the drug sensitive in two key areas:

1. Acquisition of resistance
2. Treatment (stratification of outcomes and misdiagnosis)

A proportion of the population on treatment for drug-sensitive tuberculosis developed drug-resistance. We began the acquisition of drug resistance in 1970—timed to co-incide with the discovery of rifampicin [2–4]. We implemented this as a flow of a fixed proportion [5, 6] of the drug-sensitive on-treatment for tuberculosis population (in both treatment history strata) to the previously-treated drug-resistant stratum.

The population moving from the drug-sensitive to drug-resistant stratum in this way comprised multiple sub-populations. Firstly, a proportion was converted to a drug-resistant tuberculosis treatment regimen and moved into the previously-treated drug-resistant on-treatment compartments, through the *proportion correctly treated* parameter ($P_c(t)$—section 2.5). The remainder moved to previously-treated drug-resistant active disease (both infectious and non-infectious, determined by parameter f). Acquisition of resistance and transition to latent infection was impossible; latent infection is not represented in the previously treated stratum. Moreover, such a transition would represent a move from active disease on treatment to asymptomatic latent infection.

Treatment in the drug resistant stratum differed from drug sensitive stratum as follows. In the drug sensitive stratum, all treatment was aggregated into a single state from which treatment success, failure or death were possible outcomes. In comparison, in the drug resistant stratum, transition from active disease into treatment was disaggregated. Those with active disease could be empirically misdiagnosed and transition into a "misdiagnosed and treatment state", where they received inappropriate treatment for drug-sensitive tuberculosis. Alternatively, they could be correctly diagnosed and transition to treatment for drug resistant tuberculosis, either into treatment which is predestined to succeed, or into treatment which is predestined to fail. Correct identification and diagnosis of drug-resistant tuberculosis was governed by parameters determining

the *proportion receiving drug sensitivity testing* (for infectious active drug-resistant disease) and *proportion empirically diagnosed* (for both infectious and non-infectious drug-resistant disease), described in section 2.5. Treatment successes moved to the resolved compartment. Treatment failures returned to the previously-treated active disease state (infectious or non-infectious) from which they originated. In contrast to drug sensitive tuberculosis, transitions from drug-resistant infectious active disease remained separate to those with non-infectious active disease. The on-treatment predestined-to-fail state originating from active infectious disease continued to be infectious on treatment. This structure of treatment allowed for independent counting of time spent on treatment by both treatment failures and successes.

2.3 Equations

Model equations are described in sections 2.3.1, 2.3.22.3.3 and 2.3.4. A key to symbols in the equations is provided in Table S1.

2.3.1 Susceptible Compartment

Births in year k are added in the first timestep of each year to the susceptible compartment.

$$S_{t,0} = S_{t,0} + b_k$$

Mortality and new infections were then applied to the susceptible compartment.

$$S_{t,j} = (1 - \mu_{t-1,j} - \lambda^S_{t-1,j} - \lambda^R_{t-1,j})S_{t-1,j}$$

2.3.2 Drug-Sensitive Tuberculosis

Never-Treated Drug-Sensitive Tuberculosis

$$L^S_{t,j} = (1 - \mu_{t-1,j})L^S_{t-1,j} + (1 - p_j)\lambda^S_{t-1,j}(S_{t-1,j} + xL^R_{t-1,j}) + v_j L^S_{t-1,j} - x\lambda^S_{t-1,j} p L^S_{t-1,j} - x\lambda^R_{t-1,j} L^S_{t-1,j}$$

$$\begin{aligned} I^S_{t,j} = (1 - \mu_{t-1,j})I^S_{t-1,j} + \omega N^S_{t-1,j} + p_j f_j \lambda^S_{t-1,j}(S + xL^R_{t-1,j} + xL^S_{t-1,j}) \\ + v_j f_j L^S_{t-1,j} + (p_j x \lambda^S + {}_j r_j) f_j R^S_{t-1,j} \\ + p_j f_j x \lambda^S_{t-1,j} R^R_{t-1,j} - (n_j + \kappa^I_{t-1,j} + \mu_i) I^S_{t-1,j} \end{aligned}$$

$$\begin{aligned} N^S_{t,j} = \quad & (1 - \mu_{t-1,j})N^S_{t-1,j} + p_j(1 - f_j)\lambda^S_{t-1,j}(S + xL^R + xL^S) \\ & + v_j(1 - f_j)L^S_{t-1,j} + (1 - f_j)(p_j \lambda^S + r_j)R^S_{t-1,j} + (1 - f_j)p_j x \lambda^S_{t-1,j} R^R_{t-1,j} \\ & - (n_j + \kappa^N_{t-1,j} + \mu_n)N^S_{t-1,j} - \omega N^S_{t-1,j} \end{aligned}$$

$$T^S_{t,j} = (1 - \mu_{t-1,j})T^S_{t-1,j} + \kappa^I_{t-1,j} I^S_{t-1,j} + \kappa^N_{t-1,j} N^S_{t-1,j} - (\xi + \psi^S + \phi^S + \mu_T)T^S_{t-1,j}$$

$$R^S_{t-1,j} = (1 - \mu_{t-1,j})R^S_t + n_j(I^S_{t-1,j} + N^S_{t-1,j}) - (p_j x \lambda^S_{t-1,j} + r_j + p_j x \lambda^R_{t-1,j})R^S_{t-1,j}$$

Previously-Treated Drug-Sensitive Tuberculosis The equations which follow determine changes in the *previously*-treated drug sensitive compartments. Superscript pR and pS indicate previously-treated drug-resistant and drug-sensitive compartments, respectively.

Table S1: Model equations—symbols.

Type	Symbol	Description
Super/subscripts	Subscripts i	Time step
	Subscript j	Age
	Superscripts R, S, pR, pS	Applied to terms to indicate to resistant- or sensitive- ; p represents previously-treated.
	Superscripts I, N	Applied to terms relating to active infectious- or non-infectious TB, respectively
	Superscripts M, C	Applied to κ terms to indicate correct diagnosis and treatment initiation rate onto RR/MDR-TB treatment (C) or misdiagnosis and treatment initiation onto DS-TB treatment (M).
Compartments	S	Susceptible (naive to infection)
	L	Latently-infected
	T	On-treatment
	I	Active infectious disease
	N	Active non-infectioius disease
	R	Resolved following active disease, through treatment or natural cure
Coefficients	κ	Treatment initiation risks, derived from case detection ratio (as per section 2.5.2).
	ψ, ϕ	% Treatment failure and success, respectively
	λ	Transmission parameter
	$\mu_{i,j}, \mu_I, \mu_N, \mu_T$	Mortality risks: background, active infectious disease, active non-infectious disease and on-treatment respectively.
	ξ	Risk of acquiring multidrug resistance on first line therapy
	p_j	Proportion of (re-) infected Susceptible, Latents or Recovereds developing active TB, in age group j
	x	Protection from re-infection or developing active TB due to being latently infected or recovered from infection
	v_j	Risk of reactivation in age j of latently infected population
	f_j	Proportion of new active cases directly becom- ing infectious (primary disease), at age j
	ω	Risk of converting from non-infectious to infec- tious active case
	n_j	Risk of natural cure
	r_j	Risk of relapse from recovered to active (RR/MDR-)TB
	b_k	Number of births in year k
	$P_c(t)$	Proportion correctly diagnosed and initiated onto RR/MDR-TB treatment; see section 2.5.2 and eq 5.
	$Y_{t,j}$	Ratio of infectious- to non-infectious drug-sensitive disease in timestep t
	τ^M, τ^C	Exit risk, per time-step from drug-resistant TB treatment compartments, representing 24-months (τ^C) and 6-months (τ^M) of treatment.

$$I^{pS}_{t,j} = (1-\mu_{t-1,j})I^{pS}_{t-1,j} + \omega N^{pR}_{t-1,j} + (p_j x\lambda^S + {}_j r_j) f_j R^{pS}_{t-1,j} + p_j f_j x \lambda^S_{t-1,j} R^{pR}_{t-1,j}$$
$$+ Y_{t-1,j}\phi^S(T^S_{t-1,j} + T^{pS}_{t-1,j}) - (n_j + \kappa^I_{t-1,j} + \mu_i) I^{pS}_{t-1,j}$$

$$N^{pS}_{t,j} = (1-\mu_{t-1,j})N^{pS}_{t-1,j} + (1-f_j)(p_j\lambda^S + r_j)R^{pS}_{t-1,j} + (1-f_j)p_j x\lambda^S_{t-1,j}R^{pR}_{t-1,j} - (n_j + \kappa^N_{t-1,j} + \mu_n)N^{pS}_{t-1,j}$$

$$T^{pS}_{t,j} = (1-\mu_{t-1,j})T^{pS}_{t-1,j} + \kappa^I_{t-1,j} I^{pS}_{t-1,j} + \kappa^N_{t-1,j} N^{pS}_{t-1,j} - (\xi + \psi^S + \phi^S + \mu_T)T^S_{t-1,j}$$

$$R^{pS}_{t-1,j} = (1-\mu_{t-1,j})R^{pS}_t + n_j(I^{pS}_{t-1,j} + N^{pS}_{t-1,j}) + \psi^S(T^S_{t-1,j} + T^{pS}_{t-1,j}) - (p_j x\lambda^S_{t-1,j} + r_j + x\lambda^R_{t-1,j})R^{pS}_{t-1,j}$$

2.3.3 Drug-Resistant Tuberculosis

Never-Treated Drug-Resistant Tuberculosis The equations which follow determine changes in the never treated drug resistant compartments.

Here, superscript R and S indicates never-treated drug-resistant and drug-sensitive compartments respectively.

$$L^R_{t-1,j} = (1-\mu_{t-1,j})L^R_{t,j} + (1-p_j)\lambda^R_{t-1,j}(S_{t-1,j} + xL^S_{t-1,j}) + v_j L^R_{t-1,j} - x\lambda^R_{t-1,j} p L^R_{t-1,j} - x\lambda^S_{t-1,j} L^R_{t-1,j}$$

$$I^R_{t,j} = (1-\mu_{t-1,j})I^R_{t-1,j} + \omega N^R_{t-1,j} + p_j f_j \lambda^R_{t-1,j}(S + xL^R_{t-1,j} + xL^S_{t-1,j}) + v_j f_j L^R_{t-1,j}$$
$$+ (p_j x\lambda^R + r_j) f_j R^R_{t-1,j} + p_j f_j x\lambda^R_{t-1,j} R^S_{t-1,j} - (n_j + \kappa_{t-1,j} + \mu_i) I^S_{t-1,j}$$

$$N^R_{t,j} = (1-\mu_{t-1,j})N^R_{t-1,j} + p_j(1-f_j)\lambda^R_{t-1,j}(S + xL^R + xL^S)$$
$$+ v_j(1-f_j)L^R_{t-1,j} + (1-f_j)(p_j x\lambda^R_{t-1,j} + r_j)R^R_{t-1,j} + (1-f_j)p_j x\lambda^R_{t-1,j}$$
$$R^S_{t-1,j} - (n_j + \kappa^N_{t-1,j} + \mu_n)N^R_{t-1,j} - \omega N^R_{t-1,j}$$

$$T^{I\psi}_{t,j} = (1-\mu_{t-1,j})T^{I\psi}_{t-1,j} + \kappa^{C,I}\psi^R I^R_{t-1,j} - (\tau^C + \mu)T^{I\psi}_{t-1,j}$$

$$T^{I\phi}_{t,j} = (1-\mu_{t-i,j})T^{I\phi}_{t-1,j} + \kappa^{C,I}\phi^R I^R_{t-1,j} - (\tau^C + \mu)T^{I\psi}_{t-1,j}$$

$$T^{N\psi}_{t,j} = (1-\mu_{t-1,j})T^{N\psi}_{t-1,j} + \kappa^{C,N}\psi^R I^R_{t-1,j} - (\tau^C + \mu)T^{N\psi}_{t-1,j}$$

$$T^{N\phi}_{t,j} = (1-\mu_{t-i,j})T^{N\phi}_{t-1,j} + \kappa^{C,N}\phi^R I^R_{t-1,j} - (\tau^C + \mu)T^{N\psi}_{t-1,j}$$

$$T^{M,I}_{t,j} = (1-\mu_{t-i,j})T^{M,I}_{t-1,j} + \kappa^{M,I} I^R_{t-1,j} - (\tau^M + \mu_m)T^{M,I}_{t-1,j}$$

$$T^{N,I}_{t,j} = (1-\mu_{t-i,j})T^{N,I}_{t-1,j} + \kappa^{M,N} I^{R}_{t-1,j} - (\tau^{M} + \mu_{m})T^{N,I}_{t-1,j}$$

$$R^{R}_{t,j} = (1-\mu_{t-1,j})R^{R}_{t-1,j} + n_j(I^{R}_{t-1,j} + N^{R}_{t-1,j}) - (\lambda^{R}_{t-1,j}p_j x + r_j + \lambda^{S}_{t-1,j}p_j x)R^{R}_{t-1,j}$$

Previously-Treated Drug-Resistant Tuberculosis The equations which follow determine changes in the *previously*-treated drug sensitive compartments. Superscript pR and pS indicate previously-treated drug-resistant and drug-sensitive compartments, respectively.

$$\begin{aligned} I^{pR}_{t,j} = (1-\mu_{t-1,j})I^{pR}_{t-1,j} + \omega N^{pR}_{t-1,j} + (x\lambda^{R} + r_j)f_j R^{pR}_{t-1,j} + f_j x \lambda^{R}_{t-1,j}R^{pS}_{t-1,j} \\ + \xi f_j (1-P_c(t))(T^{S}_{t-1,j} + T^{pS}_{t-1,j}) + \tau^{C}(T^{I\phi}_{t-1,j} + T^{pI\phi}_{t-1,j}) \\ + \tau^{M}(1-P_c(t))(T^{M,I}_{t-1,j} + T^{pM,I}_{t-1,j}) - (n_j + \kappa^{I}_{t-1,j} + \mu_{T})I^{S}_{t-1,j} \end{aligned}$$

$$\begin{aligned} N^{pR}_{t-1,j} = (1-\mu_{t-1,j})N^{pR}_{t-1,j} + (1-f_j)(x\lambda^{R}_{t-1,j} + r_j)R^{pR}_{t-1,j} + (1-f_j)x\lambda^{R}_{t-1,j}R^{pS}_{t-1,j} \\ + \xi(1-f_j)(1-P_c(t))(T^{S}_{t-1,j} + T^{pS}_{t-1,j}) + \tau^{C}(T^{N\phi}_{t-1,j} + T^{pN\phi}_{t-1,j}) \\ + \tau^{M}(1-P_c(t))(T^{M,N}_{t-1,j} + T^{pM,N}_{t-1,j}) - \omega N^{pR}_{t-1,j} - (n_j + \kappa^{N}_{t-1,j} + \mu_n)N^{R}_{t-1,j} \end{aligned}$$

$$\begin{aligned} T^{pI\psi}_{t,j} = (1-\mu_{t-1,j})T^{I\psi}_{t-1,j} + \kappa^{C,I}\psi^{R} I^{pR}_{t-1,j} + \psi^{R}P_c(t)\tau^{M}(T^{M,I}_{t-1,j} + T^{pM,I}_{t-1,j}) \\ + f_j P_c(t)\psi^{R}\xi(T^{S}_{t-1,j} + T^{pS}_{t-1,j}) - (\tau^{C} + \mu_{T})T^{I\psi}_{t-1,j} \end{aligned}$$

$$\begin{aligned} T^{pI\phi}_{t,j} = (1-\mu_{t-1,j})T^{I\phi}_{t-1,j} + \kappa^{C,IR}\phi^{R} I^{pR}_{t-1,j} + \phi^{R}P_c(t)\tau^{M}(T^{M,I}_{t-1,j} + T^{pM,I}_{t-1,j}) \\ + f_j P_c(t)\phi^{R}\xi(T^{S}_{t-1,j} + T^{pS}_{t-1,j}) - (\tau^{C} + \mu_{I})T^{I\psi}_{t-1,j} \end{aligned}$$

$$\begin{aligned} T^{pN\psi}_{t,j} = (1-\mu_{t-1,j})T^{N\psi}_{t-1,j} + \kappa^{C,N}\psi^{R} N^{pR}_{t-1,j} + \psi^{R}P_c(t)\tau^{M}(T^{M,N}_{t-1,j} + T^{pM,N}_{t-1,j}) \\ + (1-f_j)P_c(t)\psi^{R}\xi(T^{S}_{t-1,j} + T^{pS}_{t-1,j}) - (\tau^{C} + \mu_{T})T^{N\psi}_{t-1,j} \end{aligned}$$

$$\begin{aligned} T^{pN\phi}_{t,j} = (1-\mu_{t-1,j})T^{N\phi}_{t-1,j} + \kappa^{C,N}\phi^{R} N^{pR}_{t-1,j} + \phi^{R}P_c(t)\tau^{M}(T^{M,N}_{t-1,j} + T^{pM,N}_{t-1,j}) \\ + (1-f_j)P_c(t)\phi^{R}\xi(T^{S}_{t-1,j} + T^{pS}_{t-1,j}) - (\tau^{C} + \mu_{N})T^{N\phi}_{t-1,j} \end{aligned}$$

$$T^{M,I}_{t,j} = (1-\mu_{t-1,j})T^{M,I}_{t,j} + \kappa^{M,I}_{t-1,j} I^{pR}_{t-1,j} - (\tau^{M} + \mu_{I})T^{M,I}_{t-1,j}$$

$$T^{M,N}_{t,j} = (1-\mu_{t-1,j})T^{M,N}_{t,j} + \kappa^{M,N}_{t-1,j} I^{pR}_{t-1,j} - (\tau^{M} + \mu_{N})T^{M,N}_{t-1,j}$$

$$R^{pR}_{t,j} = (1-\mu_{t-1,j})R^{pR}_{t-1,j} + n_j(I^{pR}_{t-1,j} + N^{pR}_{t-1,j})$$
$$+ \tau^C(T^{pI\psi}_{t-1,j} + T^{pN\psi}_{t-1,j} + T^{I\psi}_{t-1,j} + T^{N\psi}_{t-1,j}) - (p_j x \lambda^S_{t-1,j} + r_j + \lambda^R_{t-1,j} x)R^{pR}_{t-1,j}$$

Treatment Initiation Rate Terms Per section 2.5.2 and equation 5, we (1) partitioned overall treatment initiation, κ for RR/MDR-TB into initiation of correct vs incorrect (misdiagnosis) therapy; (2) included probabilities of empirical detection vs drug-sensitivity testing; into four sub-components (equation 1). These were the specific treatmment initiation rates onto correct treatment for active infectious RR/MDR-TB; correct treatment for active non-infectious RR/MDR-TB; incorrect (misdiagnosed) treatment for active infectious RR/MDR-TB; and incorrect (misdiagnosed) treatment for active non-infectious RR/MDR/TB. Treatment inititiation onto correct treatment was then partitioned into those predestinated to succeed and fail treatment, as described above.

$$\begin{aligned}
\kappa^{C,I}_{t,j} &= \kappa^{I}_{t,j} P_c(t) \\
\kappa^{C,N}_{t,j} &= \kappa^{N}_{t,j} P_c(t) \\
\kappa^{M,I}_{t,j} &= \kappa^{I}_{t,j} (1-P_c(t)) \\
\kappa^{M,N}_{t,j} &= \kappa^{N}_{t,j} (1-P_c(t))
\end{aligned} \tag{1}$$

2.3.4 Vaccine Stratum

We duplicated all compartments, where for a given compartment C, we created a corresponding compartment C_V. The equations determining flow between the C_V compartments were identical to those for C, with coefficients for v, r and p modified by vaccine efficacy, as described in section 4. Within the time step of immunisation, vaccination and waning of protection were implemented as flow from $C \rightarrow C_V$ and $C_V \rightarrow C$ respectively, per equation 2,

$$\begin{aligned}
C_V &= C_V + qC - (1-q)wC_V \\
C &= C - qC + (1-q)wC_V
\end{aligned} \tag{2}$$

where q represents the coverage of vaccination and w represents the proportion experiencing waning of protection in that time step.

2.4 Natural History Parameters

Natural history parameters with prior ranges and references are presented in Tables S2–S5.

The natural history of tuberculosis varies with age, which manifests as differences in presentation (including extra-pulmonary, pulmonary or disseminated disease), rates of progression following infection, reactivation from latency, relapse following natural cure or treatment and tuberculosis related mortality [7–10]. Therefore, we modelled the corresponding natural history parameters p (progression to active disease), f (progression to infectious disease), v (reactivation from latency), r (relapse from cure or after treatment), μ_I and μ_N (infectious and non-infectious TB mortality) as age-variant. We independently sampled age-specific values for children (age<15), and adults (age $\geq$ 15) for these parameters in the India model. We also sampled additional age-specific values for elderly (age $\geq$ 65) in the China model, including an elderly-specific risk of natural cure (n), as data were available to calibrate this model to additional targets for elderly age groups.

Age-specific parameter ranges were based on data where available (Tables S2–S5). No direct data was found to inform the upper bound of the parameter p (progression to active disease) in the elderly age-group. Therefore, we assumed that immunocompromise served as a reasonable approximation of age-related decline in immunity and used data from HIV+ populations to inform this value, consistent with historical literature [11, 12]. Point estimates of overall, all-age TB mortality risk were informed by systematic reviews of data

from the pre-chemotherapeutic era [13, 14]. Based on empirical data [7], we applied age-specific TB mortality calibration factors ($uiscale$ terms) to these point estimates (represented by TB mortality parameters μ_I and μ_T), to generate age-specific TB mortality risks used within the model (Table S2). To allow for higher rates of progression, infectiousness and reactivation from latency in the elderly, and higher TB mortality in children and elderly, than adults, we constrained the sampling process (section 3.1) to retain parameter values only if the elderly/child values were greater than adult values [7–10].

Values for z (risk of transmission per infectious contact), x (protection against reinfection conferred by latent infection or resolved disease), ω (risk of converting from non-infectious to infectious disease), ξ (risk of developing drug-resistance on first line therapy) were fixed across ages.

We calibrated force of infection by multiplying *Mycobacterium tuberculosis* transmission parameters ($\lambda^S_{i,j}$ and $\lambda^R_{i,j}$) by calibration factors q^S and q^R for DS-TB and RR/MDR-TB, respectively. Estimates of the fitness cost of multidrug resistance and its consequent impact on relative transmissibility are heterogeneous [15–17]. In the absence of a precise estimate, we assumed (1) that drug-resistance was unlikely to confer a transmissibility benefit; and (2) a wide prior range of fitness, constrained to be lower than or equal to DS-TB and derived q^R by multiplying q^S with sampled fitness parameter DR_TS (Table S3). We derived an estimate of the risk of acquiring drug-resistance on DS-TB treatment from systematic reviews of the impact of rifampicin duration on TB treatment outcomes [5, 6, 18].

2.5 Diagnosis and Treatment Parameters

We used case detection ratios (CDR) from WHO [19] to inform treatment initiation from active infectious disease (compartments I and N). We fitted a generalised logistic function to CDR data from China and India [19] to remove artefactual noise in the data and derive a smoothed curve over 2000–2017 (Figure S2A). We then converted CDR to a risk of treatment initiation from prevalent active disease (section 2.5.2). We applied the same case detection ratio to both DS-TB and RR/MDR-TB tuberculosis, as we modelled the identification of drug-resistance as an event subsequent to diagnosis (section 2.5.2). To fit the model to case-notification and incidence data, we applied a scaling factor ($cdrscale$—Table S6) prior to converting to a treatment initiation risk. Bacteriologically negative (non-infectious) tuberculosis was assumed to be detected at a lower rate relative to bacteriologically positive TB [11], using a sampled parameter e.

We independently sampled values of natural cure, mortality and case detection scaling factors during the calibration process (section 3.1) and discarded sample sets where the resulting scaled sum of treatment initiation, mortality and natural cure (the total outflow from active disease) exceeded 1. When simulating increased future case detection (and therefore treatment initiation) in the India "Policy" scenario (section 2.6), we scaled this total outflow to equal 1 as necessary, while maintaining the relative proportions of treatment initiation, natural cure and mortality.

Parameter prior ranges, constraints and details are summarised in Table S6.

2.5.1 Private-Public Health Sector Treatment Proportions in India

In India, the CDR accounted for differential detection the public and private sectors. We adjusted the case detection ratio for the presence the private sector, which manages approximately 40–66% of all tuberculosis treatment [20, 21]. Despite this only 20% of tuberculosis case notifications were estimated to originate from private sector providers in 2017 [22, 23]. Moreover, the quality of care and treatment outcomes in the private sector differ to that of the public sector [22].

We accounted for the presence of the private sector in India by incorporating (1) the proportion TB treatment in the private sector, ppm; and (2) the proportion of all case notifications originating from the private sector. We increased the proportion of case notifications arising from the private sector from 0% in 2012 to 20% in 2017 per WHO data [22] and adjusted the overall case detection ratio as per,

$$
\begin{aligned}
R &= \frac{T_{\text{Pu}} + T_{\text{Pr}}}{I} \\
T_{\text{Pr}} &= ppm(T_{\text{Pu}} + T_{\text{Pr}}) \\
T_{\text{Pu}} &= \frac{C \cdot I \cdot N_{\text{Pu}}}{P_{\text{Pu}}} \\
R &= C \cdot \frac{N_{\text{Pu}}}{P_{\text{Pu}}} \left(1 + \frac{ppm}{1 - ppm}\right)
\end{aligned}
\tag{3}
$$

where R is adjusted case detection ratio, assuming each notification reflects a treatment initiation, I is incidence, C is case detection ratio, N_{Pu} is the the proportion, among notifications, which originate in the public sector, P_{Pu} is the proportion of treatment initiations in the public sector which are notified[1], ppm is the proportion of all treatment which occurs in the *private sector*, T_{Pr} is the total treatment volume in the private sector and T_{Pu} is the total treatment volume in the public sector.

The upper bound for the ppm prior range was informed by literature, which suggests up to 66% of treatment might occur in the private sector [21]. We derived the lower bound compatible with WHO notification data through back-calculation [22]. WHO estimates that percentage of *notifications* that originate from the private sector climbs from 0% 2012 to 20% in 2017, which in the "Policy" baseline scenario we further increase to 35% by 2025. We combined this range 0–35% with reported case detection ratio data for India until 2018 [19] (and an assumed increase in case detection ratio to 85% by 2025 in the "Policy" scenario—section 2.6) to estimate a minimum bound of 37% using equation 3. Here, we assumed (1) that 100% of public sector treatment initiations are notified; (2) the proportion of private sector treatment initiations *which are* notified cannot exceed 100% of all private sector treatment initiations; and (3) overall risk of treatment initiation cannot exceed 100%.

2.5.2 Treatment Initiation

WHO defines case detection as the ratio of case notifications among estimated *incident* cases. Assuming that each case notification corresponded to an initiation of anti-tuberculosis treatment, we modelled the outflow from prevalent, active, disease as the sum of treatment, natural cure and mortality. Based on this assumption, we derived a risk of treatment initiation per equation 4.

$$
\begin{aligned}
\text{CDR} &\approx \frac{\kappa}{\kappa + \mu + n} \\
\kappa &\approx \frac{\text{CDR}(\mu + n)}{1 - \text{CDR}}
\end{aligned}
\tag{4}
$$

Here, κ is risk of treatment initiation; CDR is case detection ratio; μ is mortality risk; and n is risk of natural cure.

In the drug sensitive stratum, following treatment initiation we moved populations from the active disease compartments into the on-treatment compartment, assuming that all such cases receive correct anti-tuberculosis therapy.

In contrast, although we *initiated* drug-resistant active disease cases onto treatment at the same rate as drug-sensitive active disease, they subsequently moved to one of three on-treatment destinations: successful RR/MDR-TB treatment, failing RR/MDR-TB treatment and failing (inappropriate) DS-TB treatment. We first partitioned the treatment initiation outflow into two streams—correct treatment (onto RR/MDR-TB treatment, irrespective of treatment outcome) or incorrect treatment (onto DS-TB treatment)—using the *proportion correctly treated*:

$$
P_c(t) = P_{dst}(t) + P_e(t)(1 - P_{dst}(t)) \tag{5}
$$

[1] $P_{\text{Pu}}$was assumed to equal 100% when calculating the adjusted case detection ratio, but uncertainty was introduced by adding uncertainty intervals to notification rate calibration targets (section 3.2)

where $P_c(t)$ is proportion correctly treated at time t; $P_{dst}(t)$ is proportion receiving drug sensitivity testing at time t; and $P_e(t)$ is proportion empirically identified as drug-resistant at time t.

To construct functions $P_{dst}(t)$ and $P_e(t)$, we linearly interpolated from zero (in 2007 and 1970) to values *dst_p* in 2018 and *emp_tx_p* in 2018, respectively. *dst_p* was the proportion receving a drug sensitivity test in 2018 and *emp_tx_p* was the proportion empirically identified as drug-resistant in 2018. A study on scale up of programmatic MDR-TB management in China [24] and results from the first national tuberculosis anti-tuberculosis drug resistance survey of India [25] indicate that programmatic drug-sensitive testing was initiated in 2006–2007; therefore, we initialised $P_{dst}(t)$ at zero in 2007 for both China and India. DST coverage was set at 35% in 2018 based on data from the national strategic plan for tuberculosis elimination in India [26] and expert opinion from both China and India. Beyond 2018, $P_{dst}(t)$ remained constant ("Status Quo" scenario) or incremented ("Policy" baseline scenario) as described in section 2.6. We assumed that bacteriologically-negative patients did not receive drug-sensitivity testing; therefore for identification of non-infectious (bacteriologically-negative) RR/MDR-TB, we set $P_{dst}(t)$ equal to 0. We sampled *emp_tx_p* during calibration (section 3.1).

Once partioned, we further stratified the "correct treatment" flow using the RR/MDR-TB treatment success rate (see section 2.5.3) into successful and failing RR/MDR-TB treatment respectively.

RR/MDR-TB treatment in China

Based on 2013 drug resistance survey results, WHO estimates approximately 46–69,000 cases of RR-TB among all notified pulmonary TB disease in China [19]. However, between 2014–2017, the global TB database reports only 5,807–13,069 laboratory confirmed cases of RR/MDR-TB diagnosed and treated in China. We modelled this gap as treatment occurring outside the of Chinese Centre for Disease Control and Prevention (CDC) tuberculosis dispensary system [27]. We sampled a parameter chr, which we used to partition the total RR/MDR-TB treatment initiations in the model; the chr treatment subflow was calibrated to total RR/MDR-TB volume in the China CDC system, which used towards cost-effectiveness calculations (sections 3.1 and 5.4).

2.5.3 Treatment Regimens

Per WHO guidelines [46, 47], we assumed a treatment duration of 6 months for DS-TB treatment and 24-months for RR/MDR-TB treatment (the latter up to 2018—section 2.6 for differences between baseline scenarios).

We used DS-TB treatment success rates for India and China per the WHO TB database [19] up to 2018 (Figure S2B), which we held constant when projecting into the future in all scenarios. For India, we assumed WHO values to applied to treatment in the public sector; we assumed treatment success rate in the private sector to be 5% lower at any given time point and calculated an overall treatment success rate using the private-public mix parameter ppm (sections 2.5.1 and 3.1).

For second line therapy we assumed a constant treatment success rate of 46% and 48% in China and India respectively [48, 49] from 1970 over the model time horizon.

2.6 Baseline Scenarios

We assumed a baseline scenario of unchanged future programmatic (i.e. non-vaccine) management of DS-TB and RR/MDR-TB as constant case detection, drug-sensitivity testing and TB treatment after 2018. In this document, this is referred to as the "Status Quo" scenario. To test the effect of these assumptions, we performed a scenario analysis by simulating vaccine in an alternative "Policy scenario" which incorporated country-specific changes to programmatic TB management.

India

Per the National Strategic Plan for Tuberculosis Elimination 2017-25 [26] we implemented the following in the India "Policy" scenario:

1. Linearly increase overall case detection ratio (across both private and public sectors) to 85% by 2025;

Table S2: Parameters for births and deaths

Symbol	Description	Prior Range and Constraints	References
**Births**			
b_k	Number of births in year k	Source data per UN ESA	[28]
**Deaths**			
$\mu_{i,j}$	Background (all cause) risk of deaths	Calculated from UN DESA Population Division mortality projections as number of projected deaths in a given age, divided by the total projected population in that age group j, in year i	[28]
μ_I<br>μ_N	Death risk for infectious untreated TB, varies by age<br>Death risk for non-infectious untreated TB, varies by age	0.6<br>0.21<br>Both values calibrated by $uiscale$ to TB mortality, as below	[14]
μ_T	Death risk on-treatment for DS-TB, varies by age	0.035<br>Value calibrated by $uiscale$ to TB mortality, as below<br>The definition of treatment failure in RR/MDR-TB include mortality; therefore, a specific mortality term is not applied while "on-treatment" for RR/MDR-TB. Instead, mortality terms for infectious and non-infectious RR/MDR-TB are applied as appropriate.	[13]
$uiscaleA$<br>$uiscaleC$<br>$uiscaleE$<br>(China only)	Calibration factor for TB mortality	uiscaleC = uiscale[j < 15] = (-)0.99 – (+)0.99<br>uiscaleA = uiscale[j ≥15] = (-)0.99 – (+)0.99 (India)<br>uiscaleA = uiscale[j ≥ 15, <65] = (-)0.99 – (+)0.99 (China)<br>uiscaleE = uiscale[j ≥ 65] = (-)0.99 – (+)0.99 (China)<br>Constraint: Parameter set only retained if child (India and China) and elderly (China only) parameters selected were greater than or equal to adult parameter.	Assumed

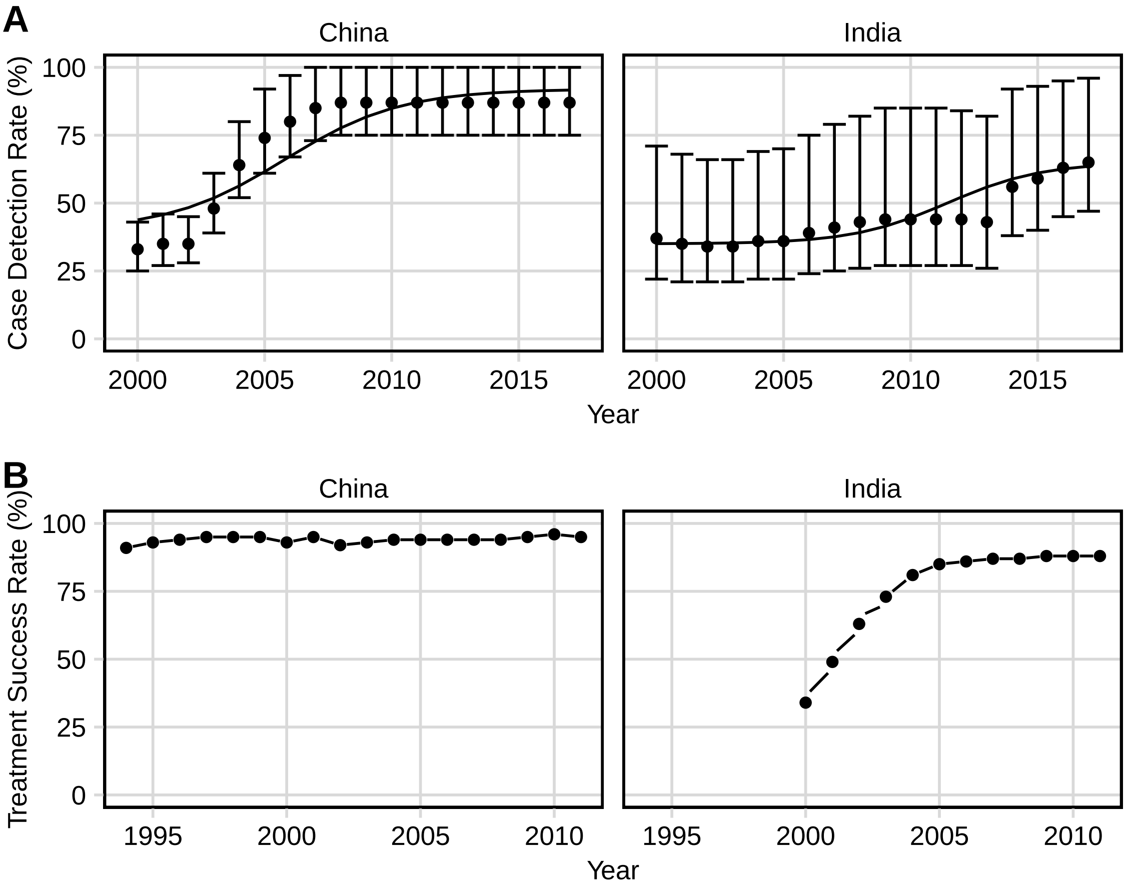

Figure S2: Case Detection and Treatment Success Rates. **A**: case detection ratios for China and India. Dots represent WHO estimates, solid line represents fitted curve. **B:** Treatment Success Rate for first line therapy. Source for A and B: WHO Tuberculosis Database [19].

Table S3: Parameters determining transmission and drug-resistance

Symbol	Description	Prior Range and Constraints	References
**Transmission**			
$\lambda^S_{i,j}, \lambda^R_{i,j}$	M. tb transmission risk (force of infection) in time step i, for age j for DS-TB and RR/MDR-TB	Calculated in model (equations 6 and 7)	
q^S	Force of infection calibration factor—DS-TB	Scales respiratory contacts to annual number of contacts and calibrates to TB incidence<br>Calibration range: 0–1 (China) and 0–5 (India)	Assumed
q^R	Force of infection calibration factor—RR/MDR-TB	Calculated as:<br>$q^R = q^S \times \text{DR\_TS}$	Assumed
$D_{m,y}$	Daily number of respiratory contacts by age group m and contacts in age group y	Calibrated by q^S to match TB incidence	[29, 30]
z	Probability of transmission per respiratory contact between an Infectious and Susceptible individual	Fixed: 0.1	[12, 31, 32]
**Drug Resistance**			
ξ	Risk of acquiring multidrug resistance on first line therapy	0.003–0.012	[5, 6, 18]
DR_TS	Relative transmission fitness of RR/MDR-TB	0–1	Assumed

2. Increase in drug sensitivity testing coverage among public sector notifications from 35% in 2018 [2] to 100% in 2025;

3. Increase in proportion of notifications originating in the private sector to 35% by 2025.

We assumed that all RR/MDR-TB treatment in the private sector was unsuccessful [*Rao, R., National Tuberculosis Elimination Programme, personal communication*].

China

In the “Policy” scenario for the China case study, we implemented the following programmatic management of MDR-TB:

1. Scale up of DST coverage (Table S7).

2. Change in RR/MDR-TB treatment regimen from a sole 24-month regimen to a mixture of 3 regimens, two of length 24 months and one of length 9 months, with no change in treatment efficacy, but with differing regimen costs (Table S8).

2.7 Demographic Model

We populated the underlying demographic model with new births per year and all-cause, age-wise mortality with data published by the United Nations Department of Economic and Social Affairs (UN ESA), Population Division [28].

We input the absolute number of births per annum into the first time step of the year. Age-wise mortality data was available in 5-year age- and calendar-year blocks; this is converted to annual, single-year age-wise

[2] The NSP 2017-2025 reports the proportion of notified pulmonary TB patients receiving a drug-sensitivity test in 2016 as 30% [26].
[3] 6LFX(MFX)-BQD-LZD-CFZ-CS/14MFX-CFZ-CS
[4] 6CmLfx(Mfx)PtoCsZ/18LfxPtoCsZ

Table S4: Parameters determining disease progression following infection

Symbol	Description	Prior Range and Constraints	References
p_j	Proportion of (re-) infected Susceptible, Latents or Recovereds developing active TB, in age group j	p[j < 15] = 0.01–0.06<br>p[j ≥ 15] = 0.08–0.2 (India)<br>p[j ≥ 15, <65] = 0.08–0.2 (China)<br>p[j ≥ 65] = 0.08–0.36 (adult and HIV-positive range)<br>Constraints: In China, the parameter set was retained only if elderly parameter selected was greater than or equal to adult parameter.	[12, 31–33]
x	Protection from re-infection or developing active TB due to being latently infected or recovered from infection	(1-x) = value for the level of protection afforded<br>Range: 0.25–0.41	[31, 32, 34, 35]
v_j	Risk of reactivation in age j of latently infected population	v[j<15] = 0.0001–0.0003<br>v[j ≥15] = 0.0001–0.0003 (India)<br>v[j ≥15, j<65] = 0.0001–0.0003 (China)<br>v[j ≥ 65] = 0.0001–0.04 (elderly; China)<br>Constraint: In China, parameter set only retained if elderly parameter selected was greater than or equal to adult parameter.	[7, 12, 31, 35, 36]
f_j	Proportion of new active cases directly becom- ing infectious (primary disease), at age j	f[j < 15] = 0-0.15<br>f[j ≥ 15] = 0.25-0.75 (India)<br>f[j ≥ 15, <65] = 0.25-0.75 (China)<br>f[j ≥ 65] = 0.19-0.75 (elderly; China)<br>Constraint: In China, parameter set only retained if adult parameter selected was greater than or equal to elderly parameter.	[7, 10, 31, 32, 37, 38]
ω	Risk of converting from non-infectious to infec- tious active case	Range: 0.007 - 0.02	[39]

Table S5: Parameters determining disease relapse and natural cure

Symbol	Description	Prior Range and Constraints	References
n_j	Risk of natural cure	Range: 0.1–0.25 (India)<br>Age stratified in China:<br>n[j<55] = 0.1–0.25<br>n[j 55-64] = (n[j<55] + n[j ≥ 65])/2<br>n[j ≥ 65] = 0.1–0.25<br>Constraint: In China, parameter set only retained if adult parameter selected was greater than or equal to elderly parameter.	[31, 32]
r_j	Risk of relapse from recovered to active (RR/MDR-)TB	r[j <15] = 0.01–0.07<br>r[j ≥ 15] = 0.01–0.07 (India)<br>r[j ≥ 15, <55] = 0.01–0.07 (China)<br>r[j ≥ 55, <65] = (r[j<55] + r[j ≥ 65])/2 (China)<br>r[j ≥ 65] = 0.01–0.07 (China)	[40–45]

Table S6: Parameters related to treatment initiation and treatment success

Parameter Symbol	Description	Prior Range and Constraints	References
κ	Treatment initiation rate, derived from case detection ratio (as per section 2.5.2).	NA	[19]
CDRscale_k	case detection ratio scaling factor in year k (see section	CDRscale[j = all] = (-)0.99 – (+)0.99 (India)<br>CDRscale[j≤ 15] = (-)0.99 – (+)0.99 (China)<br>CDRscale[j> 15, ≤ 54] = + (-)0.99 – (+)0.99 (China)<br>CDRscale[j ≥65] = + (-)0.99 – (+)0.99 (China)<br>CDRscale[j> 55, ≤ 64] = (CDRscale[j>65] + CDRscale[j> 15, ≤ 55])/2 (China)<br>CDRscale factors were applied to CDR as per scaling function f:<br>$f(\text{CDR}) = \begin{cases} \text{CDR} + (1 - \text{CDR}) \times cdrscale & \text{if } cdrscale \geq 0 \\ \text{CDR} + \text{CDR} \times cdrscale & \text{if } cdrscale < 0 \end{cases}$<br>In India, a single scaling factor was used across all age groups; in China, scaling factors for children, adults and elderly were used.<br>Constraints: In China, parameter sets only retained if values for elderly (j≤15) were lower than for adults.	[19]
emp_tx_p	Proportion empirically being started on RR/MDR-TB treatment in 2018	0-1	Assumed
e	Relative case-detection of non-infectious cases	0.4–0.8	Assumed
ppm	(India only) Proportion of private sector treatment among all treatment of DS-TB	0.37–0.66	Derived by calculation from sources [20, 21]

Table S7: Scale up of drug sensitivity testing coverage in the "Policy" scenario in China

Year	DST coverage
2018	35
2019	45
2020	50
2021	60
2026	70
2031	80
2036-	90

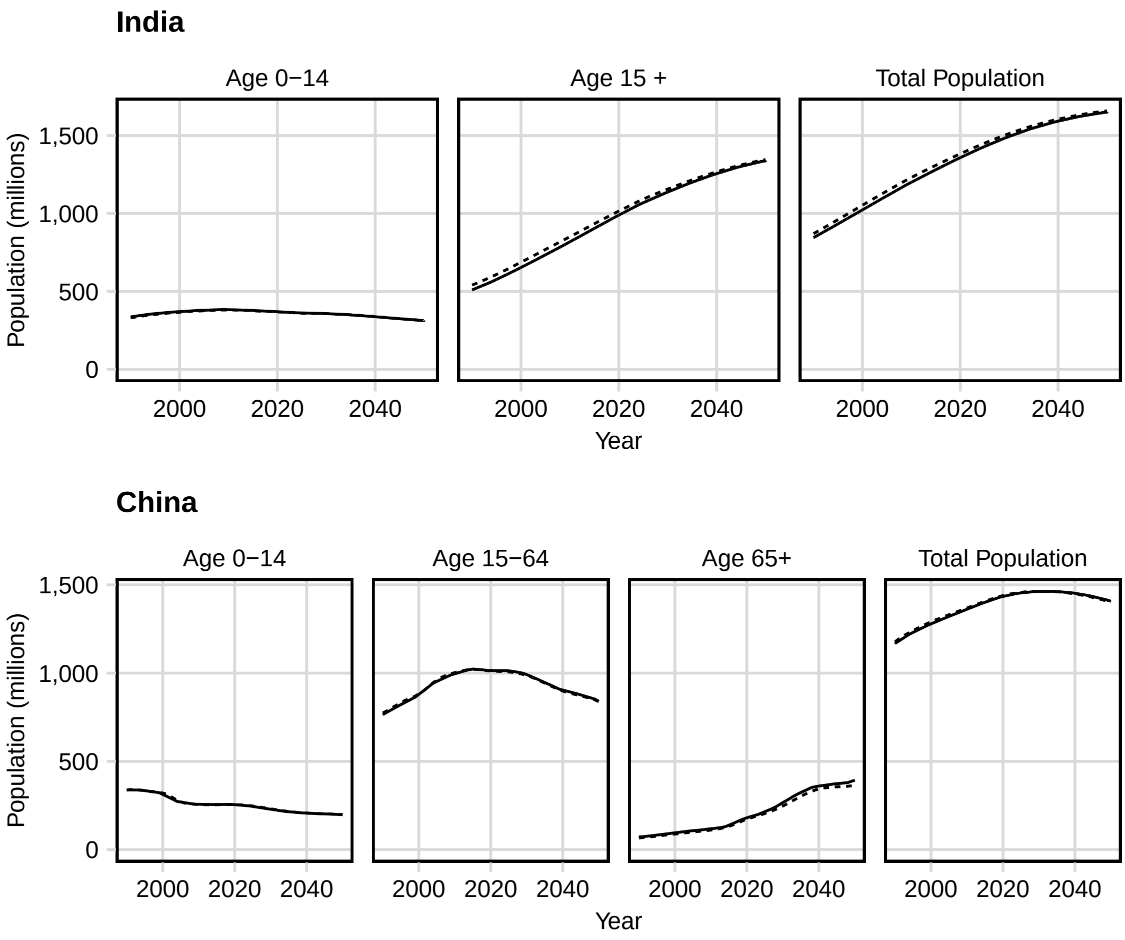

Figure S3: Demographic Model–India and China. Solid line represents median model population projection. Dashed lines represent UN ESA medium-estimate population projections until 2050. The age groups are those by which we stratified model calibration factors and targets.

mortality risk and these values were used as all-cause (background) mortality within the model. TB mortality was not removed from all-cause background mortality as its contribution was expected to be small. The median model trajectory for total population by age group as compared to UN ESA estimates are shown in Figure S3 for India and China.

Table S8: RR/MDR-TB treatment regimens in the China “Policy” scenario.

Length (months)	Regimen	2018	2019–2020	2021–2025	2026–2030	2031–2035	2036–
9	Short Course	0%	10%	30%	40%	40%	40%
24	R1[3]	0%	10%	30%	40%	50%	55%
	R2[4]	100%	80%	40%	20%	10%	5%

2.8 Transmission and Contact Mixing

Both TB and MDR-TB have age-specific epidemiologic patterns of incidence and age-dependent natural history parameters. Given this, we incorporated age-specific prevalence of infectious active disease and age-assortativity into the calculation of the age-dependent force-of-infection parameter λ (equations 6 and 7).

$$\lambda^S_{i,j} = q^S\left(1 - e^{-\sum_{y=1}^{y_{\max}} z \cdot D_{m,y} \cdot \frac{I^W_y}{N_y}}\right) \tag{6}$$

$$\lambda^R_{i,j} = q^R\left(1 - e^{-\sum_{y=1}^{y_{\max}} z \cdot D_{m,y} \cdot \frac{I^M_y}{N_y}}\right) \tag{7}$$

$$q^R = q^S \times \text{DR\_TS} \tag{8}$$

Here, i represents the time step; j is the age of interest, which suffers risk of infection; m is the broad ae class, in contact matrices, which contains the age j; y is the broad age class, in contact matrices, which contacta group m. There are $y_{\max}$ age classes; $D_{m,y}$ is the contact matrix, representing the average number of unique contacts by each member of y with members of group m; N_y is the total population in y, across age groups and compartments; I_y is the total number of infectious individuals in y across age all groups; z is the probability of transmission per infectious respiratory contact; andq^S, $q^R$and DR_TS are calibration factors described in section 2.4.

For the China case study we used an social contact matrix for China adapted by Harris, Sumner, Knight, et al. [11] and Harris [50], initially based contact data from a study based in Southwest China [29] of 1821 individuals divided among urban and rural areas. This study found strong assortativity among age-based contacts and similar total contacts between urban and rural residents.

2.8.1 India

For the India case study, we used the socialmixr R package [51] to adapt data from the POLYMOD [30] study to the population structure of India in 2015. We applied population estimates from the UN ESA [28]. The POLYMOD study includes the results of eight nationally representative prospective surveys across European countries, which estimated the daily contact patterns of individuals over the entire population age range. POLYMOD reported found contact patterns to be highly age-assortative, particularly among schoolchildren and young adults.

The social mixing model within the socialmixr package estimates the number of contacts made by an individual of age group i, with members of age group j per unit time (m_{ij}), leading to contact matrix D. The *total* number of contacts, a_i, made by i group individuals is scaled by an assortativity parameter, b_{ij}, reflecting a preference for contact with j group members and the proportion of the total population comprising group j, c_j. This gives

$$m_{ij} = [a_i] \cdot [b_{ij}] \cdot [c_j] \tag{9}$$

We collapsed the population in China into three age groups (age $\leq$ 14 years, age 15–64 years and age $\geq$ 65 years). First, we calculated *total* contact rates (a) for each group by aggregating data published in the original POLYMOD study per equations 11. We then computed the proportion of each age group j in the total POLYMOD study population, c_j and then back calculated assortativity parameters (b) from equation 9 to derive the assortativity matrix A (equation 9).

$$\mathbf{D} = \begin{array}{c} \quad\text{i} \qquad \text{j} \qquad \text{k} \\ \begin{bmatrix} m_{ii} & m_{ji} & m_{ki} \\ m_{ij} & m_{jj} & m_{kj} \\ m_{ik} & m_{jk} & m_{kk} \end{bmatrix} \end{array} \begin{array}{c} \\ \text{i} \\ \text{j} \\ \text{k} \end{array} \tag{10}$$

$$\begin{aligned} a_i &= m_{ii} + m_{ij} + m_{ik} \\ a_i &= m_{ji} + m_{jj} + m_{jk} \\ a_k &= m_{ki} + m_{kj} + m_{kk} \end{aligned} \tag{11}$$

$$\mathbf{A} = \begin{array}{c} \begin{matrix} \text{i} & \text{j} & \text{k} \end{matrix} \\ \begin{bmatrix} b_{ii} & b_{ji} & b_{ki} \\ b_{ij} & b_{jj} & b_{kj} \\ b_{ik} & b_{jk} & b_{kk} \end{bmatrix} \end{array} \begin{matrix} \text{i} \\ \text{j} \\ \text{k} \end{matrix} \tag{12}$$

We then re-applied each age group total contact rate, a_i, to the assortativity parameter of the contact group of interest, b_j and j group proportion (c_j) *for India* (from UN ESA data) to generate an asymmetric pair-wise contact matrix. Finally, we used our new estimated contact rates (m) and computed the total contacts made by groups i and j (equal in a perfect survey). To ensure that the total reciprocal *number* of contacts between any two groups was equal, we averaged these two values and re-computed new contact *rate* as per equation 13.

$$\begin{aligned} m_{ij} &= m_{av} \cdot N_i \\ m_{ji} &= m_{av} \cdot N_j \\ m_{\text{av}} &= \frac{(m_{ij} \cdot N_i) + (m_{ji} \cdot N_j)}{2} \end{aligned} \tag{13}$$

Here m_{av} is the average total number of contacts; N_i is the population in age group i; and N_j is the population in group j.

3 Model Calibration

3.1 Sampling and Calibration Method

We employed a two-stage calibration process to fit the model to calibration targets (section 3.2).

In the first stage, we utilised Approximate Bayesian Computation Accept-Reject Random sampling (ABC-RS) [52] to identify parameter space corresponding to a partial fit of the full calibration target set (>10 calibration targets). We used these partially fitted parameter sets as seeds to initialise an Approximate Bayesian Computation Markov Chain Monte Carlo (ABC-MCMC) [52, 53] rejection sampling process, to find parameter sets fitting incrementally greater numbers of calibration targets until all targets were satisfied. The parameter space was then further explored using the ABC-MCMC process to generate parameter sets fully compatible with the epidemiologic and health economic data. Within both the ABC-RS and ABC-MCMC sampling processes, we assumed uniform prior distributions for all parameters. We sampled along approximately 200 parallel Markov Chains each generating 40,000 samples. Finally, 1,000 fully fitted parameter sets were randomly selected from approximately 100,000 sets in India and 30,000 sets in China. We used these sets to generate 1,000 runs each for the Status Quo and Policy baseline scenarios. These 1,000 model runs captured uncertainty in TB natural history and costs for each baseline scenario. We then implemented the vaccine scenarios on each of these 1,000 runs for each baseline scenario to model vaccine impact.

3.2 Calibration Targets

We calibrated the model to China and India specific epidemiologic targets detailed in Tables S10 and S9 respectively.

3.2.1 India

We calibrated the India country model to seventeen calibration targets, four of which were specific to RR/MDR-TB (Table S9). These included prevalence, incidence, mortality and notification rates for all TB, and for incidence rate, laboratory confirmed RR/MDR-TB treatment initiations and proportion of RR/MDR-TB cases among notifications for all TB. Where data permitted, we calibrated to age-specific targets. Unless otherwise specified, calibration target quantities represent the same inter-compartmental transitions described in the China case study, above.

India has not reported the results of a nationally representative tuberculosis prevalence survey. Therefore, we used estimates of bacteriologically-positive prevalence rate derived through pooling subnational estimates [54] as a calibration target for all TB prevalence rate. We calibrated all TB incidence to WHO estimates of all-age all TB incidence rate. While age-stratified incidence is not reported by WHO nor by country authorities, model-based estimates of paediatric TB burden suggest that approximately 8% of incident TB in 2010 in India occured in children (age <15). Assuming the relative proportion of burden between children and adults remained the same between 2000 and 2017, we calculated age-specific incidence calibration targets from WHO overall incidence estimates and UN ESA [28] population estimates.

We calibrated to all TB mortality rate and age-specific notification rates per WHO estimates [19]. As discussed in section 2.5.1, we adjusted treatment initiation rates for the presence of the private sector, including private sector contributions towards case notifications, which are estimated to have risen from 0% in 2012 to approximately 20% of in 2017, as reported by WHO [22]. Further, a systematic review of case detection and patient retention throughout the tuberculosis "case-cascade" in the Indian Revised National Tuberculosis Control Programme has estimated that of 72% of prevalent TB patients who reach diagnostic centres, only 59% are subsequently registered on treatment. To allow for (1) uncertainty in the contribution of the private sector towards case notifications; and (2) losses between diagnosis and treatment initiation, we assumed a 20% uncertainty interval around WHO point-estimates of case notification data when constructing notification rate calibration targets.

The model was calibrated to RR/MDR-TB incidence rates as reported by WHO [22] and proportions of RR/MDR-TB cases among notified cases as reported in the first national anti-tuberculosis drug-resistance survey of India [25]. We calibrated the proportion of RR/MDR-TB treatment diagnosed through drug-sensitivity testing

($P_{dst}(t)$—section 2.5.2) to the reported number of laboratory confirmed RR/MDR-TB treatment initiations in 2017 [19].

3.2.2 China

We calibrated the China country model to twenty six calibration targets, six of which were specific to RR/MDR-TB epidemiology and treatment (Table S9). These included prevalence, incidence, mortality and notification rates for all TB and to RR/MDR-TB incidence rate, volume of RR/MDR-TB treatment in the CDC system, and proportion of RR/MDR-TB among new- and previously-treated case notifications. Where data permitted, we calibrated to age-specific targets.

We calibrated the prevalence rate of bacteriologically-positive tuberculosis (overall—including both drug-sensitive and drug-resistant), by age, to results from nationally representative prevalence surveys in 2000 and 2010 [24]. In this model, this represented the sum of I disease states across treatment history and drug-resistance strata.

Age-specific tuberculosis mortality rates for tuberculosis were provided by CDC [*Tao, L., personal communication*]; in the model, these represent disease specific mortality in the I, N, and T compartments. Although WHO estimates suggest a low case-fatality rate for TB in China [22], evidence suggests that up to 50% of pulmonary TB patients are attributed a non-TB cause of death, suggesting potentially high levels of misclassification [55, 56]. We therefore assumed a 50% uncertainty interval around mid-point estimates of mortality as calibration targets.

Notification rates represented treatment initiations (transitions from I or N compartments into T compartments. To account for overdiagnosis of bacteriologically negative tuberculosis in China, we reduced the contribution of bacteriologically-negative and/or clinically diagnosed case notifications to total case notification targets by 15%, per expert opinion [*Tao, L., CDC, personal communication*]. Case notifications reported by WHO before 2013 are disaggregated by bacteriologic status; for notification rate calibration targets before 2013, we directly reduced the sputum smear-negative notification value and total notifications to adjust for overdiagnosis. For targets including and beyond 2013, we reduced the value of clinically diagnosed new tuberculosis notifications and recalculated total notifications.

Patients in China can access TB care through either the CDC-based tuberculosis dispensary system, or through the parallel hospital-based system [27]. We assumed that case notification data only originated from the CDC system, which accounts for 80% of (all TB) treatment. Furthermore, evidence suggests that the screening algorithm utilised by China NTP may misclassify TB by up to 20% [11, 57]. Taken together, we applied a 20% uncertainty interval to age-stratified point-estimates of adjusted case notifications (as above) reported by WHO for China to derive final notification rate calibration targets.

Incidence (transitions *into* disease states I and N) was calibrated to WHO Global Tuberculosis Report [22] and Global TB database indidence data [19]. Incidence rates for RR/MDR-TB and RR/MDR-TB treatment volume were derived from WHO estimates [19, 22]. WHO estimates of incidence are derived from case notification data; following the case notification adjustment (above) we adjusted the corresponding values of all TB and RR/MDR-TB incidence rate. RR/MDR-TB incidence rate was adjusted using the WHO Global Tuberculosis Report method [22, 58] using equation 14 and data per Table S11.

$$I_{\mathrm{MDR}} = I\left((1-f)p_n((1-r)+r\rho)+fp_r\right) \tag{14}$$

Here f is the cumulative risk for incident cases to receive a non-relapse retreatment (following treatment failure or return after default); I is incidence of tuberculosis; I_{MDR} is incidence of MDR-TB; ρ is risk of RR/MDR-TB in relapses relative to previously untreated cases; p_n is proportion of RR/MDR-TB among new notifications; p_r is proportion of RR/MDR-TB among previously treated notifications; and r is proportion of relapses of the sum of new and relapse cases.

The number of RR/MDR-TB cases treated in the Chinese CDC system was derived from the WHO Global TB database [19] where we assumed 20% uncertainty interval, as for all TB case notifications. The proportion of RR/MDR-TB among case notifications was derived from nationally representative drug-resistance survey data [59, 60] and internal data from CDC [*Tao, L., personal communication*].

Table S9: Calibration targets—India. Rates are expressed per 100,000 population

Calibration target	Year	Subgroup	Target Range	References
**All TB**				
Prevalence rate	2015	Overall	195–312	[54]
Incidence rate	2000	Overall	149–473	[19, 61]
	2017	Overall	140–281	
	2000	0–14 Years	42–134	
	2017	0–14 Years	45–91	
	2000	15+ Years	192–609	
	2017	15+ Years	176–354	
Mortality rate	2017	Overall	29–34	[19]
Notification rate	2007	15+ Years	118–178	[19]
	2007	Overall	81–122	
	2017	15+ Years	139–209	
	2017	0–14 Years	6–9	
	2017	Overall	107–160	
**RR/MDR-TB**				
Incidence rate	2016	Overall	7–15	[22]
% Resistant among notified cases	2016	Never Treated	2–3	[25]
	2016	Previously Treated	10–13	
Lab confirmed RR/MDR-TB Treatments	2017	Overall	28,760–43,140	[19]

Table S10: Calibration targets—China. Rates are expressed per 100,000 population. CDC: Chinese Center for Disease Control and Prevention

Calibration target	Year	Subgroup	Target Range	References
**All TB**				
Prevalence rate	2000	Overall	163–195	[24]
		15–29 years	72–116	
		30–44 years	96–146	
		60+ years	510–609	
	2010	Overall	101–132	
		15–29 years	40–86	
		30–44 years	54–99	
		60+ years	106–168	
Incidence rate	2000	Overall	77–131	[19]
	2017	Overall	50–66	
Mortality rate	2010	Overall	1.36–4.07	
		0–14 years	0.06–0.18	
		15–64 years	0.88–2.65	
		65+ years	8.28–24.84	
Notification rate	2015	Overall	41.19–61.79	[19]
		0–14 years	1.22–1.84	
		15–64 years	43.9–65.85	
		65+ years	93.9–140.85	
**RR/MDR-TB**				
Incidence rate	2017	Overall	4.6–7	[22]
% Resistant among notified cases	2007	Never Treated	4.59–7.09	[59]
	2007	Previously Treated	21.73–29.98	
	2013	Never Treated	5.6–8.7	
	2013	Previously Treated	20–28	
CDC confirmed treatment initiations	2017	Overall	5,943–7,132	[19]

Table S11: Data values used to substantiate recalculation of adjusted TB incidence targets in China [58] China Incidence Target Data

Parameter	Mean	Standard Deviation
f	0.007745	0.00183
r	0.02983	0.002473
ρ	3.377	0.3759
p_n	0.0713	0.00801
p_r	0.2408	0.01918

4 Vaccine implementation

To model vaccination, we duplicated both drug resistance and treatment history strata and moved immunised populations from their state in the unvaccinated stratum to the corresponding state in the vaccinated stratum. Similarly, when the effect of the vaccine waned (loss of protection), these populations moved in the reverse direction, from a given state in the vaccinated strata to the corresponding state in the unvaccinated strata (equation 2).

We implemented a "Prevention of Disease" (PoD)-type vaccines, conferring protection against the development of active TB disease. We did not model a "Prevention of Infection", PoI, vaccine effect—infection by *M. tb* and transmission was identical vaccinated and unvaccinated strata. To model a PoD vaccine, we multiplied the following model parameters by a factor equal to (1 — vaccine efficacy):

1. Primary ("fast") progression from the susceptible state (p);
2. Reactivation from the latently infected state (v);
3. Relapse from the resolved state (r).

This represented a "*leaky*" type vaccine, wherein disease continues to manifest in vaccine recipients, albeit at a rate reduced in proportion to the efficacy of the vaccine.

We modelled three subtypes of PoD vaccines, whose effect depended on the extant host infection status at the time of vaccination: (1) "pre-infection" (PRI) vaccines were only effective in susceptible individuals; conversely, "post-infection" (PSI) vaccines were effective in those with latent infection and resolved infection; "pre- and post-infection" (P&PI) vaccines were effective in all three types of host infection status (Table S12). Vaccines did not affect treatment related parameters (detection, treatment success or failure rates), natural cure rates nor TB related mortality. Waning (loss of protection) occurred instantly and exactly at the end of the duration of protection.

We did not explicitly represent existing Bacillus Calmette–Guérin (BCG) immunisation programmes as we assumed protection conferred by BCG to be reflected in calibration targets.

4.1 Vaccine Characteristics

There is no currently no licensed adult vaccine to prevent tuberculosis. Two candidates in advanced clinical development—M72/AS01$_E$ and BCG revaccination[5]—have reported efficacies of 49.7% and 45.4% at 3 and 2 years follow up, respectively [62, 63]. BCG revaccination was administered to IFNγ negative populations, whereas M72/AS01$_E$ was administered to IFNγ positive populations. Additionally, WHO preferred product characteristics (PPC) for new tuberculosis vaccines [64] specifies a minimum duration of protection of at least 10 years, with a minimum efficacy of 50%. To encompass the WHO PPC specification, BCG revaccination and M72/AS01E, we modelled vaccines conferring protection for 5- and 10-years, with efficacy between 30–90% in 20% increments, across PRI, PSI and P&PI vaccine types.

[5]Revaccination administered to adults

Table S12: Modelled vaccine types and impact on disease states

**Vaccine type**	**States which vaccine is applied to (and effective in)**			
	**Susceptible (S)**	**Latent (L)**	**Active Disease (I or NI)**	**Recovered (R)**
Pre-infection (PRI)	Yes	No	No	No
Post-Infection (PSI)	No	Yes	No	Yes
Pre- and Post-infection (P&PI)	Yes	Yes	No	Yes

4.2 Deployment

Previous modelling studies suggest that age-targeting vaccination—to adults, adolescents or the elderly—is likely to achieve a greater impact on all TB burden than infant or early childhood immunisation [11, 12]. This is reflected in the WHO PPCs for new TB vaccines [64] which consider these populations the priority target for TB vaccine development. However, to date, there are no major adult diseases against which large-scale routine vaccination is administered to serve as a direct analogue to model adult TB vaccine programmes.

We assumed vaccine administration through two strategies, routine vaccination and mass campaigns, both beginning in 2027. We generated assumptions around vaccine coverage based on immunisation programmes for other diseases, applied to similar age groups in other settings.

We assumed continuous routine TB vaccination was delivered to children aged 9 with 80% coverage, along with human papillomavirus vaccine (HPV). The routine coverage estimate was based on secondary school enrolment rates in China and India, and HPV vaccine programme coverage among schoolchildren in South Africa [65, 66].

Mass campaigns were delivered to ages 10 and above at a 10-yearly frequency. Menafrivac campaigns delivered to 1–29 year olds in South Africa were reported to achieve coverage of 70–98% [67]. However, routine vaccination for influenza in China [68, 69]and mass adult campaigns for Japanese encaphalitis in India [70]—both of which were delivered to populations including the elderly—have reported coverage estimates of 36–49% and 58%, respectively. As our mass campaign age-group was wide, including the elderly, we based our mass campaign coverage (70%) on the lower bound of the Menafrivac coverage estimate. In addition, we simulated mass vaccination campaigns at 30% coverage as an additional conservative scenario analysis.

5 Cost-effectiveness Analysis

We calculated total cost from a public healthcare sector perspective as comprised of total tuberculosis programme costs (section 5.1) and vaccine programme costs (section 5.2). We then derived Disability Adjusted Life Years (DALYs) incurred due to active tuberculosis disease in the (unvaccinated) baseline scenarios and their corresponding vaccinated scenario and calculated the difference as the health benefit of vaccination (section 5.3). We derived the incremental cost-effectiveness ratio of vaccination (section 5.4) as a measure of cost-effectiveness.

5.1 TB-related Cost Model

The unit costs of TB management are summarised in Table S13 fo India and China. We estimated costs from a health service perspective using an ingredient approach [71]. To inform the cost calculations, we obtained unit costs for DS- and RR/MDR-TB diagnosis, drug-sensitivity testing and DS- and RR/MDR-TB treatment. In addition, for India, we added the estimated cost of incentives provided *to* the private sector to improve case notification and cost of providing nutritional support to patients on treatment for tuberculosis (in both private and public sectors). The annual total service delivery cost was calculated as the sum of unit costs incurred each year. We assumed a top-up of 50% of assumed programme cost, based on the national expenditure report to the WHO TB programme [22].

The annual total cost was calculated as the sum of the unit cost per output multiplied by the quantity of outputs each year. The outputs included:

1. Number of persons with presumptive TB tested, calculated from the number of people diagnosed;

2. Number of person-months of drug sensitive TB treatment;

3. Number of drug sensitivity tests conducted;

4. Number of people started on drug resistant TB treatment; and

5. Number of person-months of drug resistant TB treatment.

To calculate costs of diagnosis, we multiplied the unit costs for diagnosis by the number of people tested. The number of people tested was calculated using a Test-to-Diagnosis Ratio (TDR), to adjust for false-negative and true-negative test results. TDR values of 3.57 and 6.48 were applied to China [72] and India [26] in country-specific starting years 2011 and 2016, respectively. We then adjusted the TDR value in each subsequent year by the prevalence of active tuberculosis.

5.2 Vaccine-related Cost Model

We separated vaccine-related costs into vaccine, delivery and program costs. The unit costs are summarised in Table S14.

In India, we used a national analysis of variation in cost and performance of routine immunisation service delivery[73] to derive uncertainty ranges for routine vaccine delivery cost. We used estimates from the Indian measles-rubella vaccination campaign operational guidelines [74] to add delivery costs in mass campaigns.

In China, delivery costs were sourced from literature [75, 76]. We assumed delivery costs per person immunised to be the same in mass campaign or routine settings. Programme costs associated with mass campaigns were estimated from a study of nationwide catch-up vaccination for hepatitis B [77].

In both countries, we modelled a USD 10 and USD 30 price per vaccine based on expert opinion. We calculated the annual total cost as the sum of the unit cost per vaccine multiplied by quantity of vaccines delivered plus programme costs.

[6] Additional costs for first- and RR/MDR-TB treatment received from CDC [Tao, L., personal communication]

Table S13: TB-related Unit Costs

Country	Cost	Value	[range] (USD)	Distribution	Source
India	DSTB diagnosis, per patient	14.82	[11.86–17.79]	Normal	[78–80]
	RR/MDR-TB diagnosis, per patient (assumed to be DSTB diagnosis cost +20%)	17.78	[14.23–21.35]		Assumed
	Drug sensitivity testing, per patient	6.00	[4.80–7.20]	Normal	[80]
	DS-TB treatment, per patient-month	26.43	[21.15–31.71]	Normal	[81–83]
	RR/MDR-TB treatment, per patient-month	216.19	[187.88–244.50]	Normal	[81–83]
	Patient nutritional support	7.46			[26]
	Private sector incentive	3.73			[26]
China[6]	DSTB diagnosis, per patient	27.05	[21.63–32.47]	Normal	[84, 85]
	RR/MDR-TB diagnosis, per patient (assumed to be DSTB diagnosis cost +20%)	32.46	[25.96–38.96]	Normal	Assumed
	Drug sensitivity testing, per patient	13.20	[11.78–14.62]	Normal	[86]
	DS-TB treatment, per patient-month	28.41	[27.14–29.68]	Normal	[48, 84]
	RR/MDR-TB treatment—no injectables, per patient-month	746.84	[377.29–861.73]	β	[48, 84]
	RR/MDR-TB treatment—including bedaquiline, per patient-month	346.29	[276.77–413.84]	β	[48, 84]

Table S14: Vaccine-related Costs

Country	Cost	Value	[range] (USD)	Distribution	Source
India	Delivery cost per regimen (routine)	1.88	1.13–2.40	β	[73]
	Delivery cost per regimen (mass)	1.95	1.20–2.47	β	[74]
	Vaccine campaign cost (fixed)	25,374,949.00			[74]
China	Delivery cost per regimen (routine)	2.32	1.60–2.80	β	[75, 76]
	Delivery cost per regimen (mass)	2.32	1.60–2.80	β	[75, 76]
	Vaccine delivery cost (variable cost per 10,000 vaccinated through mass campaigns)	16,133.10			[77]

Table S15: Willingness to Pay Thresholds

	Threshold	Value	% GDP
China	WHO[7]	9771	100
	HCOC (lower)	3650	45
	HCOC (upper)	5669	71
India	WHO	2016	100
	HCOC (lower)	264	17
	HCOC (upper)	363	23

5.3 Disability Adjusted Life Years Calculations

To calculate benefits associated with vaccination, we calculated the difference in total Disability Adjusted Life Years (DALYs) associated with tuberculosis infection between each vaccine and each of the two baseline scenarios. We used the disability weight (0.333) for tuberculosis as reported in the Global Burden of Disease 2013 study [87] and calculated total DALYs incurred as per WHO CHOICE [88].

5.4 Cost-effectiveness Analysis and Willingness to Pay Thresholds

We calculated the incremental cost effectiveness ratio as the ratio between the incremental benefit, in DALYs averted, and the incremental cost, in USD, for each run across vaccination and baseline scenario. Both costs and benefits were discounted to 2027 (when vaccination begain) at 3%, per the Gates Reference Case for Economic Evaluation [89]. We analysed cost-effectiveness by 2050, reflecting a 23 year timeframe in line with WHO END TB [90] and UN SDG TB control targets [91]. We constructed a cost-effectiveness acceptability curve for each vaccine profile, per country and per baseline scenario and present an estimated probability of vaccine cost-effectiveness against a continuous willingness-to-pay (WTP) threshold. We report probability of cost-effectiveness against three WTP thresholds from the literature (Table S15):

1. WHO threshold—1 times gross domestic product (GDP) per capita [84, 92]

2. Healthcare opportunity cost (HCOC) based threshold [93]

[7] WHO values represent 2018 World Bank GDP per capita estimates.

Part II

Furthers Results and Discussion

6 Calibration and Baseline Scenario Projections

6.1 Posterior Distributions of Parameters

Posterior distributions for parameters sampled and described in sections 2.4 and 2.5 are presented here for India (Figures S4 and S5) and China (S6 and S7).

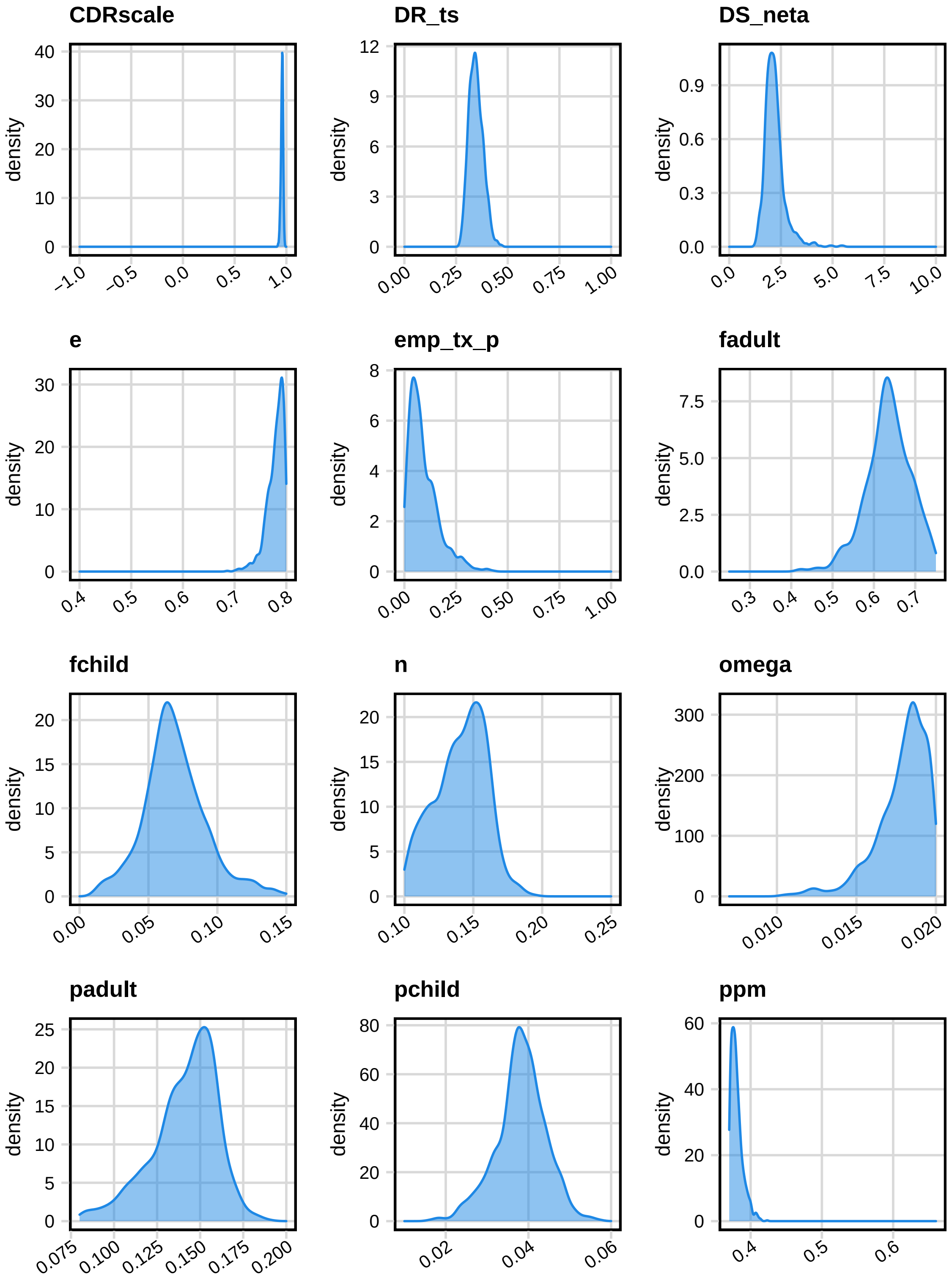

Figure S4: Posterior distributions of sampled parameters—India. Subplots show probability density of parameter values (y-axis) plotted against the prior range (x-axis) of each parameter. Parameter names suffixed with *adult or A* indicate adult specific parameters. Suffixes *child* or *C* indicate children-specific sampled parameters. **CDRscale**: case detection ratio scaling factor ($cdrscale$). **DR_ts**: RR/MDR-TB relative transmission fitness cost. **DS_neta**: transmission scaling factor for all TB. **e**: relative probability of case detection of non-infectious TB. **emp_tx_p**: proportion empirically diagnosed and treated for RR/MDR-TB. **fadult and fchild**: proportion fast progressing to infectious active TB. **n:** natural cure; **omega:** risk of converting from non-infectious to infectious TB. **padult and pchild**: proportion fast progressing to active TB. **ppm**: proportion of all TB treated in private sector.

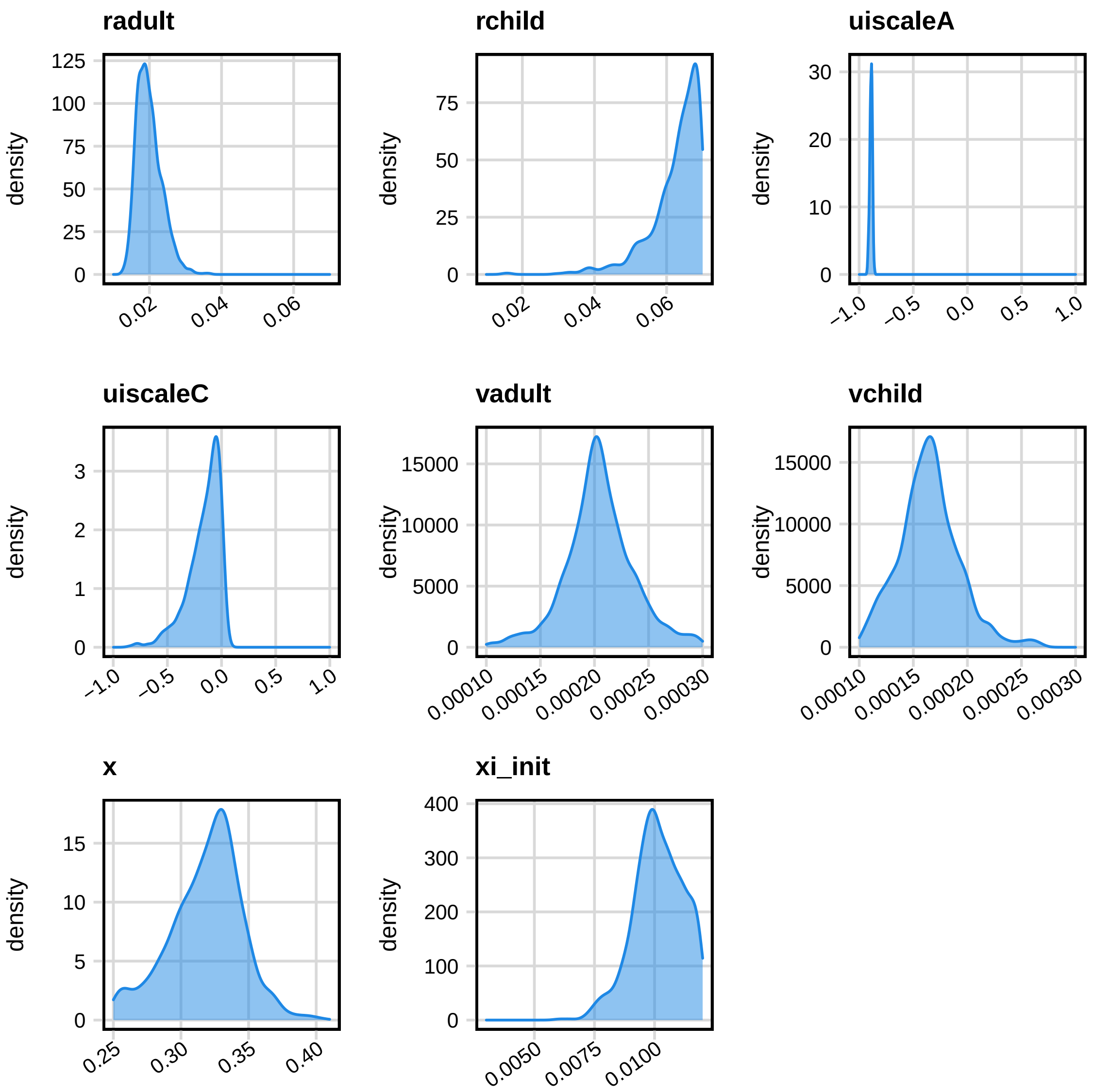

Figure S5: Posterior distributions of sampled parameters—India (contd.). Subplots show probability density of parameter values (y-axis) plotted against the prior range (x-axis) of each parameter. Parameter names suffixed with *adult or A* indicate adult specific parameters. Suffixes *child* or *C* indicate children-specific sampled parameters. **radult and rchild**: r, risk of relapse from resolved disease. **uiscaleA and uiscaleC**: TB mortality scaling factors. **vadult and vchild**: v, risk of relapse from latent disease. **x**: x, relative protection against reinfection in latent and resolved compartments; **xi_init**: ξ, risk of acquiring drug resistance on-treatment for DS-TB.

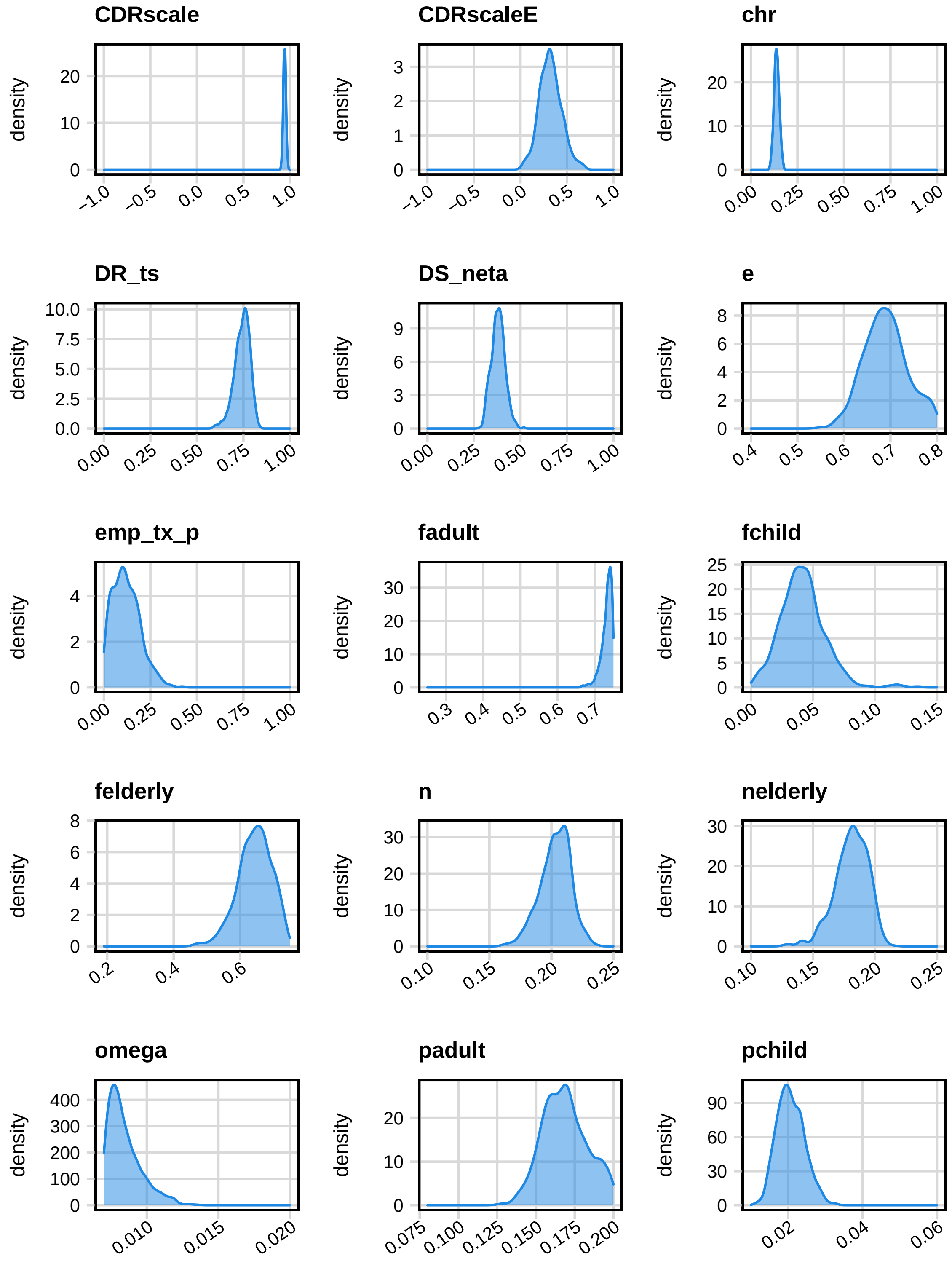

Figure S6: Posterior distributions of sampled parameters—China. Subplots show probability density of parameter values (y-axis) plotted against the prior range (x-axis) of each parameter. Parameter names suffixed with *adult or A* indicate adult specific parameters. Suffixes *child* or *C* indicate children-specific sampled parameters. Suffixes *elderly* or *E* indicate elderly-specific parameters. **CDRscale and CDRscaleE**: case detection ratio scaling factor (*cdrscale*). **chr**: proportion of RR/MDR-TB treatment in CDC system. **DR_ts**: RR/MDR-TB relative transmission fitness cost. **DS_neta**: transmission scaling factor for all TB. **e**: relative probability of case detection of non-infectious TB. **emp_tx_p**: proportion empirically diagnosed and treated for RR/MDR-TB. **fadult, fchild and felderly**: proportion fast progressing to infectious active TB. **n and nelderly:** natural cure; **omega:** risk of converting from non-infectious to infectious TB. **padult and pchild**: proportion fast progressing to active TB.

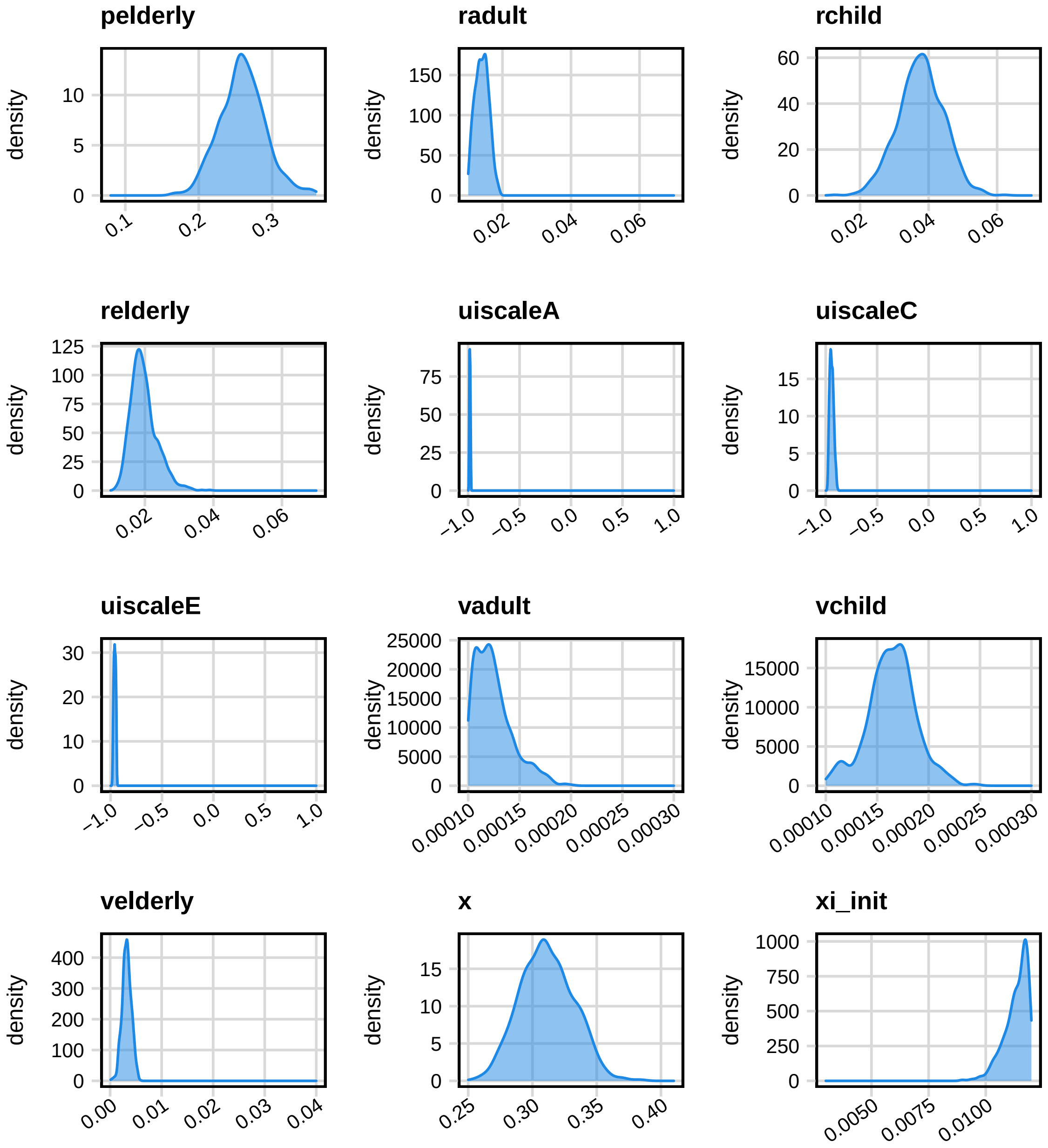

Figure S7: Posterior distributions of sampled parameters—China (contd.). Posterior distributions of sampled parameters—India (contd.). Subplots show probability density of parameter values (y-axis) plotted against the prior range (x-axis) of each parameter. Parameter names suffixed with *adult or A* indicate adult specific parameters. Suffixes *child* or *C* indicate children-specific sampled parameters. **pelderly**: proportion fast progressing to active TB. **radult, rchild relderly**: r, risk of relapse from resolved disease. **uiscaleA, uiscaleC and uiscaleE**: TB mortality scaling factors. **vadult, vchild and velderly**: v, risk of relapse from latent disease. **x**: x, relative protection against reinfection in latent and resolved compartments; **xi_init**: ξ, risk of acquiring drug resistance on-treatment for DS-TB.

6.2 India

6.2.1 Calibration

Calibration results are summarised in Figures S8 and S9.

We calibrated the model to all prespecified targets.

All TB notification rates were predicted to be 103 [UI 86–122] per 100,000 and 142 [UI 118–169] per 100,000 among all ages and adults (age>14) in 2007, and 119 [UI 107–131] per 100,000, 24 [UI 23–27] per 100,000 and 156 [UI 140–174] per 100,000 among all ages, children (age<15) and adults (age>14) in 2017, respectively (Figure S8A). All TB incidence rates for all ages, children (age<15) and adults (age>14) were predicted to be 339 [UI 292–373] per 100,000, 104 [UI 91–107] per 100,000 and 450 [UI 381–498] per 100,000 in 2010, and 261 [UI 216–273] per 100,000, 65 [UI 60–74] per 100,000 and 339 [UI 278–354] per 100,000 in 2017, respectively (Figure S8B). All TB mortality rate was predicted to be 31 [UI 29–34] per 100,000 in 2017 (Figure S8C). The model predicted all TB prevalence rate to be 218 [UI 195–310] per 100,000 in 2015 (Figure S8D).

The model predicted RR/MDR-TB incidence to be 8 [UI 7–10] per 100,000 in 2017 (Figure S9A). Laboratory confirmed RR/MDR-TB treatments were predicted to be 37,184 [UI 28,841–43,133] in 2017 (Figure S9B). The proportion of RR/MDR-TB cases among all TB notifications in 2016 was predicted to be 2.7% [UI 2.0–3.0] among never-treated cases and 11.0% [UI 10.0–13.0] among previously-treated cases, respectively (Figure S9C).

6.2.2 Baseline Scenario Projections

Model projections for overall and RR/MDR-TB epidemiology for India over 2018–2050 are presented in Figure S10.

At baseline, with no new vaccine and no change to programmatic management of TB after 2018 (the "Status Quo" baseline scenario), the model projected all TB prevalence rate at 216 [UI 191–301] per 100,000 in 2018 and 237 [UI 191–341] per 100,000 in 2050 (Figure S10A). Overall incidence was predicted to be 262 [UI 217–274] per 100,000 in 2018 and 280 [UI 217–334] per 100,000 (Figure S10B) 2050. Overall mortality was predicted to be 31 [UI 29–34] per 100,000 in 2018 and 32 [UI 25–40] per 100,000 in 2050 (Figure S10C). RR/MDR-TB incidence was predicted to be 8 [UI 7–10] per 100,000 in 2018 and 11 [UI 7–25] per 100,000 (Figure S10D). RR/MDR-TB mortality rate was predicted at 2 [UI 2–3] per 100,000 in 2018 and 3 [UI 2–5] per 100,000 in 2050 (Figure S10E). The proportion of RR/MDR-TB cases among never treated TB notifications was predicted to be stable at 3% [UI 2–3] in 2018 and 4% [UI 2–7] in 2050; the corresponding proportion among previously treated TB notifications was predicted to be 10% [UI 9–13] in 2018 and 13% [UI 10–23] in 2050 (Figure S10F).

In the "Policy" baseline scenario with scaled up programmatic TB management, all TB burden declined and then plateaued compared to 2018. All TB prevalence rate, incidence rate, and mortality in 2050 were predicted to be 180 [UI 136–268] per 100,000 (Figure S10A), 238 [UI 183–292] per 100,000 (Figure S10B) and 24 [UI 19–31] per 100,000 (Figure S10C) respectively. RR/MDR-TB incidence and mortality were predicted to be 7 [UI 5–15] per 100,000 (Figure S10D) and 2 [UI 1–3] per 100,000 (Figure S10E) by 2050 respectively. The proportion of RR/MDR-TB among never treated and previously treated notifications in 2050 was predicted to be 3% [UI 2–5] and 7% [UI 6–14] respectively (Figure S10F).

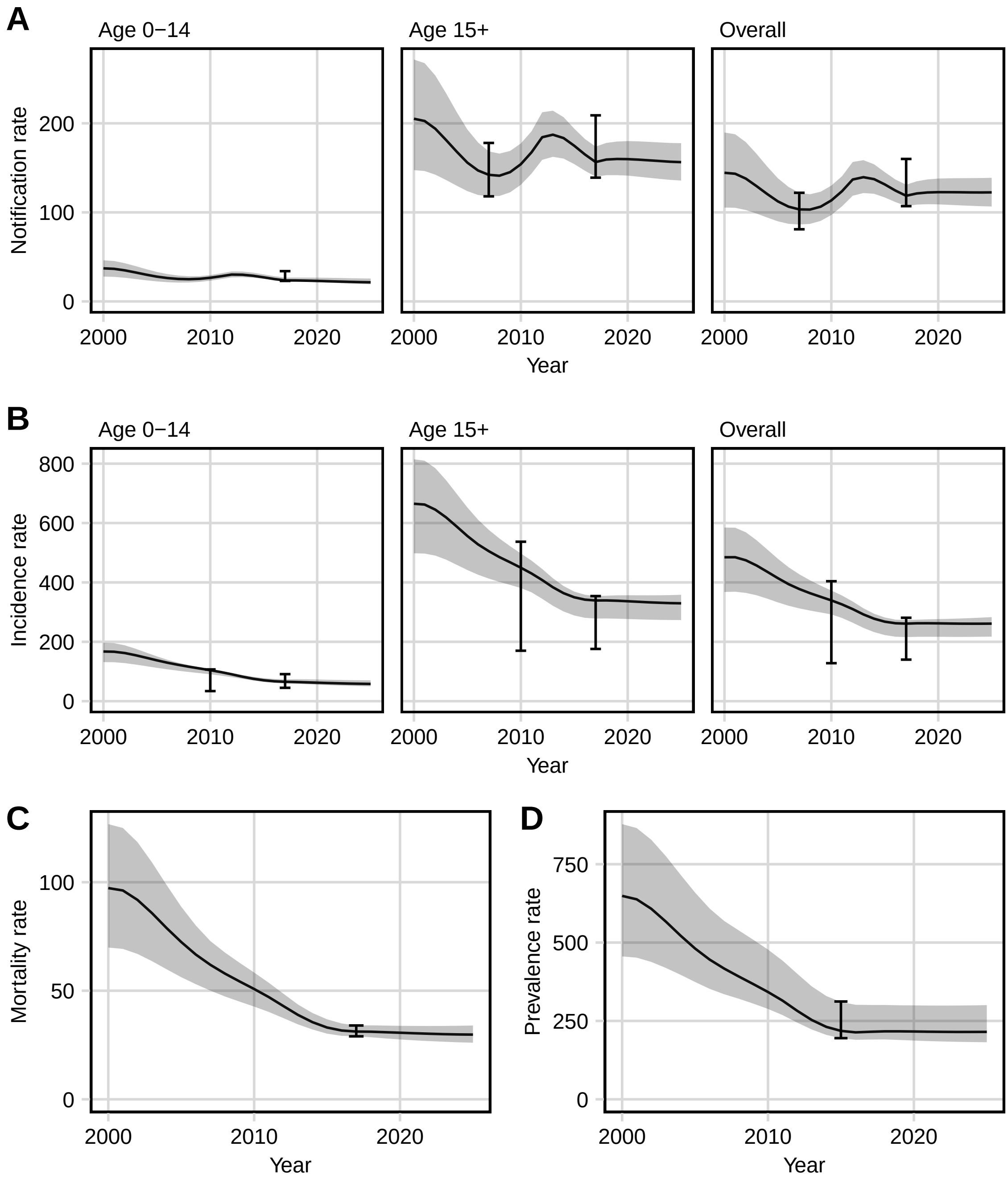

Figure S8: Calibration Results–All Tuberculosis in India. **A**: TB case notifications. **B**: Incidence rate for all TB. **C**: TB mortality. **D**: TB prevalence. All rates are presented per 100,000 population. Line represents median trajectory; ribbons represent minimum and maximum trajectories and error bars represent calibration targets.

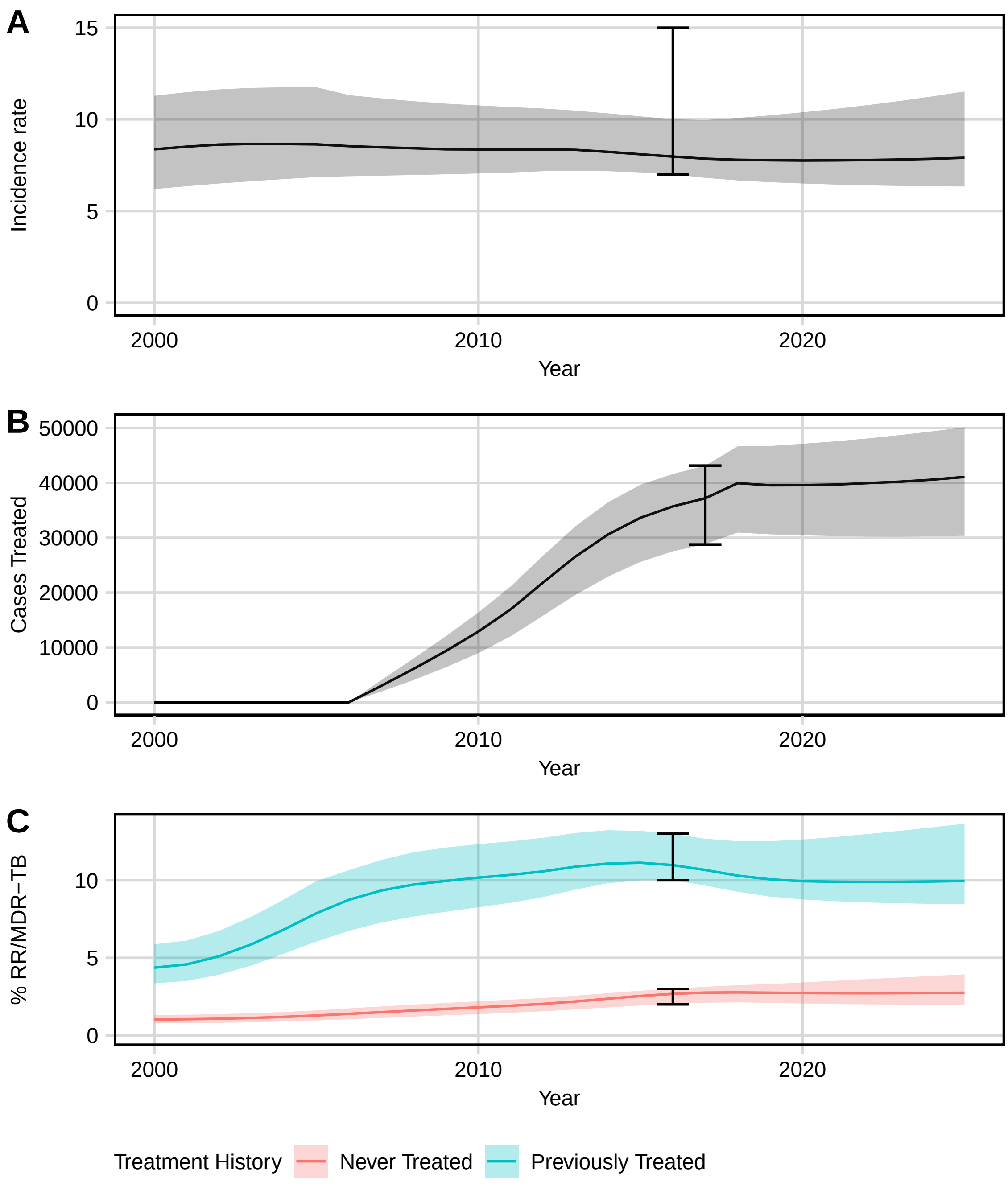

Figure S9: Calibration Results—RR/MDR-TB in India. **A**: RR/MDR-TB incidence rate. **B**: Number of laboratory confirmed treatments for RR/MDR-TB in the public sector. **C**: % of RR/MDR-TB among all TB case notifications, disaggregated by previous treatment. Line represents median trajectory; ribbons represent minimum and maximum trajectories and error bars represent calibration targets.

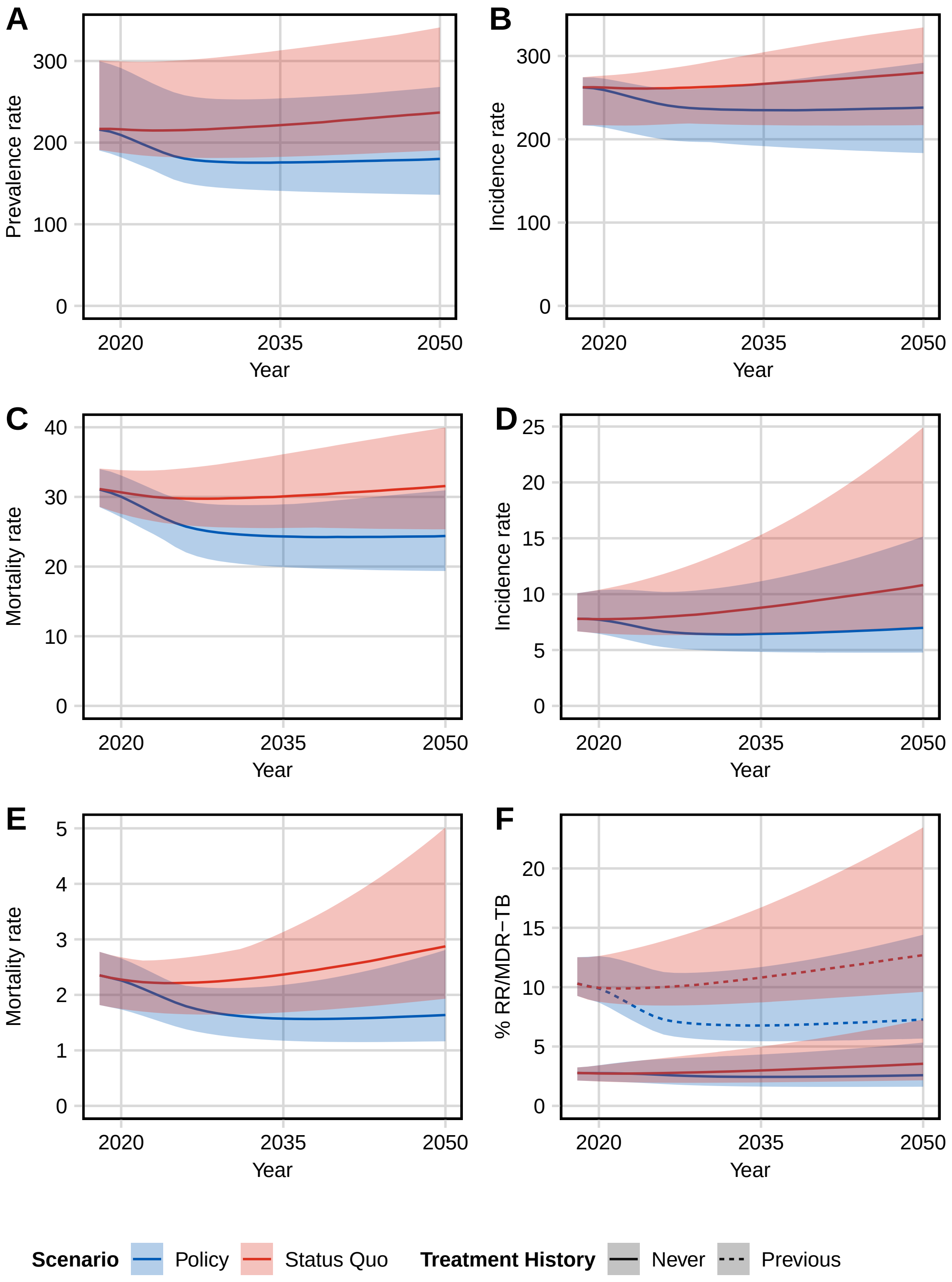

Figure S10: Baseline (no vaccine) projections–India. **A:** All TB Prevalence rate. **B**: All TB Incidence rate. **C**: All TB mortality rate. **D**: RR/MDR-TB incidence rate. **E**: RR/MDR-TB mortality rate. **F**: Proportion RR/MDR-TB cases among notifications, stratified by treatment history. All rates are expressed per 100,000 population.

6.3 China

6.3.1 Calibration

The model predicted prevalence rates of all TB for all adults (age >14), ages 15-29, ages 30-44, ages 45-59 and ages >59 of 178 [UI 168–187] per 100,000, 82 [UI 72–92] per 100,000, 132 [UI 121–146] per 100,000, 179 [UI 174–195] per 100,000 and 531 [UI 510–564] per 100,000 in 2000 and 178 [UI 168–187] per 100,000, 82 [UI 72–92] per 100,000, 132 [UI 121–146] per 100,000, 179 [UI 174–195] per 100,000 and 531 [UI 510–564] per 100,000 in 2010 respectively (Figure S11A). All TB incidence rates were predicted at 83 [UI 77–93] per 100,000 and cninc2017 in 2000 and 2017 respectively (Figure S11B).

All TB notification rates in 2010 were predicted at 47 [UI 43–56] per 100,000, 2 [UI 1–2] per 100,000, 52 [UI 48–65] per 100,000 and 100 [UI 90–134] per 100,000 for across all ages, in children (age < 15), adults (age > 14 & and < 65) and elderly (age > 64), respectively (Figure S12A and C). All TB mortality rates in 2010 were predicted at 3 [UI 1–4] per 100,000, 0.1 [UI 0.1–0.2] per 100,000, 1.6 [UI 0.9–2.6] per 100,000 and 17 [UI 8–25] per 100,000 for across all ages, in children (age < 15), adults (age > 14 & and < 65) and elderly (age > 64), respectively (Figure S12B and D).

The model predicted RR/MDR-TB incidence to be 5 [UI 4–6] per 100,000 in 2017 (Figure S13A). RR/MDR-TB treatment by the Chinese Center for Disease Control and Prevention were predicted to be 6,544 [UI 5,943–7,131] in 2017 (Figure S13B). The proportion of RR/MDR-TB cases among all TB notifications was predicted to be 4.9% [UI 4.6–5.9] among never-treated cases in 2007, 22.6% [UI 21.7–26.0] among previously-treated cases in 2007, 7.6% [UI 6.7–8.6] among never-treated cases in 2013, and 26.5% [UI 23.0–28.0] among previously-treated cases in 2013 respectively (Figure S13C).

6.3.2 Baseline Scenario Projections

Model projections for overall and RR/MDR-TB epidemiology for China over 2018–2050 are presented in Figure S14.

At baseline, with no new vaccine and no change to programmatic management of TB after 2018 (the "Status Quo" baseline scenario), the model projected all TB prevalence rate at 76 [UI 74–79] per 100,000 in 2018 and 86 [UI 77–94] per 100,000 in 2050 (Figure S14A). All TB incidence rate was predicted to be 64 [UI 63–65] per 100,000 in 2018 and 62 [UI 59–66] per 100,000 in 2050 (Figure S14B). All TB mortality rate was predicted to be 2.1 [UI 1.4–2.8] per 100,000 in 2018 and 2.8 [UI 1.8–4.0] per 100,000 in 2050 (Figure S14C). RR/MDR-TB incidence rate was predicted to be 5 [UI 5–6] per 100,000 in 2018 and 15 [UI 12–17] per 100,000 in 2050 (Figure S14D). RR/MDR-TB mortality rate was predicted to be 0.2 [UI 0.2–0.4] per 100,000 in 2018 and 0.8 [UI 0.5–1.2] per 100,000 in 2050 (Figure S14E). The proportion of RR/MDR-TB cases among never treated TB notifications was predicted to be 10% [UI 9–11] in 2018 and 28% [UI 23–30] in 2050; correspondingly, the proportion among previously treated TB notifications was predicted to be 27% [UI 26–28] in 2018 and 50% [UI 44–53] in 2050 (Figure S14F).

In the "Policy" baseline scenario with scaled up programmatic TB management, all TB burden remained relatively stable compared to 2018. All TB prevalence, incidence and mortality rates in 2050 were predicted to be 74 [UI 67–80] per 100,000, 56 [UI 54–60] per 100,000 and 2.5 [UI 1.6–3.5] per 100,000 respectively (Figure S14A-C). RR/MDR-TB incidence and mortality rates were predicted to be 9 [UI 7–9] per 100,000 and 0.5 [UI 0.3–0.7] per 100,000 by 2050 respectively (Figure S14D and E). The proportion of RR/MDR-TB among never treated and previously treated notifications in 2050 was predicted to be 17% [UI 14–19] and 32% [UI 25–33] respectively (Figure S14F).

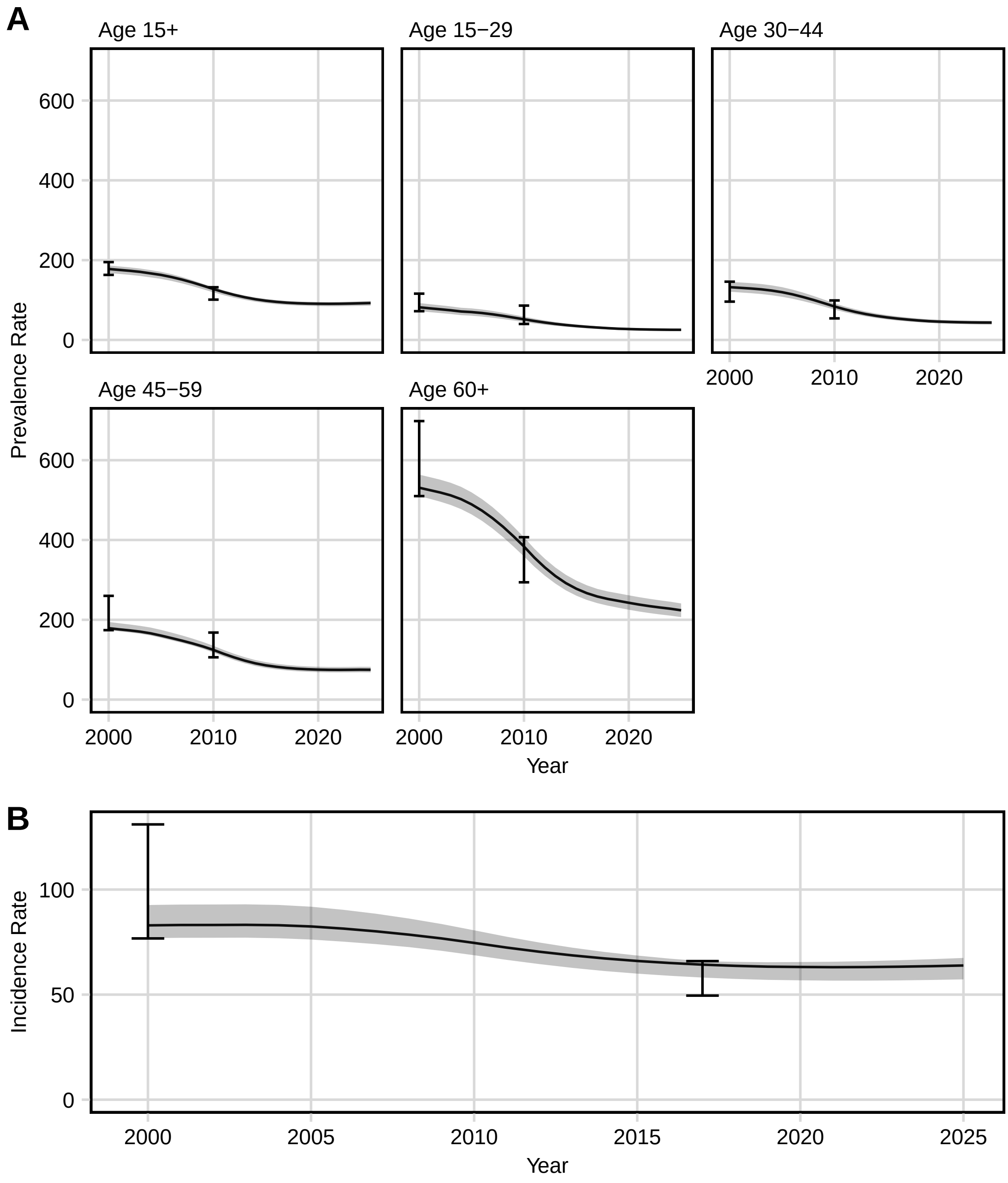

Figure S11: Calibration Results—All TB in China. **A**: All TB prevalence rate, by age. **B**: All TB incidence rate, all ages. All rates are presented per 100,000 population. Line represents median trajectory; ribbons represent minimum and maximum trajectories and error bars represent calibration targets.

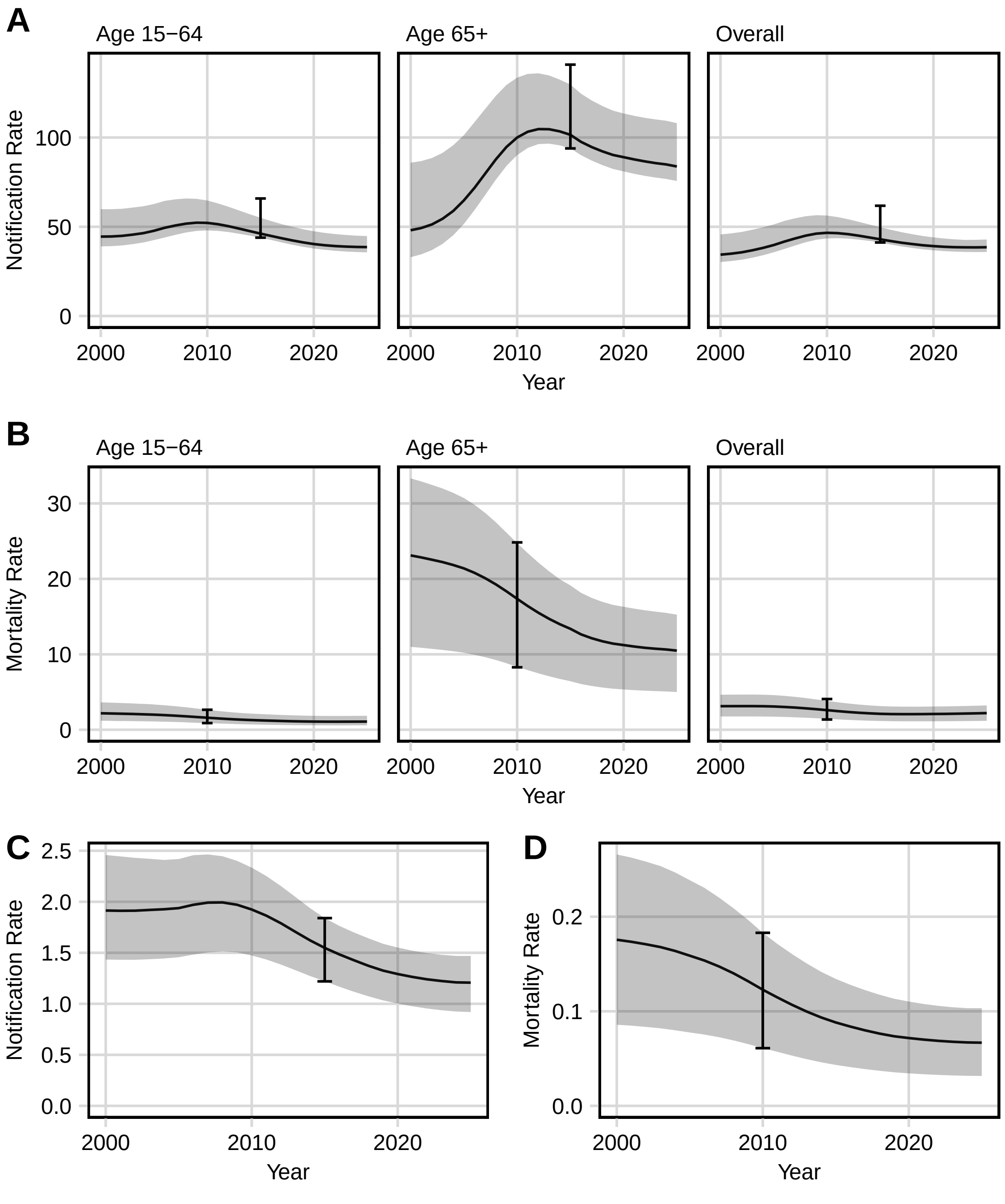

Figure S12: Calibration Results—Notifications and Mortality of All TB in China. **A**: All TB notification rate, by age. **B**: All TB mortality rate, by age. **C**: Notification rate for **Age**≤ 14 **years**. **D**: Mortality rate for **Age**≤ 14 **years**. All rates are presented per 100,000 population. Line represents median trajectory; ribbons represent minimum and maximum trajectories and error bars represent calibration targets.

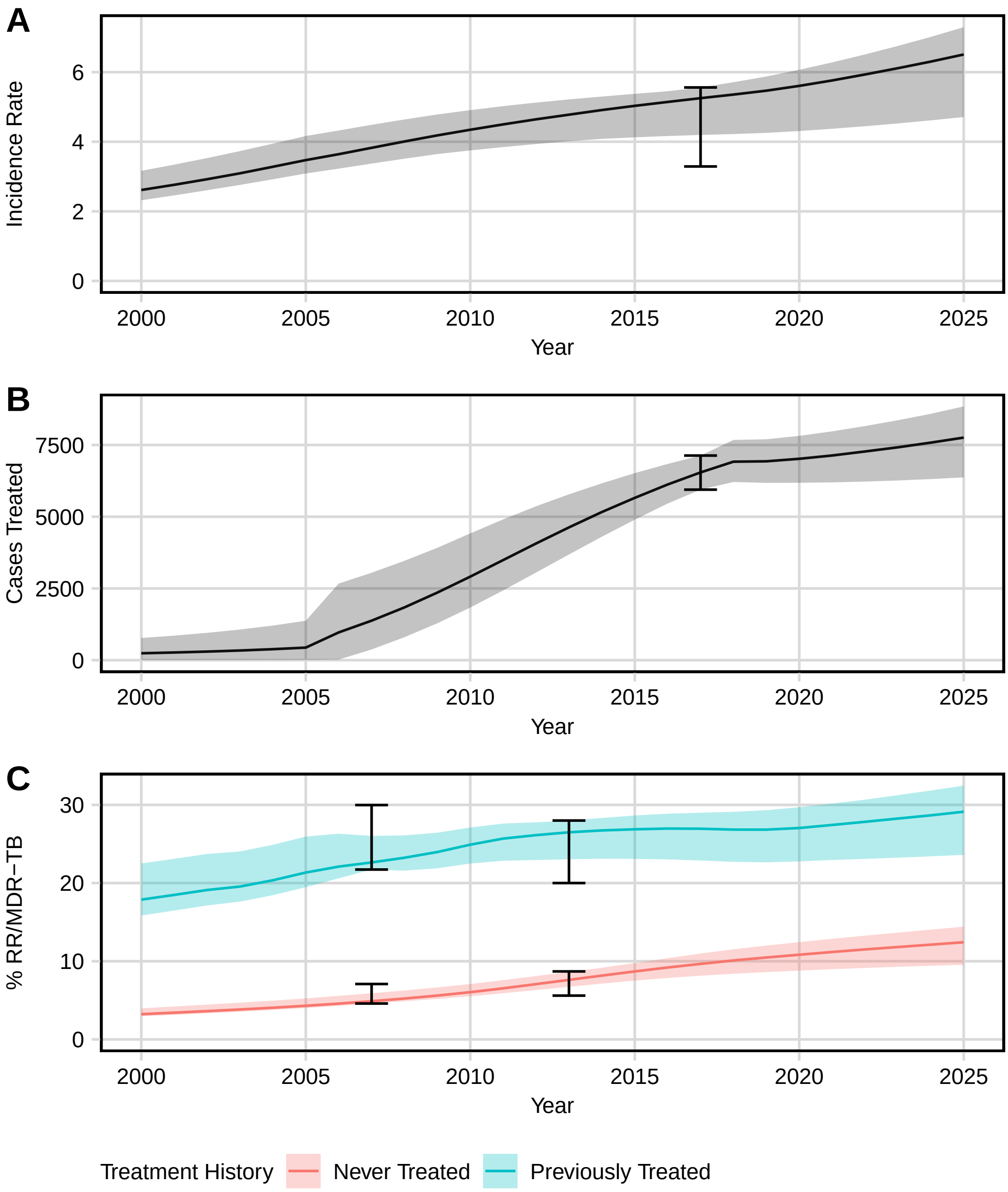

Figure S13: Calibration Results—RR/MDR-TB in China. **A**: RR/MDR-TB incidence rate. **B**: Number RR/MDR-TB treatments in the Chinese Center for Disease Control and Prevention system. **C**: % of RR/MDR-TB among all TB case notifications, disaggregated by previous treatment for tuberculosis. Line represents median trajectory; ribbons represent minimum and maximum trajectories and error bars represent calibration targets.

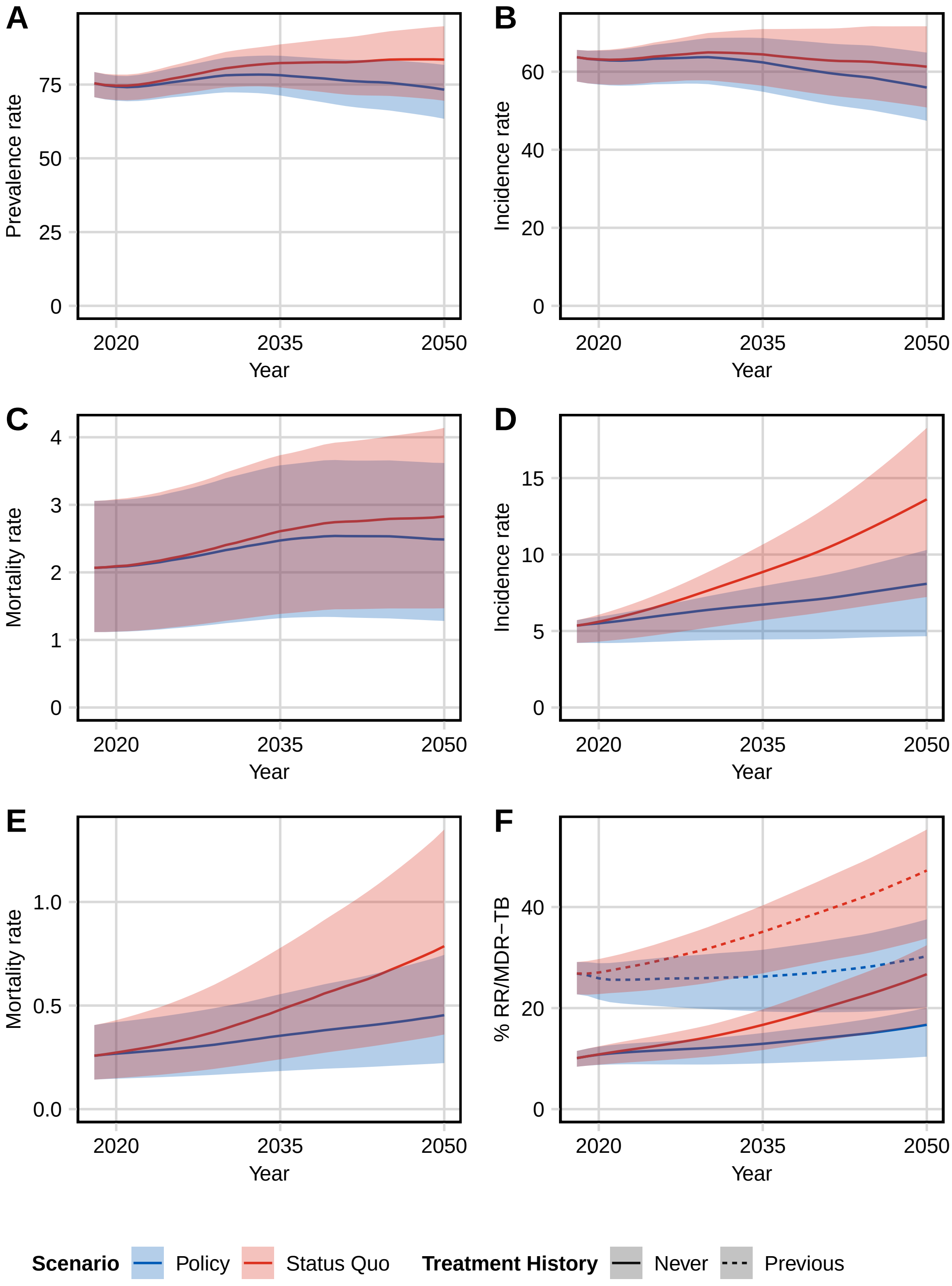

Figure S14: Baseline (no vaccine) projections–China. **A:** All TB Prevalence rate. **B**: All TB Incidence rate. **C**: All TB mortality rate. **D**: RR/MDR-TB incidence rate. **E**: RR/MDR-TB mortality rate. **F**: Proportion RR/MDR-TB cases among notifications, stratified by treatment history. All rates are expressed per 100,000 population.

7 Vaccine Impact

7.1 Vaccine Efficacy and Duration of Protection

Following their introduction in 2027, across both baseline scenarios and over all endpoints—incidence and mortality rate reductions, cases and deaths averted and averted RR/MDR-TB treatment—we found increasing duration of protection and vaccine efficacy led to greater vaccine impact by 2050. As expected, by 2030 both 5-year and 10-year duration of protection vaccines had identical impact as no waning of vaccination had occurred in any cohort which received the vaccine.

7.2 Host-Infection Status Required for Vaccine Efficacy

We found that pre– and post– infection (P&PI) vaccines had a greater impact than pre-infection (PRI) vaccines or post-infection (PSI) vaccines (sections 7.4 and 7.5). PRI vaccines had a comparable or lower impact than PSI vaccines on all TB in India and China and RR/MDR-TB in India. In China, the PRI effect on RR/MDR-TB was greater than PSI effect. Moreover, the impact of PSI vaccines on RR/MDR-TB was substantially higher in India than China (Table S16), whereas the impact of P&PI vaccines was comparable. In contrast, the impact of both P&PI and PSI vaccines on all TB was only modestly greater in India than China.

Two key differences between the TB epidemics in China and India contributed to these differences. First, the prevalence of latent tuberculosis infection (LTBI) (Fig S15) is substantially higher in India (∼40–50%) than China (∼10–15%). Second, the composition of incident tuberculosis in terms of *pathway to arrival at active disease*—for both overall and RR/MDR—differed substantially between China and India. We disaggregated incident TB into the following streams:

1. Active disease due to fast progression following infection (NP), which is further disaggregated into:
   (a) Active disease due to fast progression following infection of *susceptible* (NP-S)
   (b) Active disease due to fast progression following **re**-infection of *latent* or *recovered* (NP-LR)
2. Reactivation or relapse to active disease from *latent* or *recovered* populations (RR).

RR/MDR-TB RR/MDR-TB incidence in India driven by approximately equal proportions of NP-S, NP-LR and RR streams (Table S17 and Figure S16). In China, RR/MDR-TB incidence was driven by the NP-S stream, which was approximately twice the proportion of RR, with a very small contribution from NP-LR.

All Tuberculosis In India, the NP stream (the sum of NP-LR and NP-S) was the dominant component of all TB incidence, whereas in China the RR stream was the dominant component (Table S17 and Figure S16). Furthermore, following from the higher prevalence of LTBI in India, the contribution of the NP-LR stream to incidence was substantially higher (median 19.6–21.8%) than in China (median 1.5–1.9%).

Differential Vaccine Efficacy Post-infection vaccine efficacy was mediated by two separate mechanisms (1) reduced fast progression to active disease, following re-infection by *M. tuberculosis* in individuals who are in the latent or recovered states and; (2) reduced rate of reactivation and relapse from the latent and recovered states, respectively. In contrast, pre-infection vaccine efficacy was mediated by reduced fast progression following infection by *M. tuberculosis* of susceptible individuals.

In India, the proportion of RR/MDR-TB incidence "avertible" by PSI vaccine efficacy (the sum of RR + NP-LR) was considerably larger than China (Table S17). PSI vaccines reduce incidence in the NP-LR and RR streams—and therefore force of infection—more in India than China, leading to a greater estimate of vaccine impact (Table S16).

In China, a greater proportion of RR/MDR-TB incidence is "avertible" by PRI efficacy (the NP-S substream) than PSI efficacy (NP-LR + RR) (Table S17). Consequently, PRI vaccines lead to a greater reduction in RR/MDR-TB incidence than PSI vaccines. The opposite is true for all TB, where incidence avertible by PSI vaccination (NP-LR + RR) is greater than avertible by PRI vaccination (NP-S) (Table S17).

Table S16: Vaccine impact: Tuberculosis incidence rate reduction (uncertainty interval) in 2050 compared to no-vaccine “Status Quo” baseline, by 50% efficacy, 10-year pre- and post-infection (P&PI), pre-infection (PRI) and post-infection (PSI) efficacy vaccination in India and China.

		**P&PI**	**PRI**	**PSI**
**India**	RR/MDR-TB	72% (65–77)	46% (31–54)	47% (37–58)
	All TB	67% (59–71)	40% (27–47)	44% (39–49)
**China**	RR/MDR-TB	73% (66–76)	59% (49–64)	29% (27–31)
	All TB	56% (53–59)	30% (24–35)	37% (35–38)

Table S17: Incidence of TB disaggregated by origin. Values presented are *median [uncertainty interval]* proportions of the incidence streams, as a percentage, *over* 2027–2050.

**Country**	**Resistance Status**	**Incidence Stream**		
		**NP-LR**	**NP-S**	**RR**
**RR/MDR-TB**	India	28.1% [27.2–30.9]	37.7% [33.8–39.9]	34.3% [33.0–35.3]
	China	3.3% [2.8–4.0]	64.9% [63.9–67.8]	31.9% [28.2–33.3]
**All TB**	India	20.2% [19.6–21.8]	39.2% [34.4–41.6]	40.9% [39.2–44.1]
	China	1.6% [1.5–1.9]	37.1% [36.0–38.4]	61.3% [59.7–62.4]

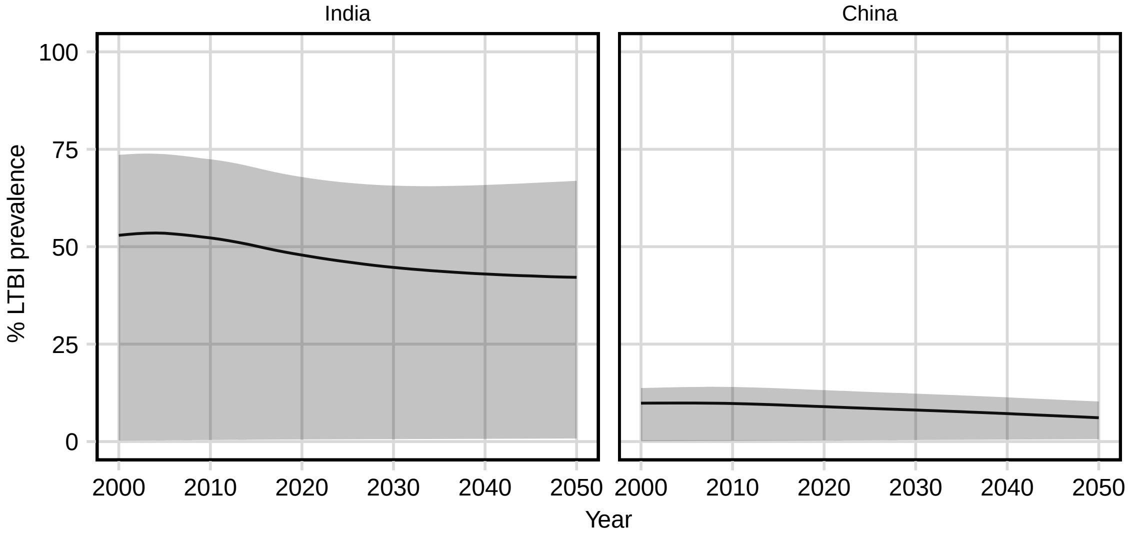

Figure S15: Latent tuberculosis infection in the baseline scenario—India & China, 2025–2050

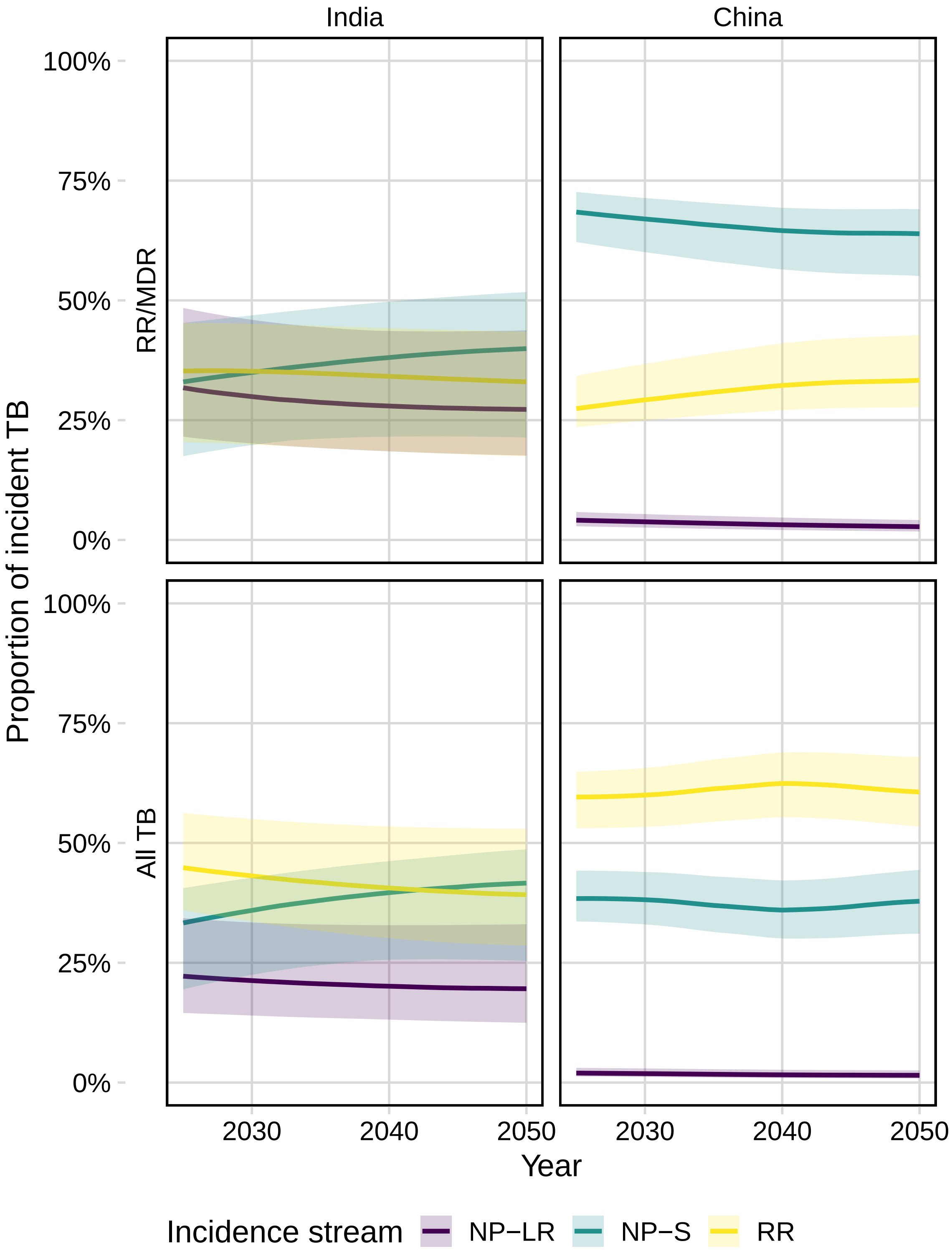

Figure S16: Incident tuberculosis, disaggregated by incidence source in the baseline scenario—India & China, 2025–2050. Lines represent median proportion. Ribbons represent uncertainty.

7.3 Scenario Analyses: Variable Mass Vaccine Campaign Coverage

Percent reduction in incidence rate in 2050 by vaccination for both RR/MDR-TB and all TB in India and China are presented in Figures S17 and S18, respectively. As expected across all vaccine types and efficacies, reduced vaccine campaign coverage leads to lower incidence rate reduction.

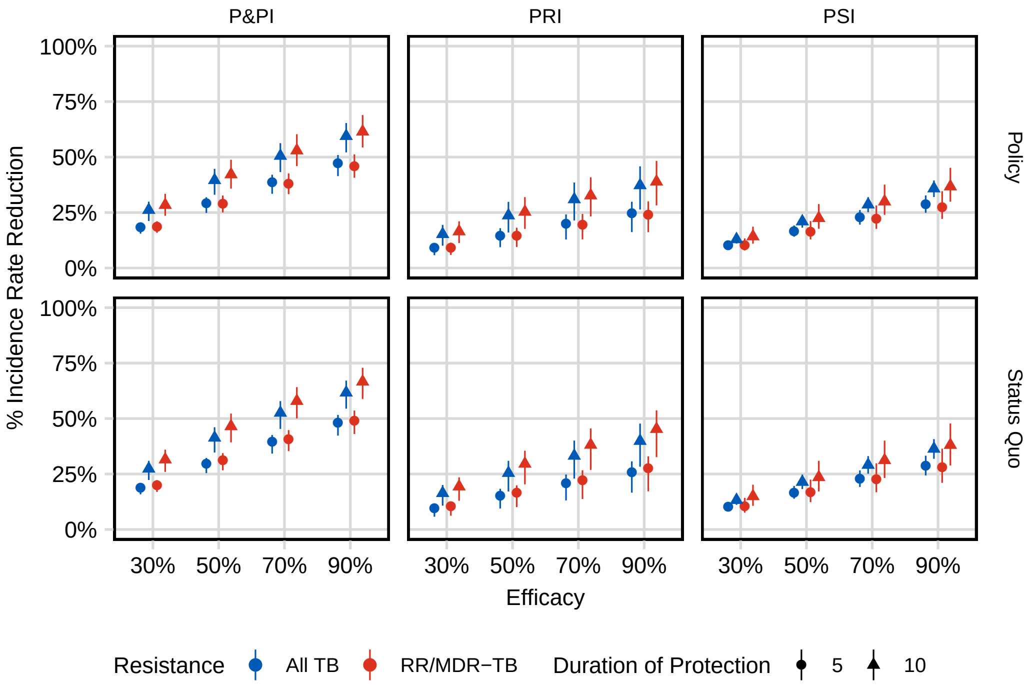

Figure S17: Percent Incidence Rate Reduction in India in 2050 by vaccination, where mass vaccination of ages 10 and above at 30% coverage and routine vaccination of 9-year olds at 80% coverage. Points represent median value. Bars represent the uncertainty interval. P&PI: pre and post-infection efficacy; PSI: post-infection efficacy; PRI: pre-infection efficacy. Horizontal facets represent drug resistance status. Vaccine provides 10-years of protection.

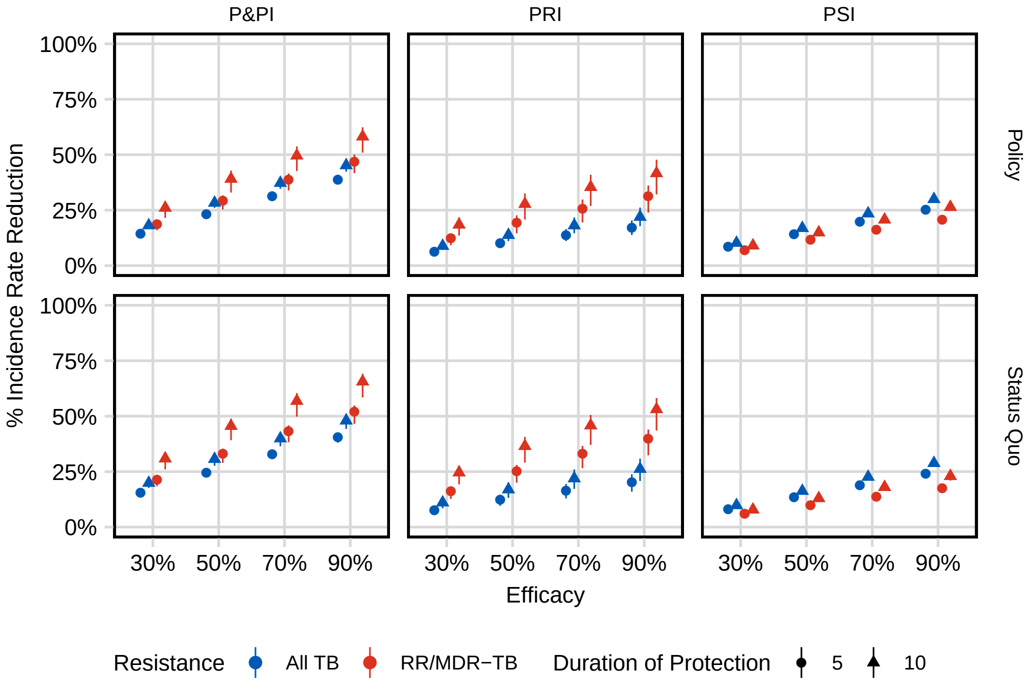

Figure S18: Percent Incidence Rate Reduction in China in 2050 by vaccination, where mass vaccination of ages 10 and above at 30% coverage and routine vaccination of 9-year olds at 80% coverage. Points represent median value. Bars represent the uncertainty interval. P&PI: pre and post-infection efficacy; PSI: post-infection efficacy; PRI: pre-infection efficacy. Horizontal facets represent drug resistance status. Vaccine provides 10-years of protection.

7.4 Scenario Analyses: Tuberculosis Incidence and Mortality Rate Reduction

Percent reduction in incidence rate and mortality rate in 2030 and 2050 by vaccination for both RR/MDR-TB and all TB in India are presented in Figures S19 and S20, respectively. Percent reduction in incidence rate and mortality rate in 2030 and 2050 by vaccination for both RR/MDR-TB and all TB in China are presented in Figures S21 and S22, respectively.

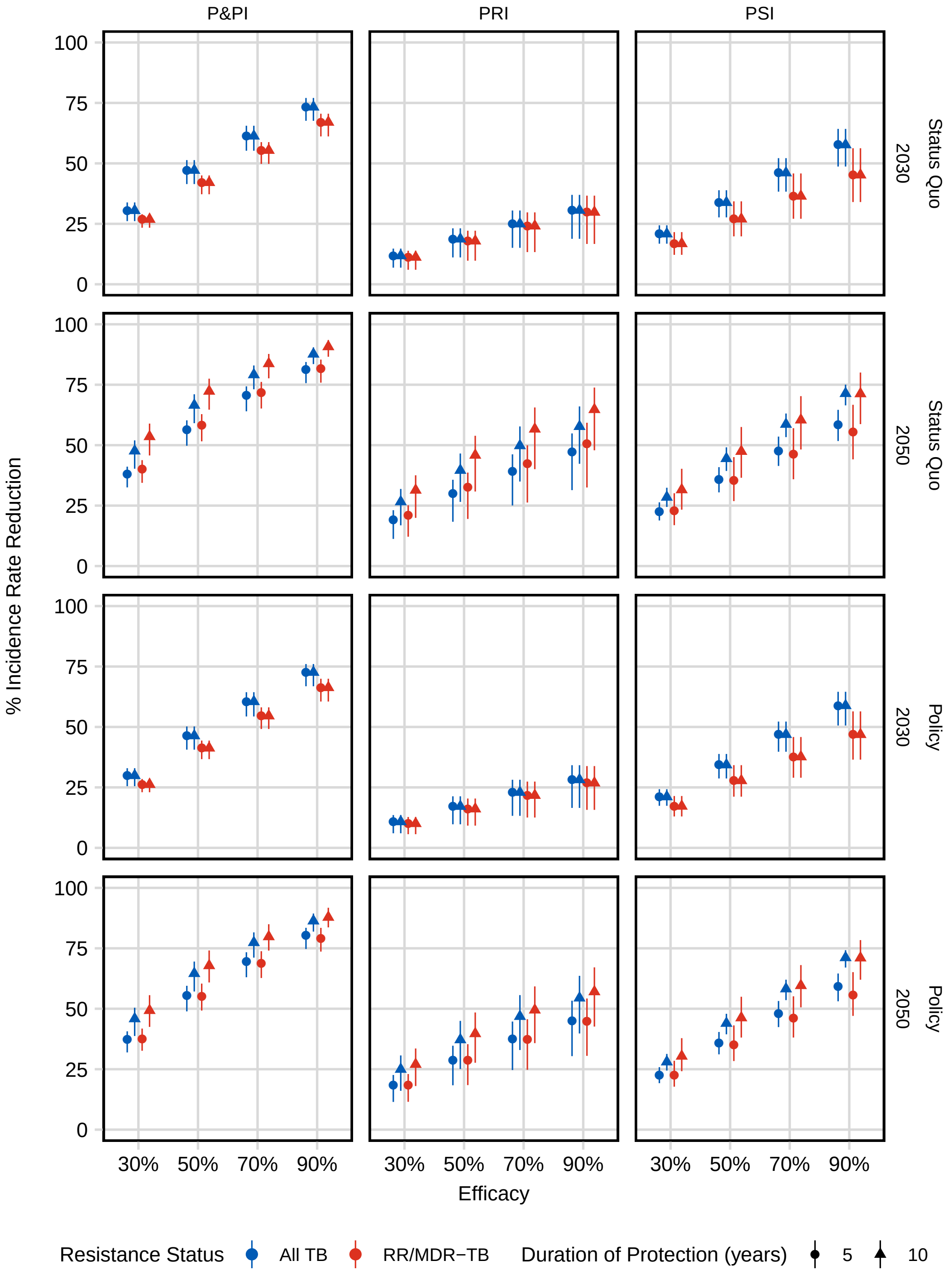

Figure S19: Percent Incidence Rate Reduction in India by vaccination. Colors represent drug-resistance status. Points represent median value. Bars represent the uncertainty interval. P&PI: pre and post-infection efficacy; PSI: post-infection efficacy; PRI: pre-infection efficacy. Horizontal facets show baseline scenarios.

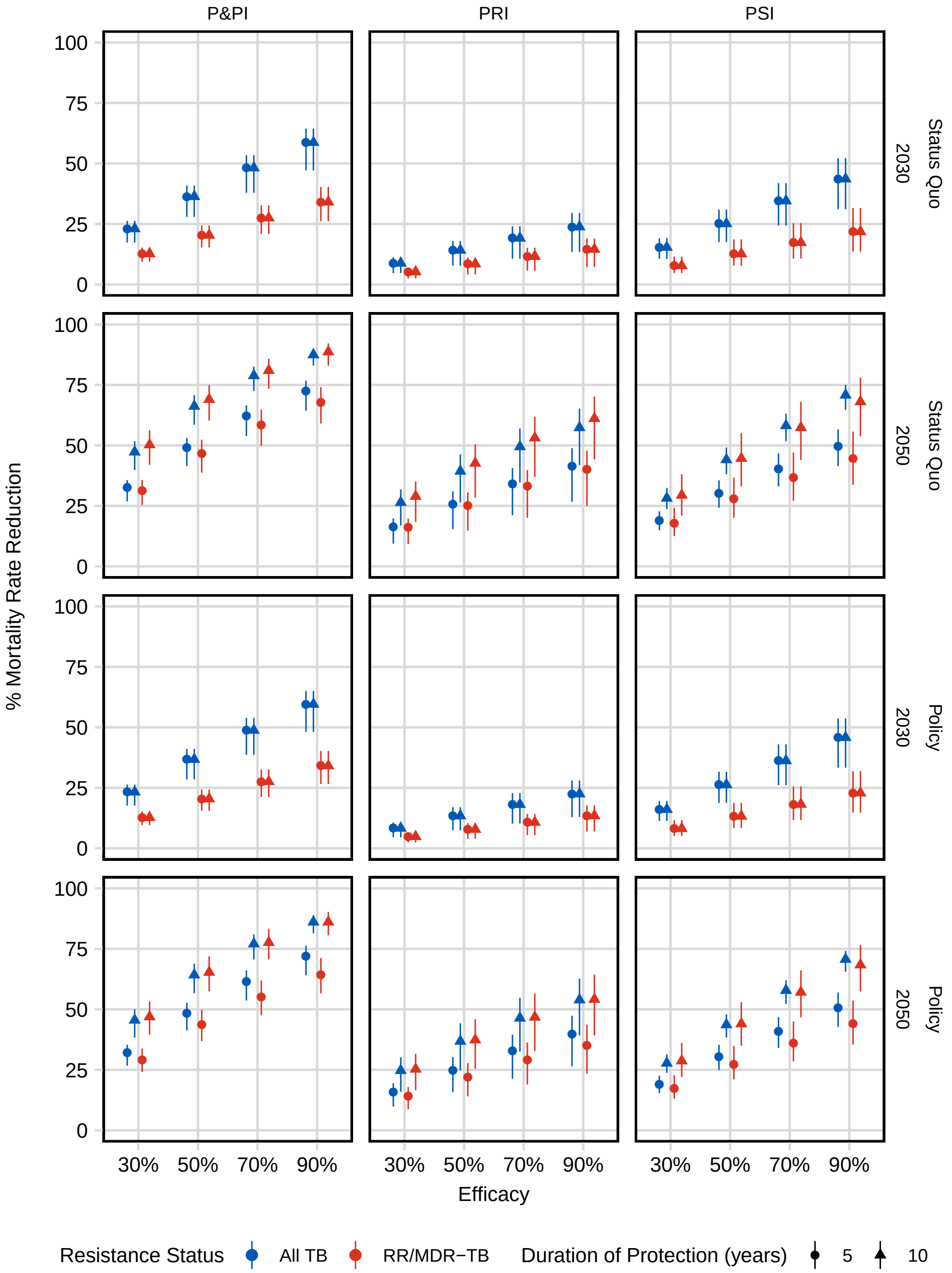

Figure S20: Percent Mortality Rate Reduction in India by vaccination. Colors represent drug-resistance status. Points represent median value. Bars represent the uncertainty interval. P&PI: pre and post-infection efficacy; PSI: post-infection efficacy; PRI: pre-infection efficacy. Horizontal facets show baseline scenarios.

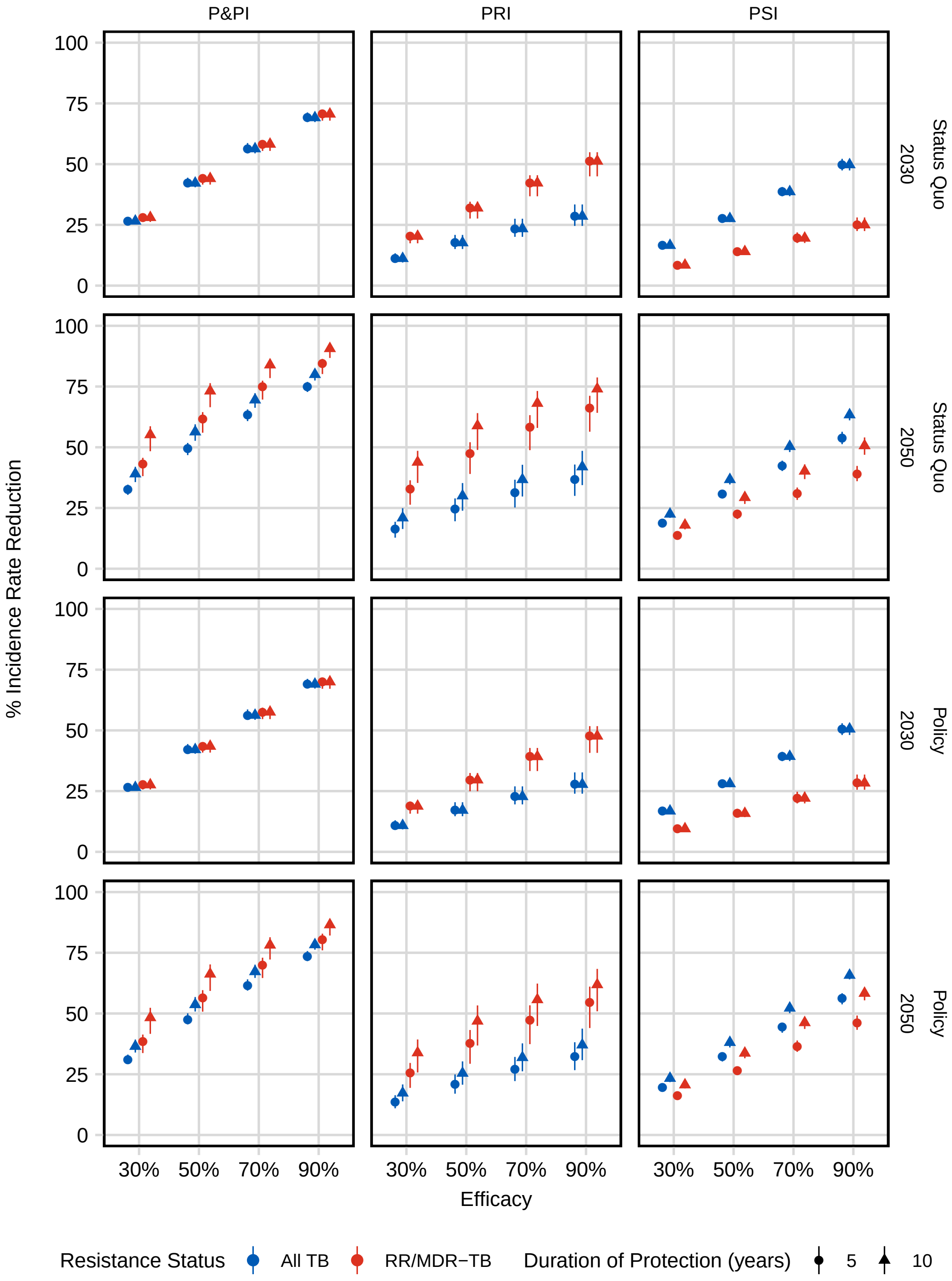

Figure S21: Percent Incidence Rate Reduction in China by vaccination. Colors represent drug-resistance status. Points represent median value. Bars represent the uncertainty interval. P&PI: pre and post-infection efficacy; PSI: post-infection efficacy; PRI: pre-infection efficacy. Horizontal facets show baseline scenarios.

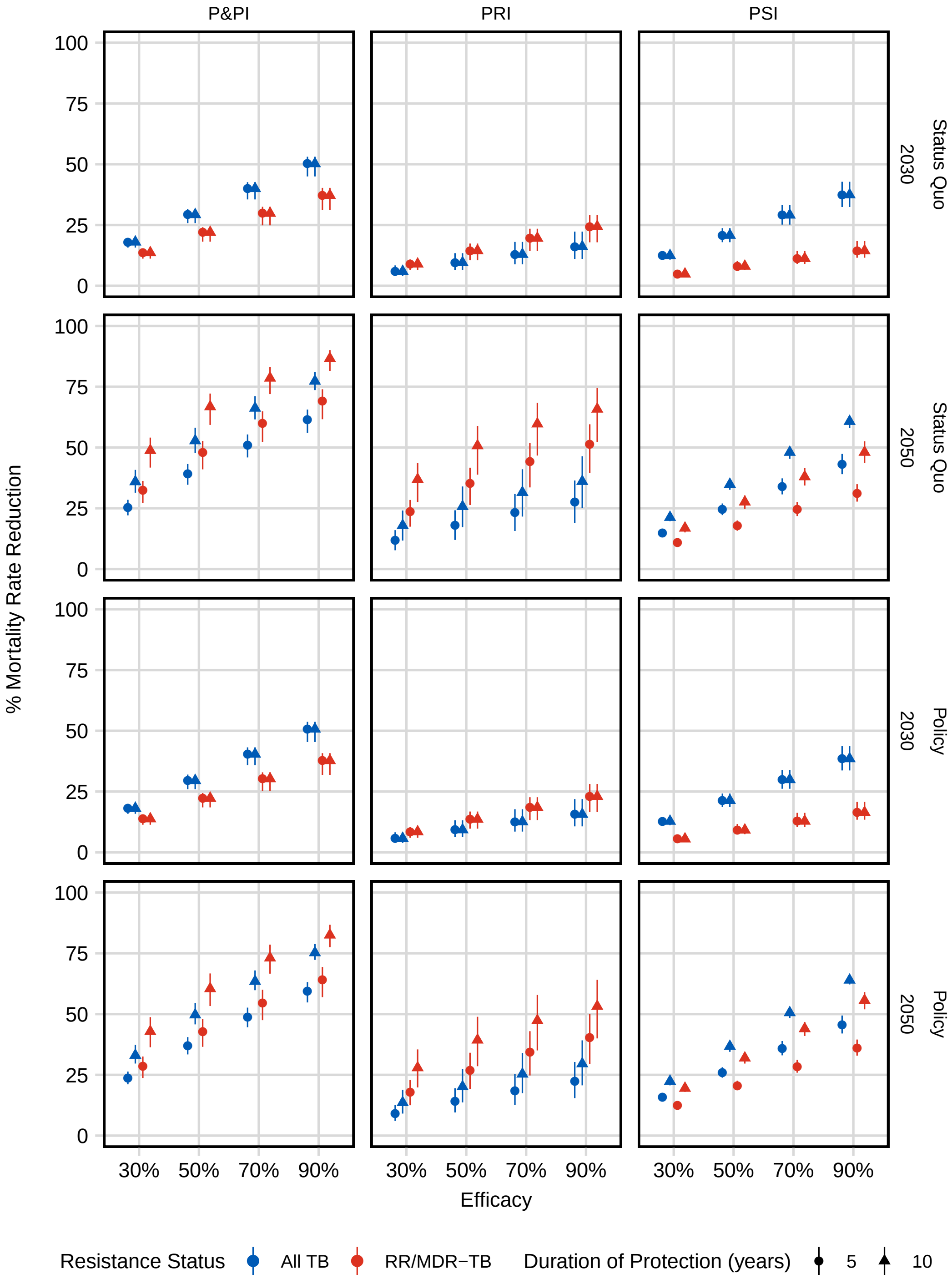

Figure S22: Percent Mortality Rate Reduction in China by vaccination. Colors represent drug-resistance status. Points represent median value. Bars represent the uncertainty interval. P&PI: pre and post-infection efficacy; PSI: post-infection efficacy; PRI: pre-infection efficacy. Horizontal facets show baseline scenarios.

7.5 Scenario Analyses: Tuberculosis Cases and Deaths Averted

The number of averted RR/MDR-TB cases and deaths averted by vaccination by 2030 and 2050 in India are presented in Figures S23 and S24. The number of averted all TB cases and deaths averted by vaccination by 2030 and 2050 in India are presented in Figures S25 and S26.

The number of averted RR/MDR-TB cases and deaths averted by vaccination by 2030 and 2050 in China are presented in Figures S27 and S28. The number of averted all TB cases and deaths averted by vaccination by 2030 and 2050 in China are presented in Figures S29 and S30.

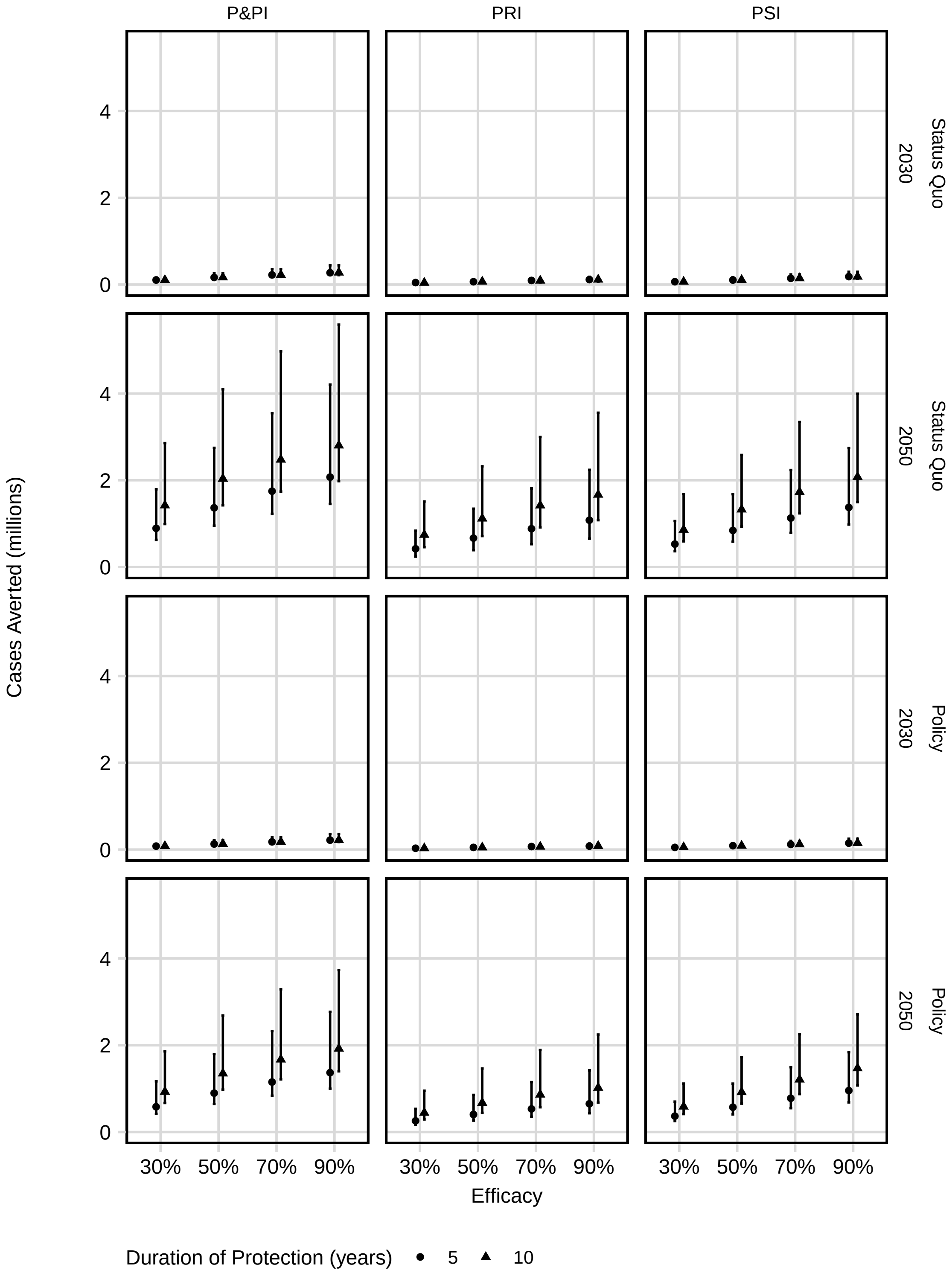

Figure S23: RR/MDR-TB Cases Averted in India by vaccination by 2030 and 2050. Points represent median value. Bars represent the uncertainty interval. P&PI - pre and post-infection efficacy; PSI - post-infection efficacy; PRI - pre-infection efficacy. Horizontal facets show baseline scenarios.

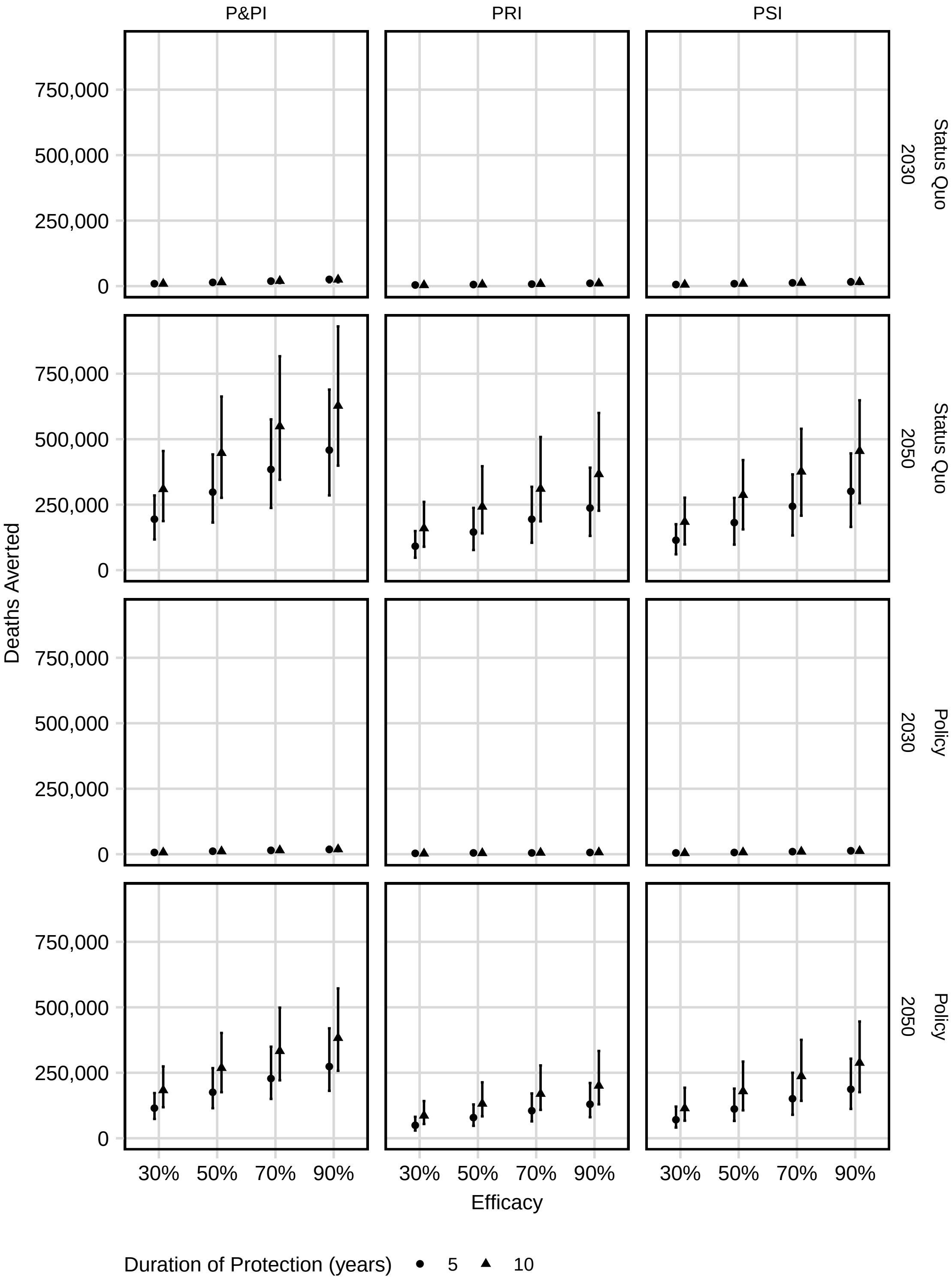

Figure S24: RR/MDR-TB Deaths Averted in India by vaccination by 2030 and 2050. Points represent median value. Bars represent the uncertainty interval. P&PI - pre and post-infection efficacy; PSI - post-infection efficacy; PRI - pre-infection efficacy. Horizontal facets show baseline scenarios.

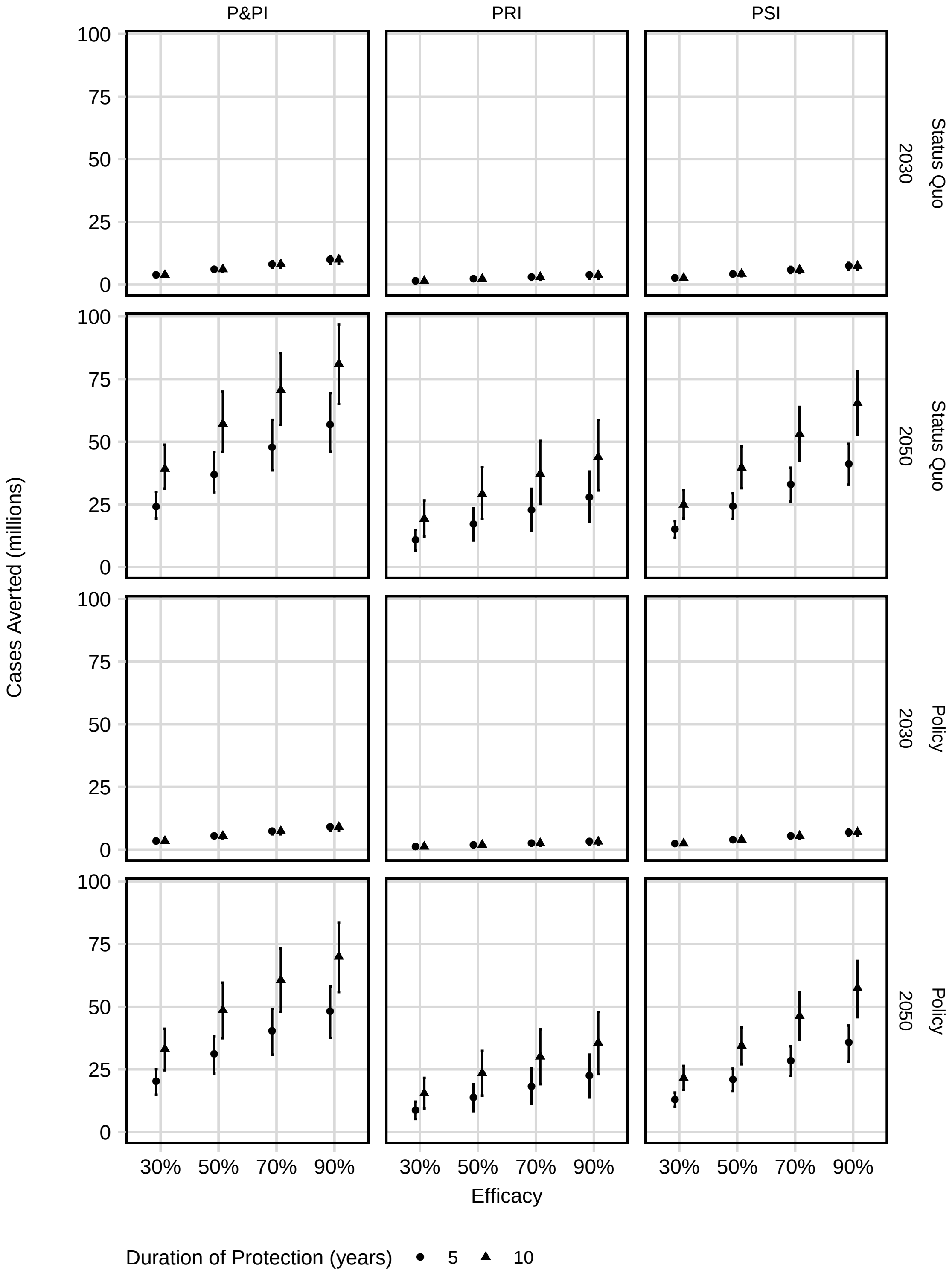

Figure S25: All TB Cases Averted in India by vaccination by 2030 and 2050. Points represent median value. Bars represent the uncertainty interval. P&PI - pre and post-infection efficacy; PSI - post-infection efficacy; PRI - pre-infection efficacy. Horizontal facets show baseline scenarios.

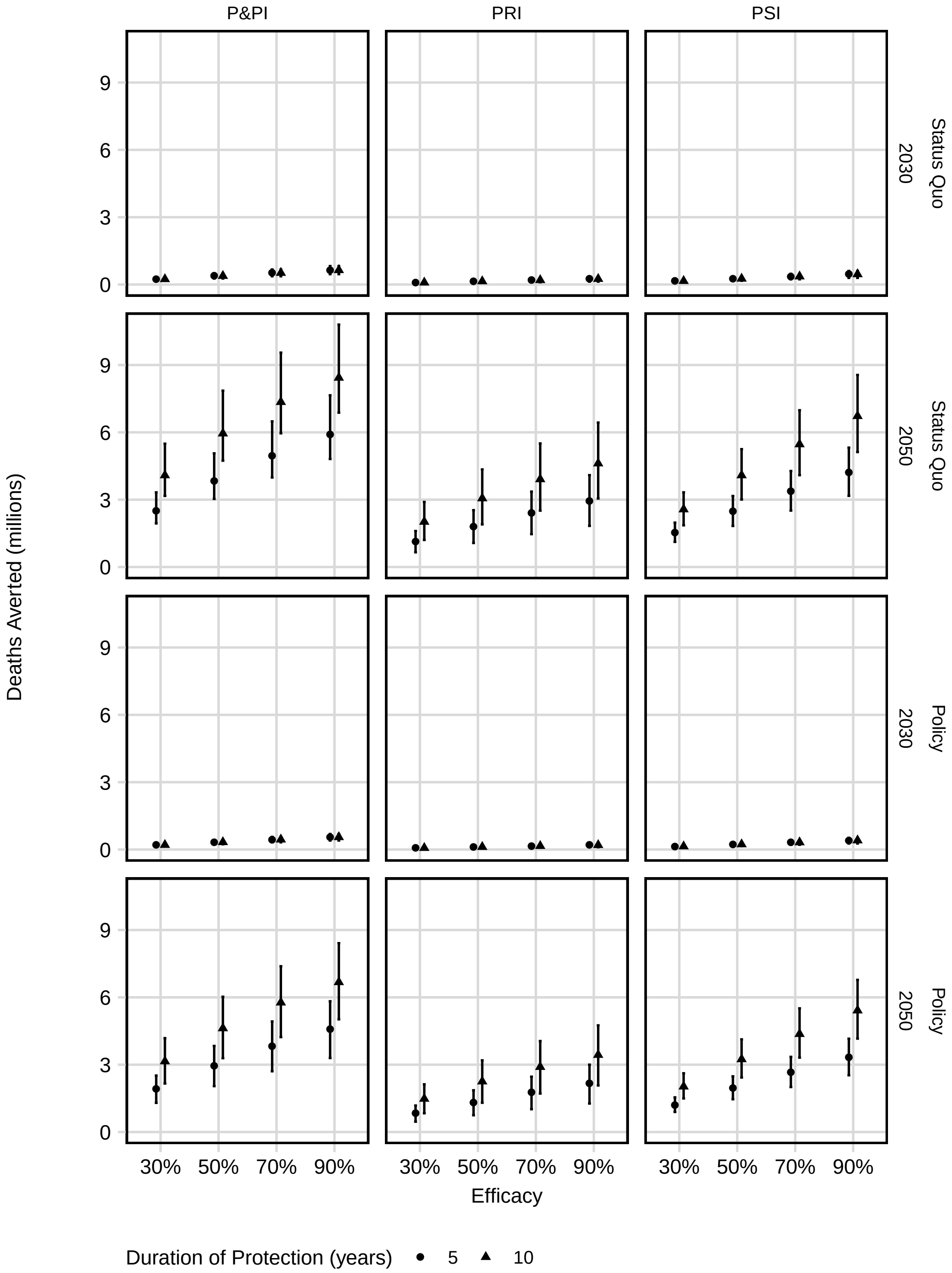

Figure S26: All TB Deaths Averted in India by vaccination by 2030 and 2050. Points represent median value. Bars represent the uncertainty interval. P&PI - pre and post-infection efficacy; PSI - post-infection efficacy; PRI - pre-infection efficacy. Horizontal facets show baseline scenarios.

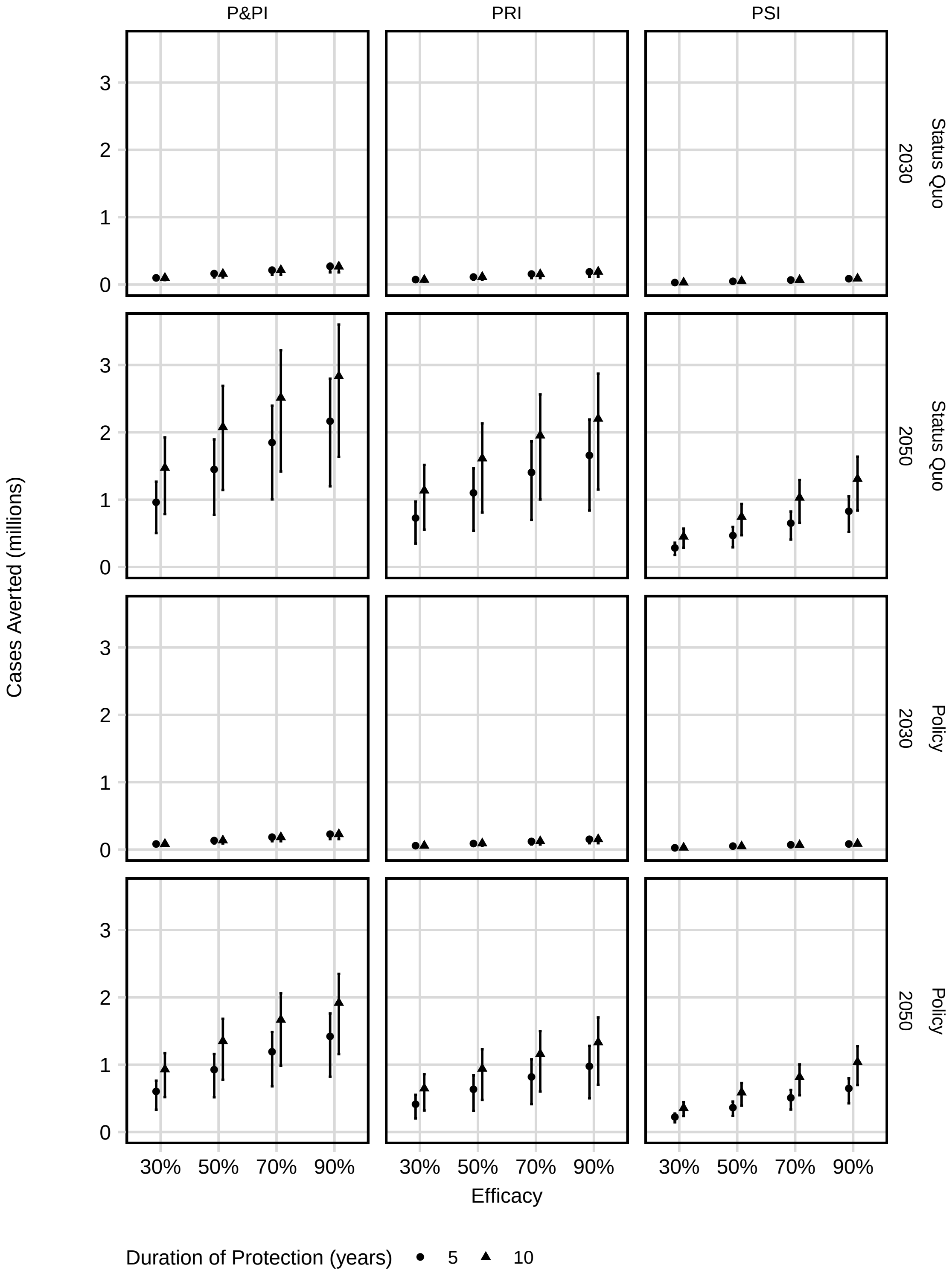

Figure S27: RR/MDR-TB Cases Averted in China by vaccination by 2030 and 2050. Points represent median value. Bars represent the uncertainty interval. P&PI - pre and post-infection efficacy; PSI - post-infection efficacy; PRI - pre-infection efficacy. Horizontal facets show baseline scenarios.

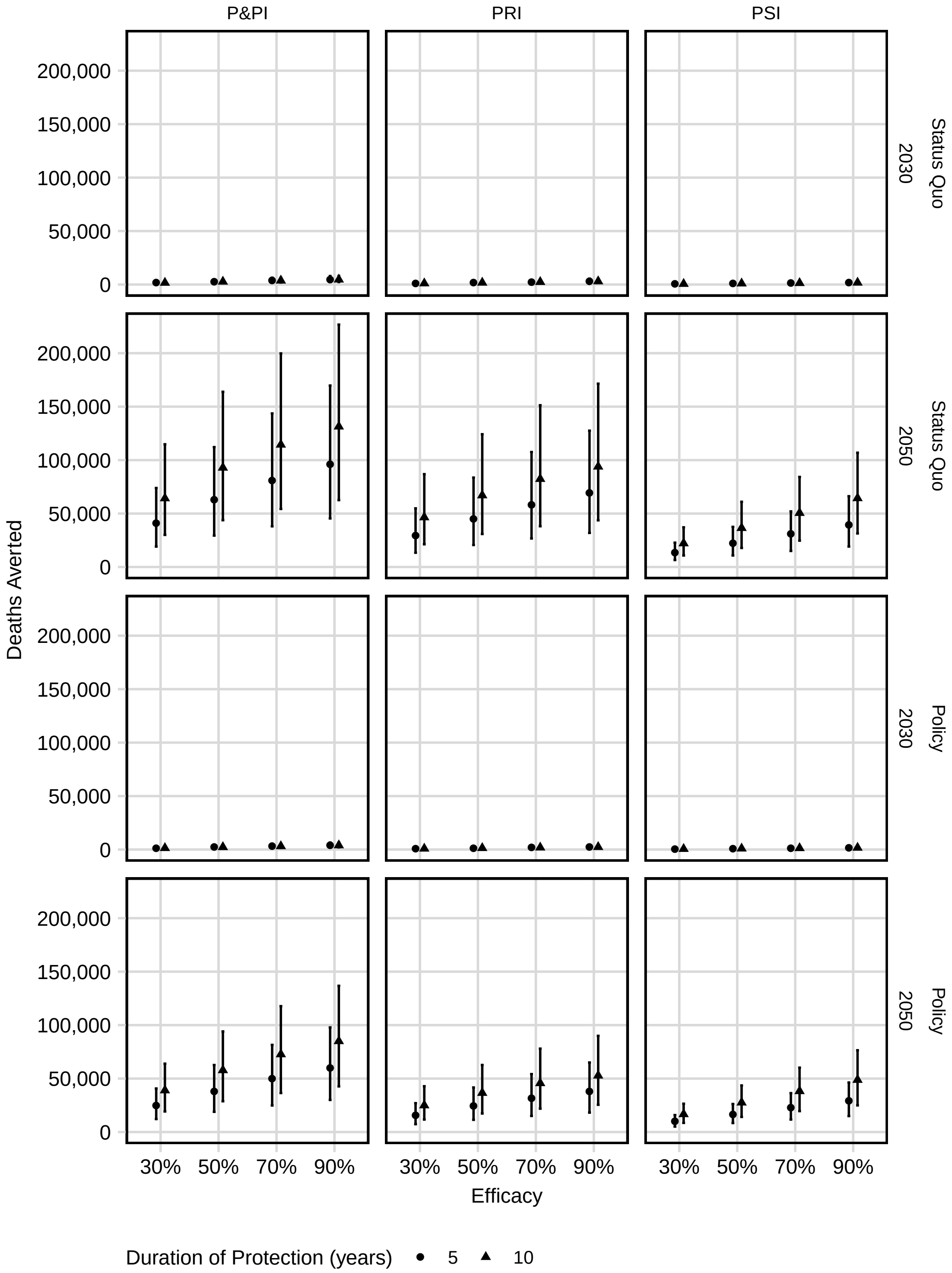

Figure S28: RR/MDR-TB Deaths Averted in China by vaccination by 2030 and 2050. Points represent median value. Bars represent the uncertainty interval. P&PI - pre and post-infection efficacy; PSI - post-infection efficacy; PRI - pre-infection efficacy. Horizontal facets show baseline scenarios.

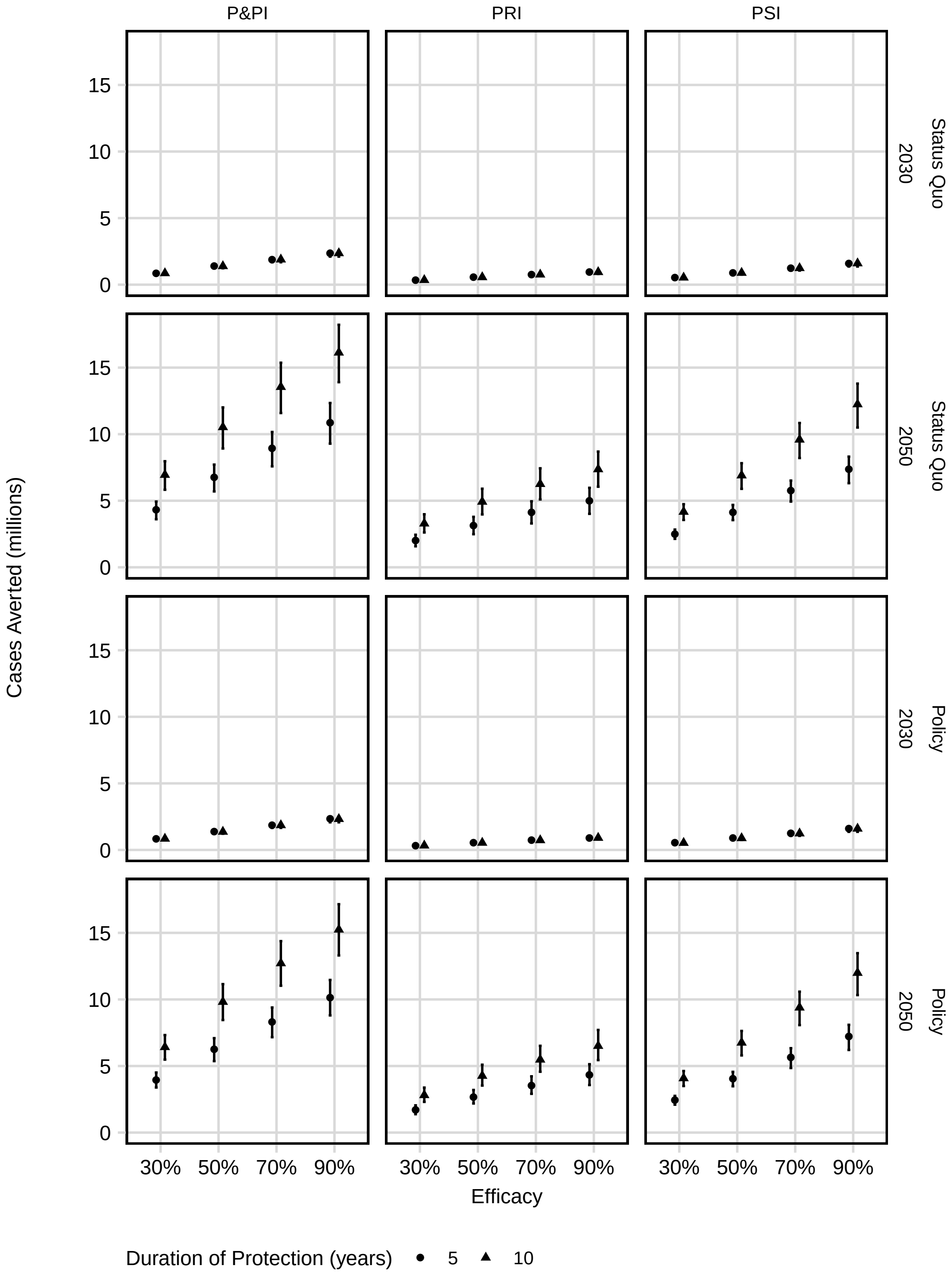

Figure S29: All TB Cases Averted in China by vaccination by 2030 and 2050. Points represent median value. Bars represent the uncertainty interval. P&PI - pre and post-infection efficacy; PSI - post-infection efficacy; PRI - pre-infection efficacy. Horizontal facets show baseline scenarios.

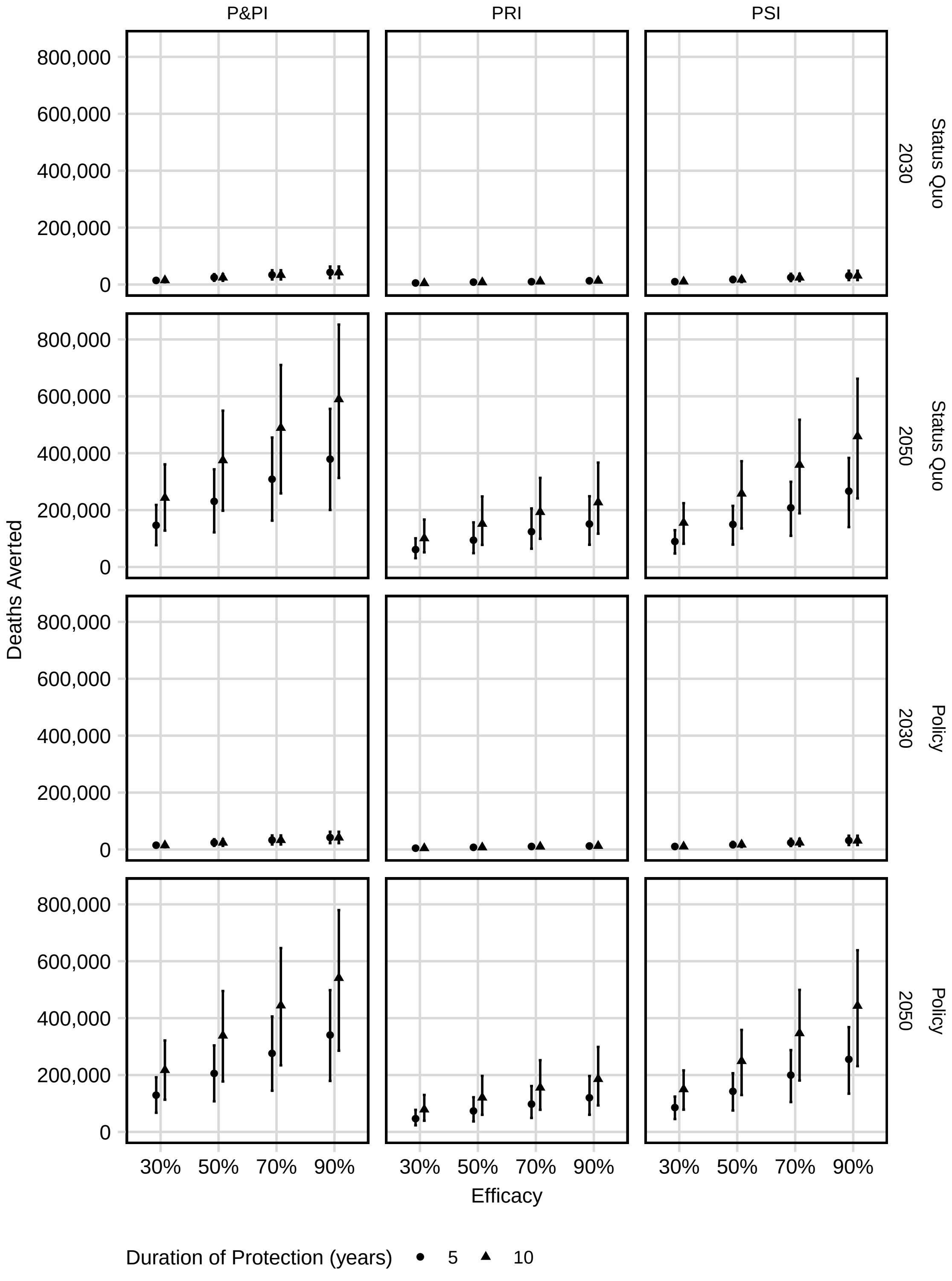

Figure S30: All TB Deaths Averted in China by vaccination by 2030 and 2050. Points represent median value. Bars represent the uncertainty interval. P&PI - pre and post-infection efficacy; PSI - post-infection efficacy; PRI - pre-infection efficacy. Horizontal facets show baseline scenarios.

7.6 Scenario Analyses: Averted Anti-tuberculosis Therapy

The number of averted RR/MDR-TB and DS-TB treatment regimens averted by 2030 and 2050 by vaccination in India is presented in Figures S31 and S32. The number of averted RR/MDR-TB and DS-TB treatment regimens averted by 2030 and 2050 by vaccination in China is presented in Figures S33 and S34.

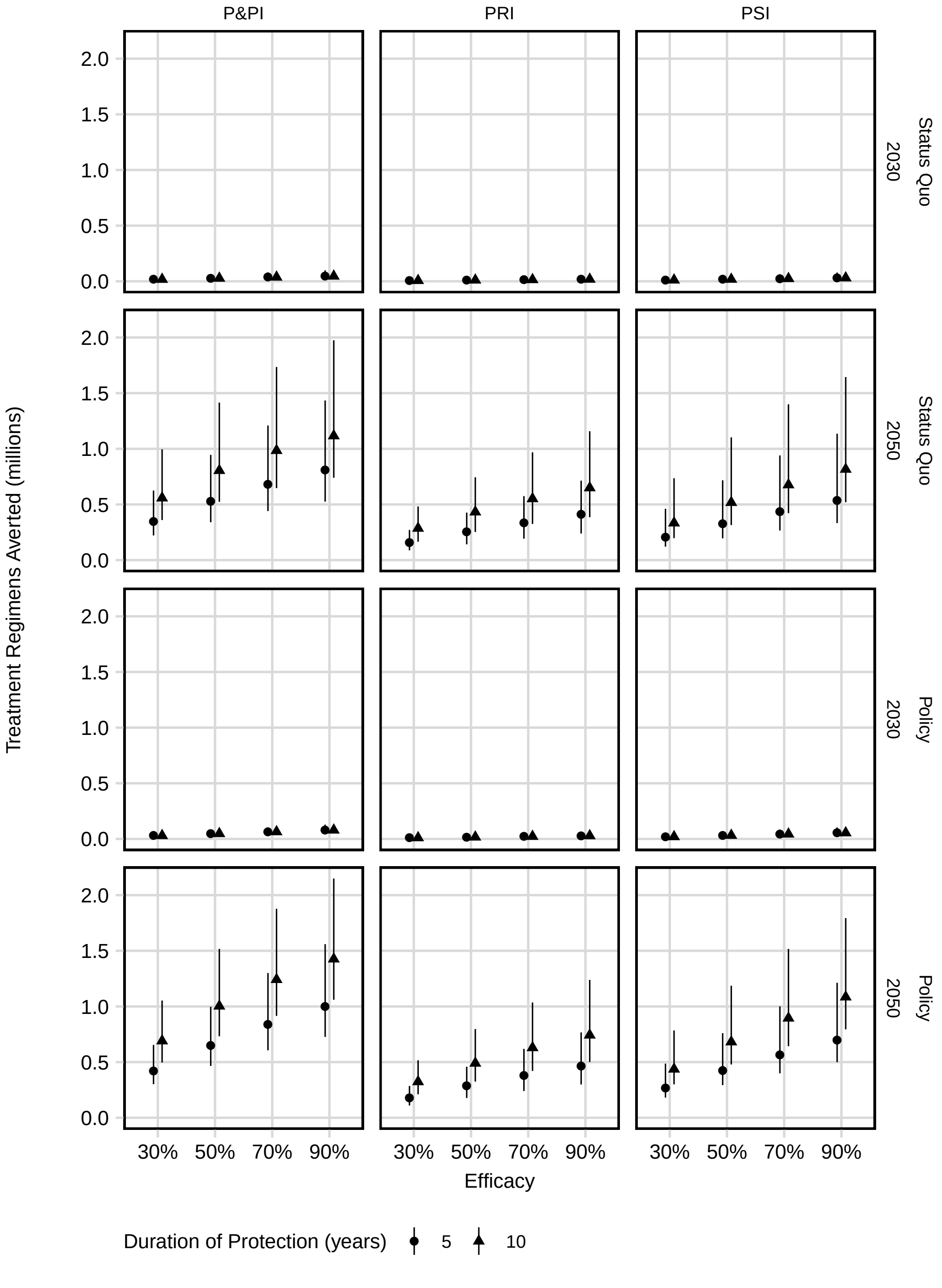

Figure S31: RR/MDR-TB Treatment Regimens Averted by 2030 & 2050 in India. Points represent median value. Bars represent the uncertainty interval. P&PI - pre and post-infection efficacy; PSI - post-infection efficacy; PRI - pre-infection efficacy. Horizontal facets show baseline scenarios.

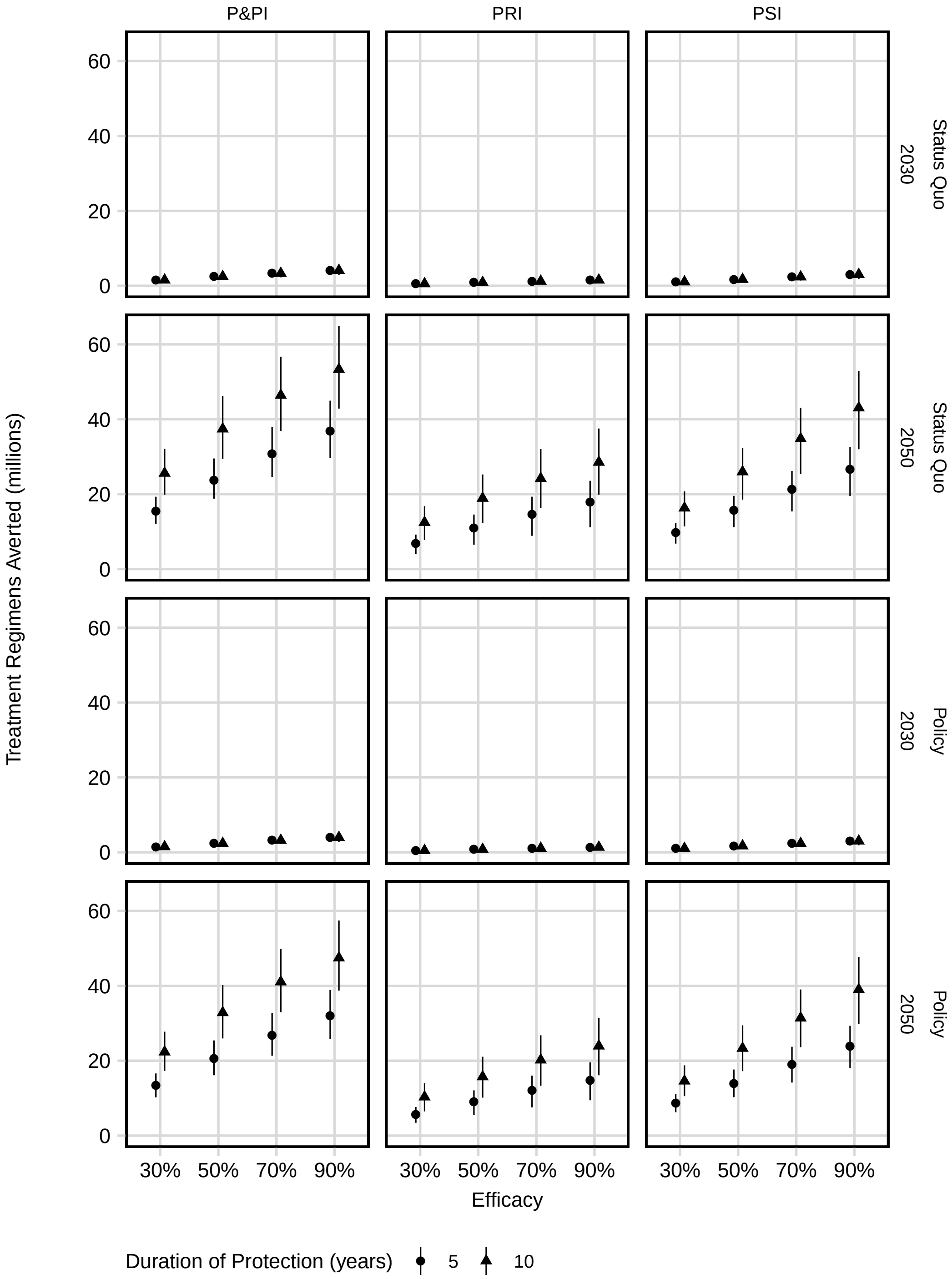

Figure S32: DS-TB Treatment Regimens Averted by 2030 & 2050 in India. Points represent median value. Bars represent the uncertainty interval. P&PI - pre and post-infection efficacy; PSI - post-infection efficacy; PRI - pre-infection efficacy. Horizontal facets show baseline scenarios.

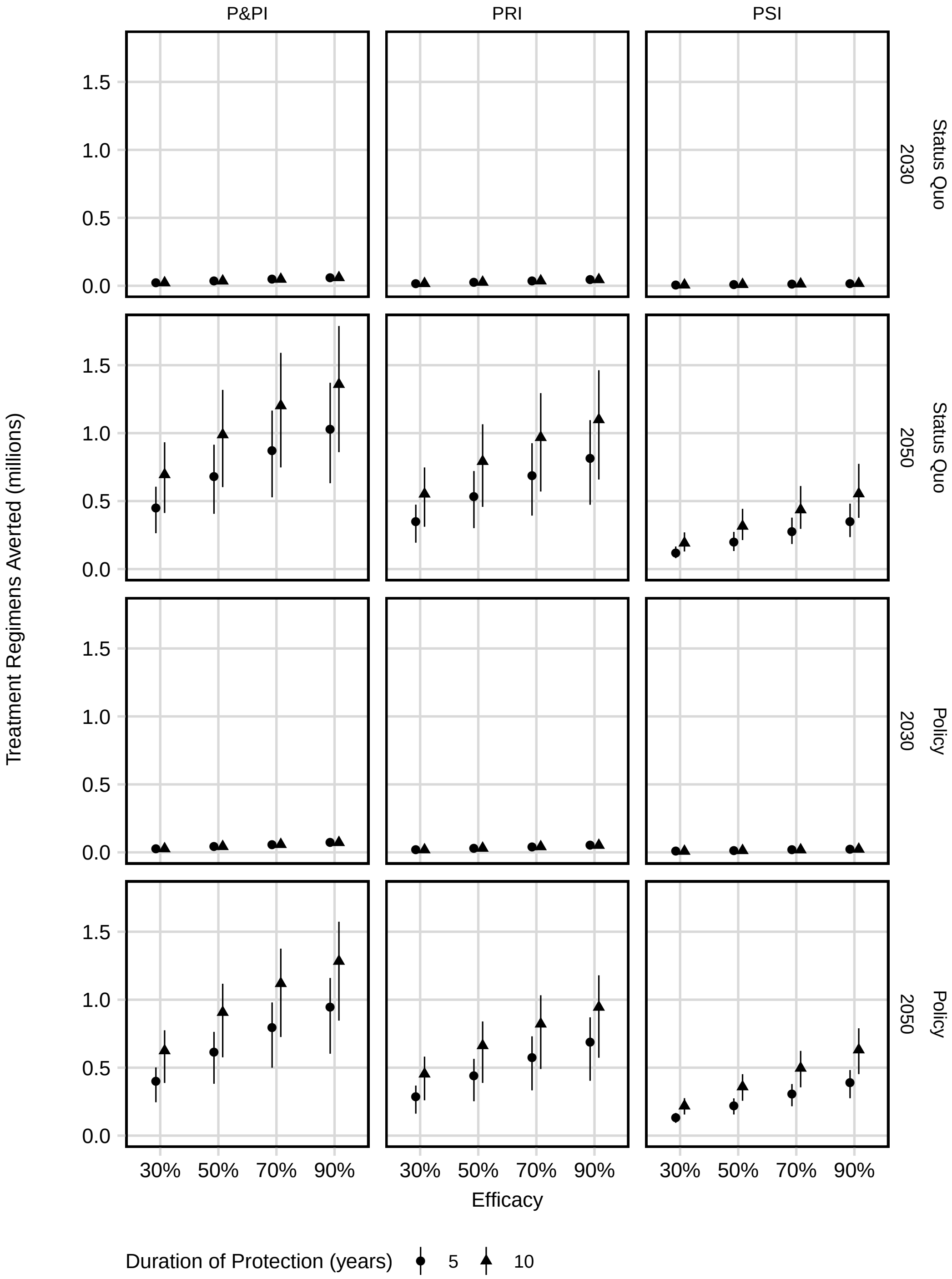

Figure S33: RR/MDR-TB Treatment Regimens Averted by 2030 & 2050 in China. Points represent median value. Bars represent the uncertainty interval. P&PI - pre and post-infection efficacy; PSI - post-infection efficacy; PRI - pre-infection efficacy. Horizontal facets show baseline scenarios.

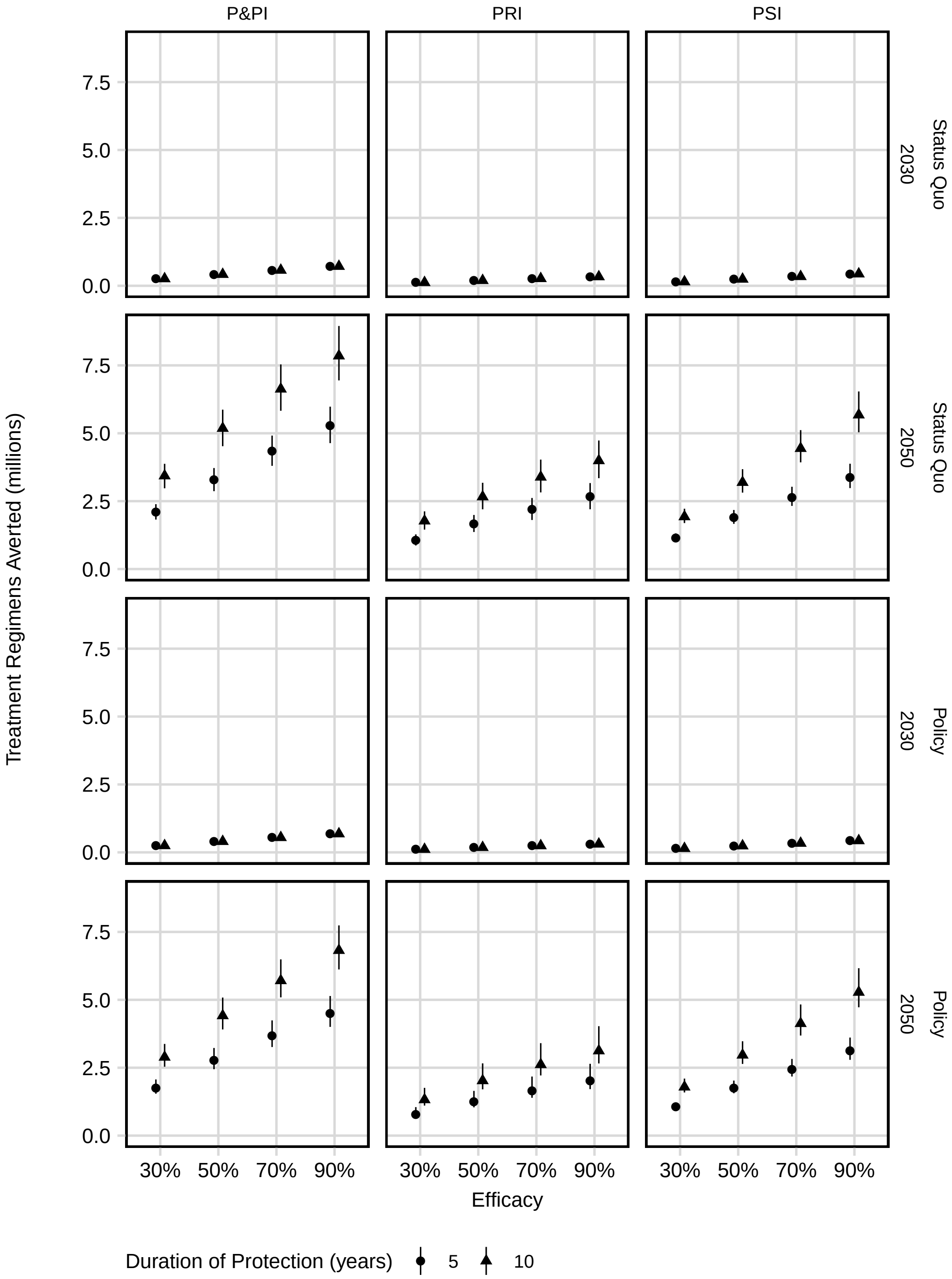

Figure S34: DS-TB Treatment Regimens Averted by 2030 & 2050 in China. Points represent median value. Bars represent the uncertainty interval. P&PI - pre and post-infection efficacy; PSI - post-infection efficacy; PRI - pre-infection efficacy. Horizontal facets show baseline scenarios.

8 Cost-effectiveness

8.1 Incremental Cost Effectiveness Ratios

Estimated incremental cost-effectiveness ratios for USD 10 and USD 30 vaccines are presented in Figures S35 and S36 for India and Figures S37 and S38 for China, respectively.

DALYs averted and costs are affected differently in the discounted analyses as benefits of vaccination are realised later compared to costs, which are incurred earlier. Consequently, we find that ICERs in discounted analyses to be greater than undiscounted analyses across both countries and over the range of vaccine characteristics. Further, in both countries, we found that ICERs for vaccination in the “Policy” scenario to be greater than in the “Status Quo” scenario, consistent with the lower absolute averted burden of disease by vaccination (section 7.5).

Estimated incremental cost-effectiveness ratios for USD 10 vaccines, delivered with routine vaccine coverage of 80% but mass campaign coverage of 30% for India and China are presented in Figures S39 and S40 respectively.

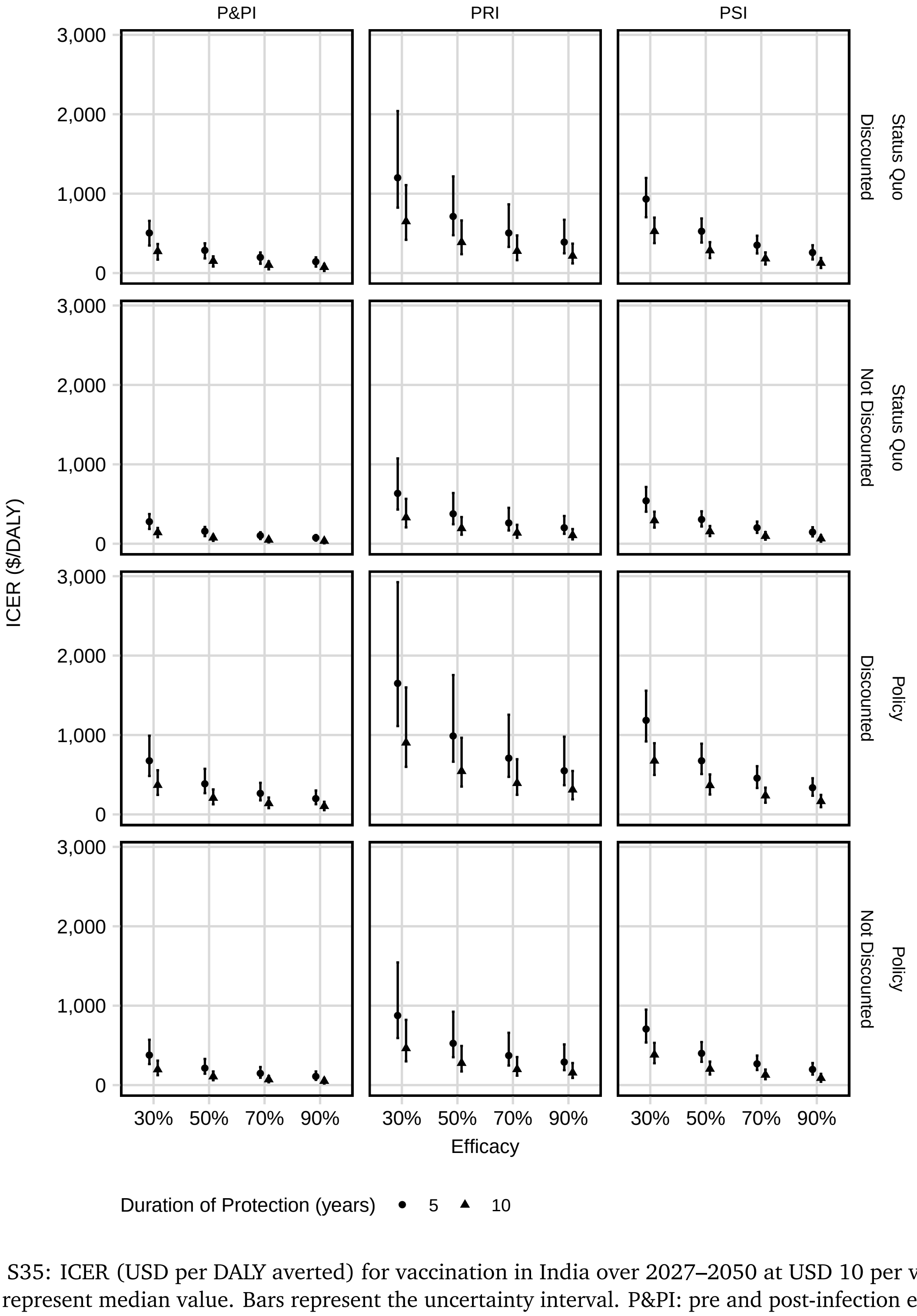

Figure S35: ICER (USD per DALY averted) for vaccination in India over 2027–2050 at USD 10 per vaccine. Points represent median value. Bars represent the uncertainty interval. P&PI: pre and post-infection efficacy; PSI: post-infection efficacy; PRI: pre-infection efficacy. Horizontal facets show baseline scenarios.

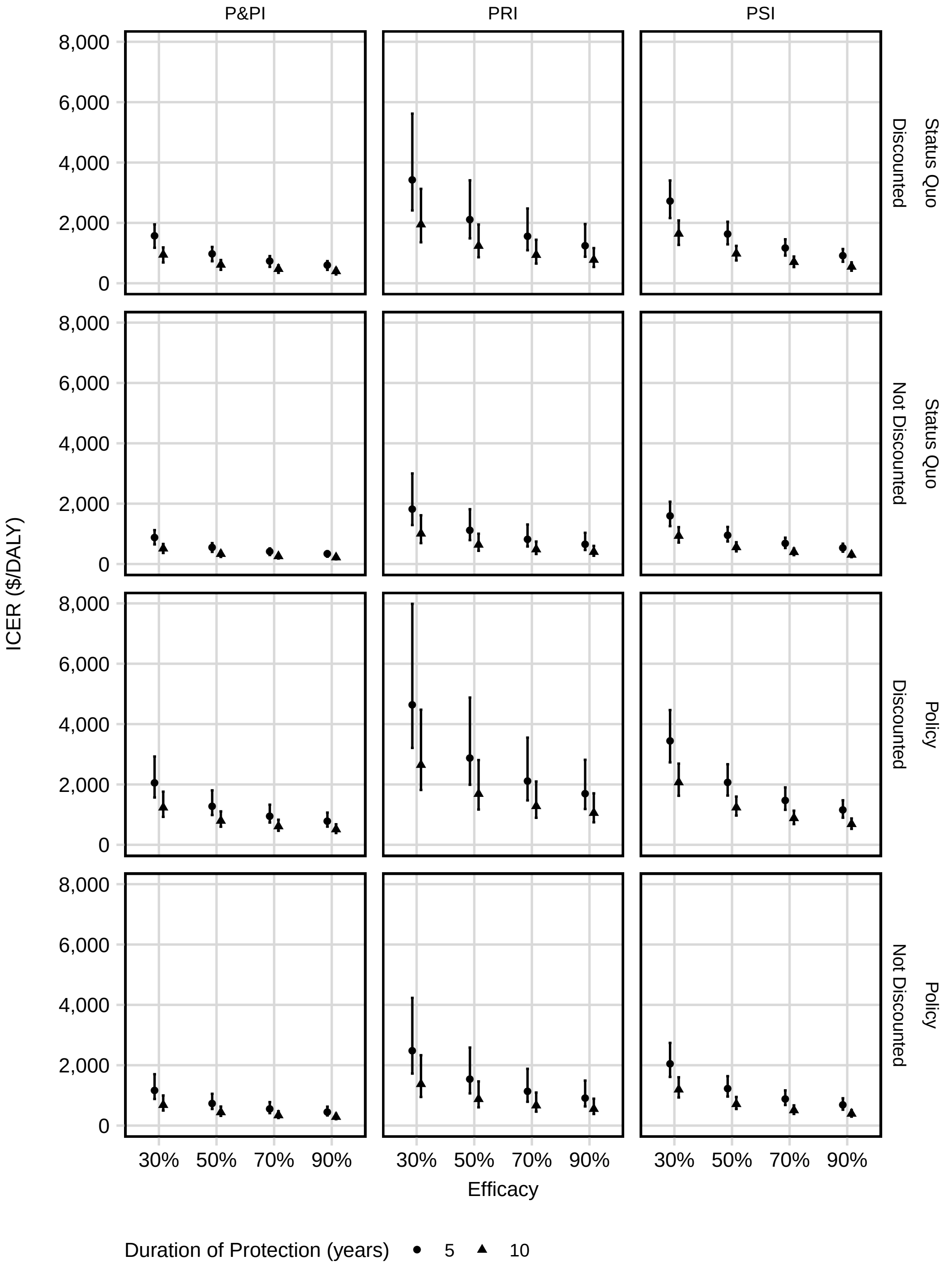

Figure S36: ICER (USD per DALY averted) for vaccination in India over 2027–2050 at USD 30 per vaccine. Points represent median value. Bars represent the uncertainty interval. P&PI: pre and post-infection efficacy; PSI: post-infection efficacy; PRI: pre-infection efficacy. Horizontal facets show baseline scenarios.

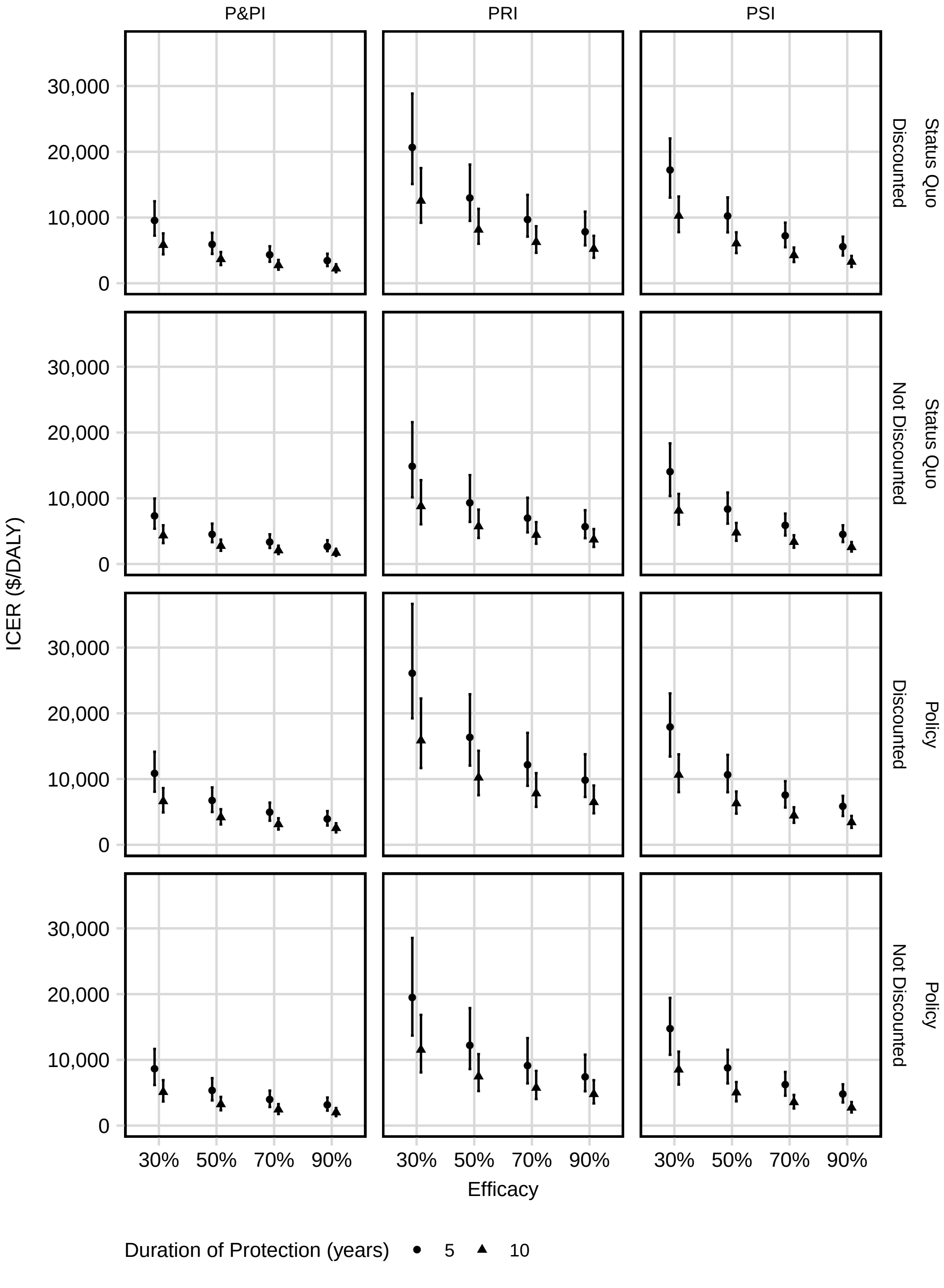

Figure S37: ICER (USD per DALY averted) for vaccination in China over 2027–2050 at USD 10 per vaccine. Points represent median value. Bars represent the uncertainty interval. P&PI: pre and post-infection efficacy; PSI: post-infection efficacy; PRI: pre-infection efficacy. Horizontal facets show baseline scenarios.

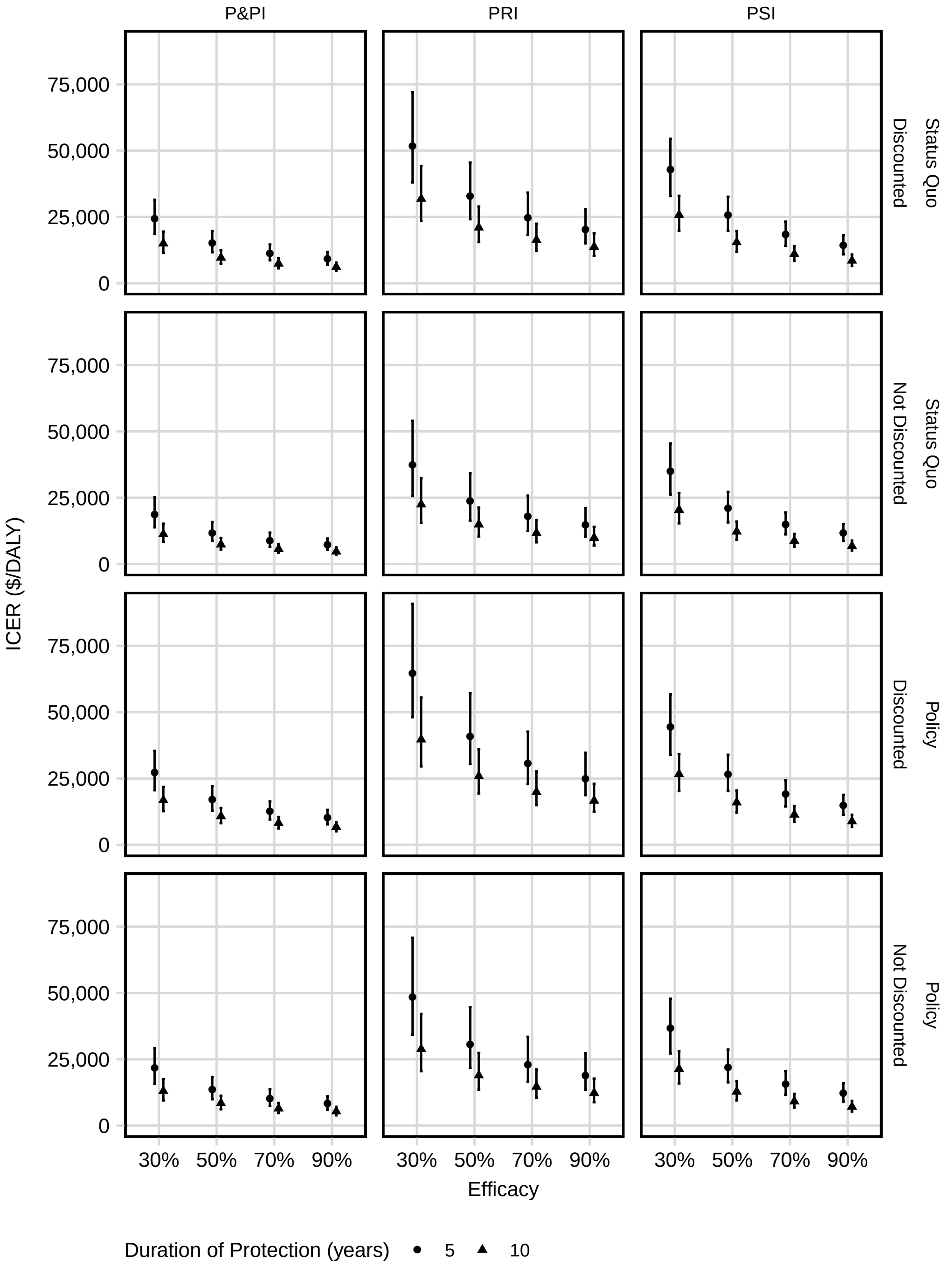

Figure S38: ICER (USD per DALY averted) for vaccination in China over 2027–2050 at USD 30 per vaccine. Points represent median value. Bars represent the uncertainty interval. P&PI: pre and post-infection efficacy; PSI: post-infection efficacy; PRI: pre-infection efficacy. Horizontal facets show baseline scenarios.

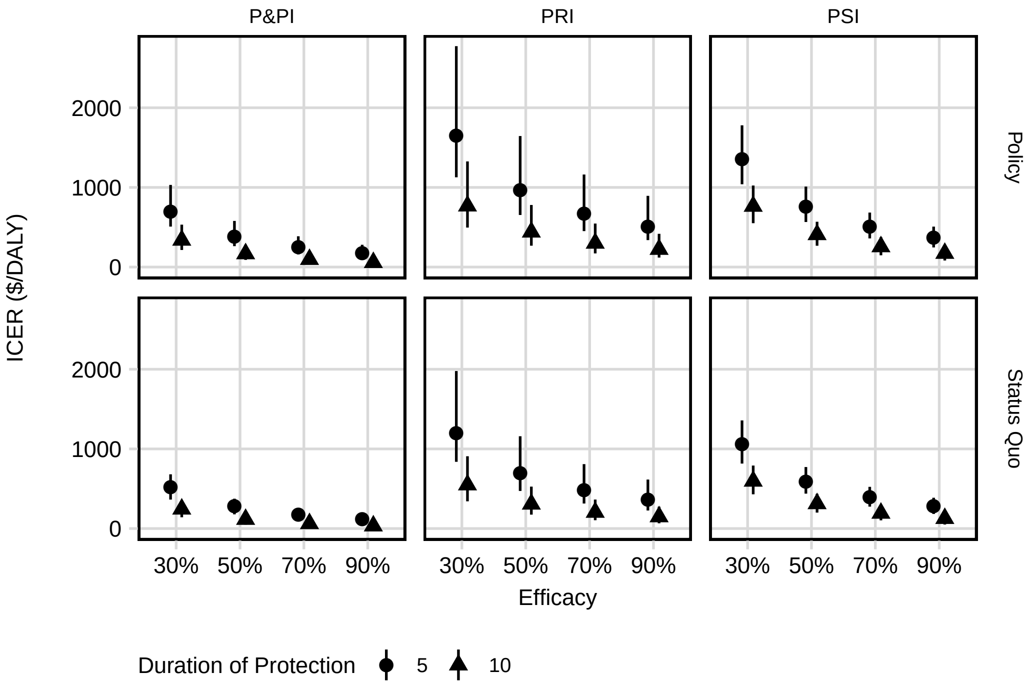

Figure S39: ICER (USD per DALY averted) for vaccination in India over 2027–2050 at USD 10 per vaccine and 30% mass campaign coverage. Points represent median value. Bars represent the uncertainty interval. P&PI: pre and post-infection efficacy; PSI: post-infection efficacy; PRI: pre-infection efficacy. Horizontal facets show baseline scenarios.

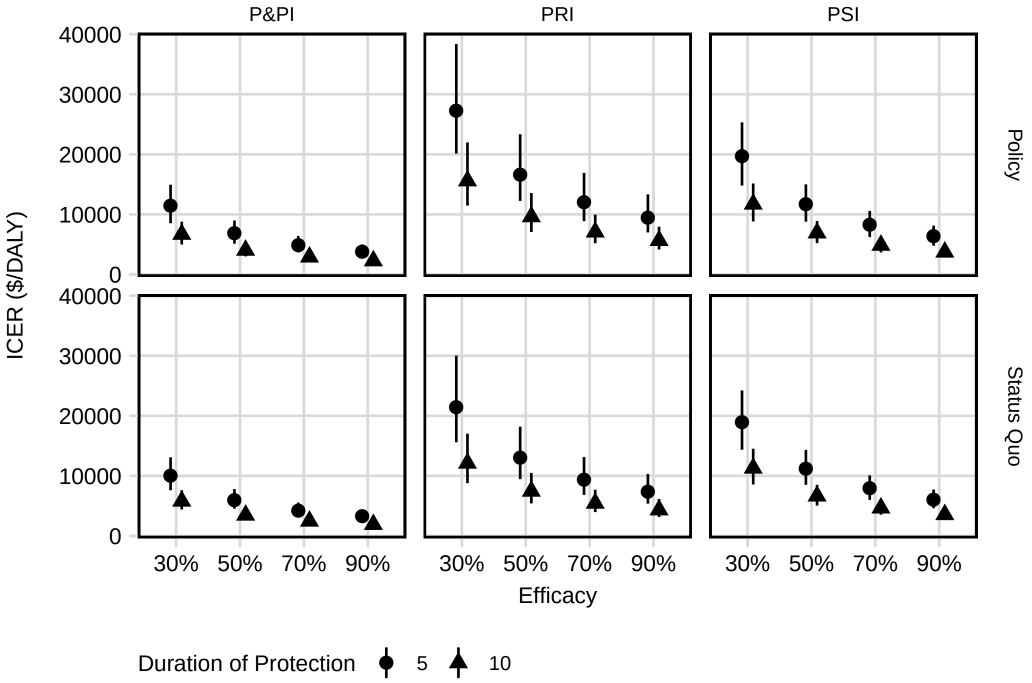

Figure S40: ICER (USD per DALY averted) for vaccination in China over 2027–2050 at USD 10 per vaccine and 30% mass campaign coverage. Points represent median value. Bars represent the uncertainty interval. P&PI: pre and post-infection efficacy; PSI: post-infection efficacy; PRI: pre-infection efficacy. Horizontal facets show baseline scenarios.

9 Budget Impact

For the immunisation programme, we present the total costs incurred through instantaneous deployment of the vaccine. In India, for a 50% efficacy, 10-year duration of protection PSI vaccine priced at USD 10, the model predicted costs for instantly deployed mass campaigns in 2027, 2037 and 2047 of USD 10.2 billion, USD 11.2 billion and USD 12.0 billion respectively. In China, for a 50% efficacy, 10-year duration of protection P&PI vaccine priced at USD 10, the model predicted slightly higher costs for instantly deployed mass campaigns in 2027, 2037 and 2047 of USD 12.7 billion, USD 12.8 billion and USD 12.6 billion, respectively. The corresponding median cost for routine annual vaccination was US$218.2 million (UI: 197.4–229.1) and US$138.1 million (UI: 131.7–166.1) in India and China respectively.

References

1. R Core Team. R: A Language and Environment for Statistical Computing. Vienna, Austria: R Foundation for Statistical Computing, 2019. URL: https://www.R-project.org/.
2. Sensi P. History of the Development of Rifampin. Clin. Infect. Dis. Supplement_3 1983;5:S402–S406.
3. Murray JF, Schraufnagel DE, and Hopewell PC. Treatment of Tuberculosis. A Historical Perspective. Ann. Am. Thorac. Soc. 2015;12:1749–59.
4. Fox W, Ellard GA, and Mitchison DA. Studies on the Treatment of Tuberculosis Undertaken by the British Medical Research Council Tuberculosis Units, 1946-1986, with Relevant Subsequent Publications. Int. J. Tuberc. Lung Dis. 10 Suppl 2 1999;3:S231–279.
5. Menzies D, Benedetti A, Paydar A, et al. Effect of Duration and Intermittency of Rifampin on Tuberculosis Treatment Outcomes: A Systematic Review and Meta-Analysis. PLOS Medicine 2009;6:e1000146.
6. Johnston JC, Campbell JR, and Menzies D. Effect of Intermittency on Treatment Outcomes in Pulmonary Tuberculosis: An Updated Systematic Review and Metaanalysis. Clin Infect Dis 2017;64:1211–20.
7. Schaaf HS, Collins A, Bekker A, and Davies PDO. Tuberculosis at Extremes of Age. Respirology 2010;15:747–63.
8. Swaminathan S and Rekha B. Pediatric Tuberculosis: Global Overview and Challenges. Clin Infect Dis Supplement_3 2010;50:S184–S194.
9. Datta M and Swaminathan S. Global Aspects of Tuberculosis in Children. Paediatric Respiratory Reviews 2001;2:91–6.
10. Rajagopalan S and Yoshikawa TT. Tuberculosis in the Elderly. Z Gerontol Geriatr 2000;33:374–80.
11. Harris RC, Sumner T, Knight GM, et al. Age-Targeted Tuberculosis Vaccination in China and Implications for Vaccine Development: A Modelling Study. The Lancet Global Health 2019;7:e209–e218.
12. Knight GM, Griffiths UK, Sumner T, et al. Impact and Cost-Effectiveness of New Tuberculosis Vaccines in Low- and Middle-Income Countries. PNAS 2014;111:15520–5.
13. Straetemans M, Glaziou P, Bierrenbach AL, Sismanidis C, and van der Werf MJ. Assessing Tuberculosis Case Fatality Ratio: A Meta-Analysis. PLoS ONE 2011;6. Ed. by Pai M:e20755.
14. Tiemersma EW, Werf MJ van der, Borgdorff MW, Williams BG, and Nagelkerke NJD. Natural History of Tuberculosis: Duration and Fatality of Untreated Pulmonary Tuberculosis in HIV Negative Patients: A Systematic Review. PLOS ONE 2011;6:e17601.
15. Cohen T, Sommers B, and Murray M. The Effect of Drug Resistance on the Fitness of Mycobacterium Tuberculosis. The Lancet Infectious Diseases 2003;3:13–21.
16. Cohen T and Murray M. Modeling Epidemics of Multidrug-Resistant M. Tuberculosis of Heterogeneous Fitness. Nat Med 2004;10:1117–21.
17. Knight GM, Zimic M, Funk S, Gilman RH, Friedland JS, and Grandjean L. The Relative Fitness of Drug-Resistant Mycobacterium Tuberculosis: A Modelling Study of Household Transmission in Peru. J. R. Soc. Interface 2018;15:20180025.
18. Kendall EA, Azman AS, Cobelens FG, and Dowdy DW. MDR-TB Treatment as Prevention: The Projected Population-Level Impact of Expanded Treatment for Multidrug-Resistant Tuberculosis. PLOS ONE 2017;12:e0172748.
19. World Health Organization. WHO Tuberculosis Database. 2018. URL: http://www.who.int/tb/country/data/download/en/ (visited on 08/23/2018).
20. Satyanarayana S, Nair SA, Chadha SS, et al. From Where Are Tuberculosis Patients Accessing Treatment in India? Results from a Cross-Sectional Community Based Survey of 30 Districts. PLOS ONE 2011;6:e24160.
21. Arinaminpathy N, Batra D, Khaparde S, et al. The Number of Privately Treated Tuberculosis Cases in India: An Estimation from Drug Sales Data. Lancet Infect Dis 2016;16:1255–60.

22. World Health Organization. Global Tuberculosis Report 2019. Geneva, Switzerland: World Health Organization, 2019.

23. Yeole RD, Khillare K, Chadha VK, Lo T, and Kumar AMV. Tuberculosis Case Notification by Private Practitioners in Pune, India: How Well Are We Doing? Public Health Action 2015;5:173–9.

24. Wang L, Zhang H, Ruan Y, et al. Tuberculosis Prevalence in China, 1990–2010; a Longitudinal Analysis of National Survey Data. The Lancet 2014;383:2057–64.

25. Ministry of Health and Family Welfare, Government of India and World Health Organization. Report of the First National Anti-Tuberculosis Drug Resistance Survey India. 2018. URL: `https://tbcindia.gov.in/WriteReadData/l892s/4187947827National%20Anti-TB%20Drug%20Resistance%20Survey.pdf` (visited on 04/06/2018).

26. Ministry of Health and Family Welfare, Government of India. National Strategic Plan for Tuberculosis Elimination 2017-25. New Delhi, India: Government of India, 2017. URL: `https://tbcindia.gov.in/WriteReadData/NSP%20Draft%2020.02.2017%201.pdf` (visited on 09/25/2018).

27. Lin HH, Wang L, Zhang H, Ruan Y, Chin DP, and Dye C. Tuberculosis Control in China: Use of Modelling to Develop Targets and Policies. Bull. World Health Organ. 2015;93:790–8.

28. United Nations Department of Economic and Social Affairs, Population Division. World Population Prospects. Vol. 1. 2 vols. New York: United Nations, 2019.

29. Read JM, Lessler J, Riley S, et al. Social Mixing Patterns in Rural and Urban Areas of Southern China. Proc. R. Soc. Lond. B Biol. Sci. 2014;281:20140268.

30. Mossong J, Hens N, Jit M, et al. Social Contacts and Mixing Patterns Relevant to the Spread of Infectious Diseases. PLOS Medicine 2008;5:e74.

31. Dye C and Williams BG. Eliminating Human Tuberculosis in the Twenty-First Century. J. R. Soc. Interface 2008;5:653–62.

32. Abu-Raddad LJ, Sabatelli L, Achterberg JT, et al. Epidemiological Benefits of More-Effective Tuberculosis Vaccines, Drugs, and Diagnostics. Proc. Natl. Acad. Sci. U. S. A. 2009;106:13980–5.

33. Dye C, Garnett GP, Sleeman K, and Williams BG. Prospects for Worldwide Tuberculosis Control under the WHO DOTS Strategy. The Lancet 1998;352:1886–91.

34. Vynnycky E and Fine PE. The Natural History of Tuberculosis: The Implications of Age-Dependent Risks of Disease and the Role of Reinfection. Epidemiol Infect 1997;119:183–201.

35. Gomes GM, Rodrigues P, Hilker FM, et al. Implications of Partial Immunity on the Prospects for Tuberculosis Control by Post-Exposure Interventions. J. Theor. Biol. 2007;248:608–17.

36. Schulzer M, Fitzgerald JM, Enarson DA, and Grzybowski S. An Estimate of the Future Size of the Tuberculosis Problem in Sub-Saharan Africa Resulting from HIV Infection. Tuber. Lung Dis. Off. J. Int. Union Tuberc. Lung Dis. 1992;73:52–8.

37. Yoshikawa TT. Tuberculosis in Aging Adults. J Am Geriatr Soc 1992;40:178–87.

38. Marion C and High K. Tuberculosis in Older Adults. In: Yoshikawa T and Norman D. *Infectious Disease in the Aging: A Clinical Handbook*. Springer Science & Business Media, 2009. Google Books: `xg7xVJwvycwC`.

39. Ferebee SH. Controlled Chemoprophylaxis Trials in Tuberculosis. A General Review. Bibl. Tuberc. 1970;26:28–106.

40. Marx FM, Dunbar R, Enarson DA, et al. The Temporal Dynamics of Relapse and Reinfection Tuberculosis After Successful Treatment: A Retrospective Cohort Study. Clin Infect Dis 2014;58:1676–83.

41. Perriëns JH, St. Louis ME, Mukadi YB, et al. Pulmonary Tuberculosis in HIV-Infected Patients in Zaire — A Controlled Trial of Treatment for Either 6 or 12 Months. N. Engl. J. Med. 1995;332:779–85.

42. Batool Sharifi-Mood, Malihe Metanat, Roya Alavi-Naini, et al. The Comparison of Six-Month and Four-Month Regimens of Chemotherapy in the Treatment of Smear Positive Pulmonary Tuberculosis. J. Med. Sci. 2006;6:108–11.

43. Felten MK. Importance of Rifampicin in Combined Daily/Intermittent Chemotherapy for Tuberculosis. S. Afr. Med. J. 1989;75:524–6.

44. Combs DL. USPHS Tuberculosis Short-Course Chemotherapy Trial 21: Effectiveness, Toxicity, and Acceptability: The Report of Final Results. Ann. Intern. Med. 1990;112:397.

45. Holtz TH, Sternberg M, Kammerer S, et al. Time to Sputum Culture Conversion in Multidrug-Resistant Tuberculosis: Predictors and Relationship to Treatment Outcome. Ann. Intern. Med. 2006;144:650.

46. World Health Organization. Guidelines for Treatment of Drug-Susceptible Tuberculosis and Patient Care. World Health Organization, 2017. URL: `https://apps.who.int/iris/handle/10665/255052` (visited on 05/08/2020).

47. World Health Organization. WHO Consolidated Guidelines on Drug-Resistant Tuberculosis Treatment. 2019. URL: `http://www.ncbi.nlm.nih.gov/books/NBK539517/` (visited on 03/31/2020).

48. Xu C, Pang Y, Li R, et al. Clinical Outcome of Multidrug-Resistant Tuberculosis Patients Receiving Standardized Second-Line Treatment Regimen in China. Journal of Infection 2018;0.

49. World Health Organization. Global Tuberculosis Report 2016. 2016.

50. Harris RC. Informing Development Strategies for New Tuberculosis Vaccines: Mathematical Modelling and Novel Epidemiological Tools. Doctoral Thesis. London: London School of Hygiene & Tropical Medicine, 2018. 480 pp. URL: `http://researchonline.lshtm.ac.uk/4648987/` (visited on 09/02/2018).

51. Funk S. Socialmixr. 2020. URL: `https://github.com/sbfnk/socialmixr` (visited on 02/11/2020).

52. Sunnåker M, Busetto AG, Numminen E, Corander J, Foll M, and Dessimoz C. Approximate Bayesian Computation. PLOS Computational Biology 2013;9:e1002803.

53. Wegmann D, Leuenberger C, and Excoffier L. Efficient Approximate Bayesian Computation Coupled With Markov Chain Monte Carlo Without Likelihood. Genetics 2009;182:1207–18.

54. Pandey S, Chadha VK, Laxminarayan R, and Arinaminpathy N. Estimating Tuberculosis Incidence from Primary Survey Data: A Mathematical Modeling Approach. Int J Tuberc Lung Dis 2017;21:366–74.

55. Shen X, DeRiemer K, Yuan Z, et al. Deaths among Tuberculosis Cases in Shanghai, China: Who Is at Risk? BMC Infect Dis 2009;9:95.

56. Wang WB, Zhao Q, Yuan ZA, Jiang WL, Liu ML, and Xu B. Deaths of Tuberculosis Patients in Urban China: A Retrospective Cohort Study. Int. J. Tuberc. Lung Dis. 2013;17:493–8.

57. Cheng J, Wang L, Zhang H, and Xia Y. Diagnostic Value of Symptom Screening for Pulmonary Tuberculosis in China. PLoS One 2015;10.

58. Glaziou P, Dodd PJ, Zignol M, Sismanidis K, and Floyd K. Online Technical Appendix: WHO Global Tuberculosis Report 2018. URL: `https://www.who.int/tb/publications/global_report/gtbr2018_online_technical_appendix_global_disease_burden_estimation.pdf?ua=1` (visited on 02/12/2020).

59. Zhao Y, Xu S, Wang L, et al. National Survey of Drug-Resistant Tuberculosis in China. N Eng J Med 2012;366:2161–70.

60. World Health Organisation. Global Tuberculosis Report 2017. Geneva, Switzerland: World Health Organizaton, 2017. URL: `http://apps.who.int/iris/bitstream/10665/259366/1/9789241565516-eng.pdf` (visited on 11/21/2017).

61. Dodd PJ, Sismanidis C, and Seddon JA. Global Burden of Drug-Resistant Tuberculosis in Children: A Mathematical Modelling Study. The Lancet Infectious Diseases 2016;16:1193–201.

62. Tait DR, Hatherill M, Van Der Meeren O, et al. Final Analysis of a Trial of M72/AS01E Vaccine to Prevent Tuberculosis. N. Engl. J. Med. 2019;381:2429–39.

63. Nemes E, Geldenhuys H, Rozot V, et al. Prevention of M. Tuberculosis Infection with H4:IC31 Vaccine or BCG Revaccination. N. Engl. J. Med. 2018;379:138–49.

64. World Health Organization. WHO Preferred Product Characteristics for New Tuberculosis Vaccines. Technical documents WHO/IVB/18.06. World Health Organization, 2018. 26 pp. URL: `http://apps.who.int/iris/handle/10665/273089` (visited on 11/16/2018).

65. HPV Information Centre. Human Papillomavirus and Related Diseases Report. Institut Català d'Oncologia, 2017. URL: http://www.hpvcentre.net/statistics/reports/ZAF.pdf.

66. UNESCO Institute for Statistics. Education: Gross Enrolment Ratio by Level of Education. UIS.Stat. URL: http://data.uis.unesco.org/?queryid=142.

67. Harouna Djingarey M. Roll out of the Meningococcal A Conjugate Vaccine through Mass Vaccination Campaigns in Countries of the African Meningitis Belt. Meeting of the Strategic Advisory Group of Experts on Immunization (SAGE) (Geneva, Switzerland). 2014.

68. Wu S, Yang P, Li H, Ma C, Zhang Y, and Wang Q. Influenza Vaccination Coverage Rates among Adults before and after the 2009 Influenza Pandemic and the Reasons for Non-Vaccination in Beijing, China: A Cross-Sectional Study. BMC Public Health 2013;13:636.

69. Zheng Y, Yang P, Wu S, et al. A Cross-Sectional Study of Factors Associated with Uptake of Vaccination against Influenza among Older Residents in the Postpandemic Season in Beijing, China. BMJ Open 2013;3:e003662.

70. Das BR, Kakoti G, Bahety H, Das N, and Medhi AH. Adult Japanese Encephalitis Mass Vaccination Campaign: A Rapid Convenience Assessment. Int. J. Curr. Res. Acad. Rev. 2014;2:30–6.

71. Vassall A, Sweeney S, Kahn J, et al. Reference Case for Estimating the Costs of Global Health Services and Interventions. Global Health Cost Consortium, 2017. URL: https://ghcosting.org/pages/standards/reference_case (visited on 04/16/2020).

72. Mijiti P, Yuehua L, Feng X, et al. Prevalence of Pulmonary Tuberculosis in Western China in 2010–11: A Population-Based, Cross-Sectional Survey. Lancet Glob. Health 2016;4:e485–e494.

73. Chatterjee S, Das P, Nigam A, et al. Variation in Cost and Performance of Routine Immunisation Service Delivery in India. BMJ Glob. Health 2018;3.

74. Department of Health and Family Welfare, Ministry of Health and Family Welfare. Introduction of Measles-Rubella Vaccine National Operational Guidelines. New Delhi, India, 2017. URL: https://main.mohfw.gov.in/sites/default/files/195431585071489665073.pdf (visited on 05/01/2020).

75. Yu W, Lu M, Wang H, et al. Routine Immunization Services Costs and Financing in China, 2015. Vaccine 2018;36:3041–7.

76. Yin Z, Beeler Asay GR, Zhang L, et al. An Economic Evaluation of the Use of Japanese Encephalitis Vaccine in the Expanded Program of Immunization of Guizhou Province, China. Vaccine 2012;30:5569–77.

77. Hutton DW, So SK, and Brandeau ML. Cost-Effectiveness of Nationwide Hepatitis B Catch-up Vaccination among Children and Adolescents in China. Hepatology 2010;51:405–14.

78. Floyd K, Arora VK, Murthy KJR, et al. Cost and Cost-Effectiveness of PPM-DOTS for Tuberculosis Control: Evidence from India. Bull World Health Organ 2006;84:437–45.

79. Pantoja A, Lönnroth K, Lal SS, et al. Economic Evaluation of Public-Private Mix for Tuberculosis Care and Control, India. Part II. Cost and Cost-Effectiveness. Int. J. Tuberc. Lung Dis. 2009;13:705–12.

80. Rupert S, Vassall A, Raizada N, et al. Bottom-up or Top-down: Unit Cost Estimation of Tuberculosis Diagnostic Tests in India. Int. J. Tuberc. Lung Dis. 2017;21:375–80.

81. Department of Community Medicine & School of Public Health, Post Graduate Institute of Medical Education and Research (PGIMER) Chandigarh, Department for Health Research, Ministry of Health & Family Welfare, Health Technology Assessment in India, Public Health Foundation of India, Indian Institute of Techology, Madras, and Tata Institute of Social Science. National Health System Cost Database for India. National Health System Cost Database for India. URL: https://www.healtheconomics.pgisph.in/costing_web/index.php?action=Cost_data (visited on 03/31/2020).

82. Stop TB Partnership. Medicines & Diagnostics Catalog - Global Drug Facility. Geneva, Switzerland: Stop TB Partnership, 2020:64.

83. Gotham D, Fortunak J, Pozniak A, et al. Estimated Generic Prices for Novel Treatments for Drug-Resistant Tuberculosis. J Antimicrob Chemother 2017;72:1243–52.

84. World Health Organization. WHO-CHOICE. WHO. URL: http://www.who.int/choice/en/ (visited on 02/18/2020).

85. Fitzpatrick C, Hui Z, Lixia W, et al. Cost–Effectiveness of a Comprehensive Programme for Drug-Resistant Tuberculosis in China. Bull World Health Organ 2015;93:775–84.

86. Wang G, Wang S, Jiang G, Fu Y, Shang Y, and Huang H. Incremental Cost-Effectiveness of the Second Xpert MTB/RIF Assay to Detect Mycobacterium Tuberculosis. J Thorac Dis 2018;10:1689–95.

87. Salomon JA, Haagsma JA, Davis A, et al. Disability Weights for the Global Burden of Disease 2013 Study. The Lancet Global Health 2015;3:e712–e723.

88. World Health Organization. WHO | Metrics: Disability-Adjusted Life Year (DALY). Health Statistics and Information Systems. 2014. URL: http://www.who.int/healthinfo/global_burden_disease/metrics_daly/en/ (visited on 08/22/2018).

89. Claxton KP, Revill P, Sculpher M, Wilkinson T, Cairns J, and Briggs A. The Gates Reference Case for Economic Evaluation. Seattle WA USA Bill Melinda Gates Found. 2014.

90. World Health Organization. Gear up to End TB: Introducing the End TB Strategy. Technical documents WHO/HTM/GTB/2015.09. World Health Organization, 2015. 20 pp. URL: http://apps.who.int/iris/handle/10665/156394 (visited on 11/15/2018).

91. Lönnroth K and Raviglione M. The WHO's New End TB Strategy in the Post-2015 Era of the Sustainable Development Goals. Trans R Soc Trop Med Hyg 2016;110:148–50.

92. WHO Commission on Macroeconomics and Health. Macroeconomics and health : investing in health for economic development : executive summary. Geneva, Switzerland: World Health Organization, 2001. URL: https://apps.who.int/iris/handle/10665/42463 (visited on 02/20/2020).

93. Ochalek J, Lomas J, and Claxton K. Estimating Health Opportunity Costs in Low-Income and Middle-Income Countries: A Novel Approach and Evidence from Cross-Country Data. BMJ Glob. Health 2018;3:e000964.
